# Strongly Luminescent Pt(IV) Complexes with a Mesoionic *N*-Heterocyclic Carbene Ligand: Tuning Their Photophysical Properties

**DOI:** 10.1021/acs.inorgchem.1c00410

**Published:** 2021-05-10

**Authors:** Ángela Vivancos, Adrián Jiménez-García, Delia Bautista, Pablo González-Herrero

**Affiliations:** † Departamento de Química Inorgánica, Facultad de Química, Universidad de Murcia, Campus de Espinardo 19, 30100 Murcia, Spain; ‡ Área Científica y Técnica de Investigación, Universidad de Murcia, Campus de Espinardo, 21, 30100 Murcia, Spain

## Abstract

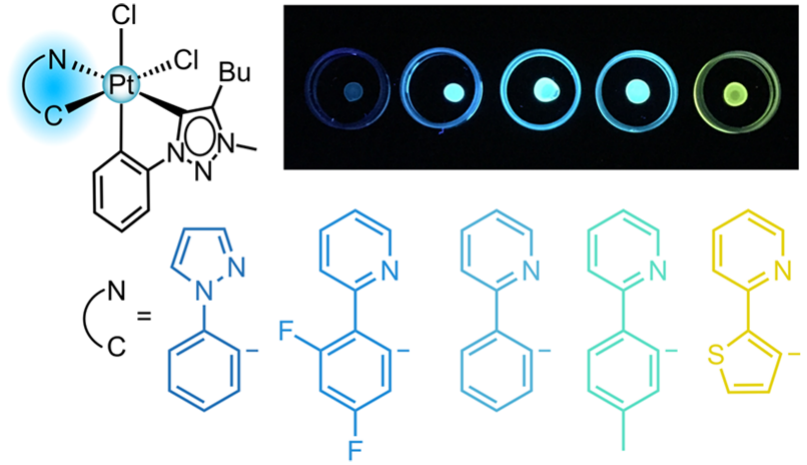

The synthesis, electrochemistry, and photophysical properties of a series of bis-cyclometalated Pt(IV) complexes that combine the mesoionic aryl-NHC ligand 4-butyl-3-methyl-1-phenyl-1*H*-1,2,3-triazol-5-ylidene (trz) with either 1-phenylpyrazole or 2-arylpyridine (C^∧^N) are reported. The complexes (*OC*-6-54)-[PtCl_2_(C^∧^N)(trz)] bearing cyclometalating 2-arylpyridines present phosphorescent emissions in the blue to yellow color range, which essentially arise from ^3^LC(C^∧^N) states, and reach quantum yields of ca. 0.3 in fluid solutions and almost unity in poly(methyl methacrylate) (PMMA) matrices at 298 K, thus representing a class of strong emitters with tunable properties. A systematic comparison with the homologous *C*_2_-symmetrical species (*OC*-6-33)-[PtCl_2_(C^∧^N)_2_], which contains two equal 2-arylpyridine ligands, shows that the introduction of a trz ligand leads to significantly lower nonradiative decay rates and higher quantum efficiencies. Computational calculations substantiate the effect of the carbene ligand, which raises the energy of dσ* orbitals in these derivatives and results in the higher energies of nonemissive deactivating ^3^LMCT states. In contrast, the isomers (*OC*-6-42)-[PtCl_2_(C^∧^N)(trz)] are not luminescent because they present a ^3^LMCT state as the lowest triplet.

## Abstract 2

Bis-cyclometalated Pt(IV) complexes bearing an aryl-1,2,3-triazolylidene ligand and heteroaromatic C^∧^N ligands with different energies for the lowest π−π* transition are reported, constituting a class of strongly phosphorescent compounds with tunable properties. A comparison with bis-cyclometalated complexes bearing only C^∧^N ligands provides the first systematic demonstration of the electronic effects of a cyclometalating aryl-NHC ligand on the luminescence of Pt(IV) complexes.

## Introduction

Transition-metal complexes featuring long-lived emissive triplet excited states are at the core of numerous technological, analytical, biomedical, and synthetic developments, including chemosensing,^1,2^ cell imaging,^3^ photodynamic therapy,^4^ photocatalysis,^5^ and light-emitting materials.^6−8^ Over the past decades, most research in this area has focused on luminescent Ir(III)^8−13^ and Pt(II)^14−18^ complexes with cyclometalating heteroaromatic ligands because of the high tunability and adaptability of their excited states, whereas Pt(IV) complexes have only started to be systematically explored as strong emitters in recent years.^19−27^

In previous contributions, we have shown that several types of Pt(IV) complexes bearing cyclometalating 2-arylpyridines may exhibit efficient and long-lived luminescence from essentially ligand-centered triplet excited states (^3^LC) that possess a very low metal-to-ligand charge-transfer (MLCT) admixture.^19,20,22,23,27^ These characteristics make them promising candidates for applications that take advantage of relatively long excited-state lifetimes, such as sensing, singlet-oxygen sensitization, or photocatalysis. Although small, the extent of the MLCT contribution to the emissive state has been observed to fluctuate depending on the coordination environment, causing variations in the radiative rates.^21,22,28^ Thus, shorter Pt–C bonds from metalated aryls or the presence of suitable π-donor ancillary ligands, e.g., the fluoride ion, result in occupied dπ orbitals with higher energies and greater MLCT admixtures, leading to higher radiative rates.^21^ However, a more critical factor that influences the emission properties of cyclometalated Pt(IV) complexes is the presence of thermally accessible ligand-to-metal charge-transfer (LMCT) excited states originating from electronic promotions to dσ* orbitals, which can provide the effective nonradiative deactivation of the emissive excited state. Higher-energy LMCT states can be achieved by introducing strong σ-donor ligands, which usually lead to lower nonradiative rates and increased emission efficiencies.^23^

*N*-Heterocyclic carbenes (NHCs) have emerged as very valuable ligands for the design of highly efficient luminescent complexes of late-transition-metal ions, such as Ir(III),^29,30,39,31−38^ Pt(II),^40,41,50,42−49^ or Au(III).^51^ The beneficial effects exerted by these ligands can be attributed to their exceptional σ-donor capabilities,^52,53^ which lead to strong ligand–field splittings and an increased energy of nonemissive excited states that arise from electronic transitions to dσ* orbitals, which could otherwise become thermally populated and cause nonradiative deactivation or even degradation via ligand–metal σ-bond labilization. Consequently, the use of NHCs brings about improved stabilities and emission efficiencies, which are particularly important for the development of blue emitters. Diverse types of NHC ligands have been used to synthesize luminescent complexes, including chelating dicarbenes (C*^∧^C*),^54−56^ pyridyl-NHCs (N^∧^C*),^57,58^ and cyclometalated aryl-NHCs (C^∧^C*).^43,50,59^ Most incorporate normal Arduengo-type NHC moieties, whereas the use of mesoionic NHCs is rather infrequent.^46,47,60−62^ Although cyclometalating aryl-NHCs have been demonstrated as chromophoric ligands in homoleptic Ir(III) emitters,^37−39^ mixed-ligand systems have also been developed in which they act as supporting ligands while other chelating heteroaromatic ligands, such as arylpyridines^34,63^ or bipyridines,^32^ are responsible for the emission.

We have recently developed a synthetic method that allowed the preparation of the first examples of Pt(IV) complexes bearing a cyclometalated aryl-NHC ligand.^64^ The reported complexes combined a mesoionic carbene of the 1,2,3-triazolylidene subclass (C^∧^C*) and either a monocyclometalating 2,6-diarylpyridine or a dicyclometalating 2,6-diarylpyridine (C^∧^N^∧^CH or C^∧^N^∧^C, respectively) and were found to display exceptionally intense phosphorescence in poly(methyl methacrylate) (PMMA) matrices at 298 K, which originated from a ^3^LC state involving the C^∧^N^∧^CH or C^∧^N^∧^C ligand. In addition, the complex with a monocyclometalating C^∧^N^∧^CH ligand showed an intense luminescence in a fluid solution, which was marginally enhanced compared to that of similar bis-cyclometalated Pt(IV) complexes containing only C^∧^N ligands. However, no systematic evidence of the effects of supporting C^∧^C* ligands on the emission efficiencies of Pt(IV) complexes has been gathered so far.

In this work, we present a family of bis-cyclometalated Pt(IV) complexes bearing an aryl-1,2,3-triazolylidene ligand and cyclometalating C^∧^N ligands of different energies for the lowest π–π* transition, which exhibit strong phosphorescent emissions in fluid solutions and can reach quantum efficiencies of almost unity in PMMA matrices. Their emission properties are compared to those of bis-cyclometalated complexes bearing only C^∧^N ligands with the aim of providing a clear and general demonstration of the electronic effects of the C^∧^C* ligand.

## Results and Discussion

### Synthesis

**Scheme 1:**
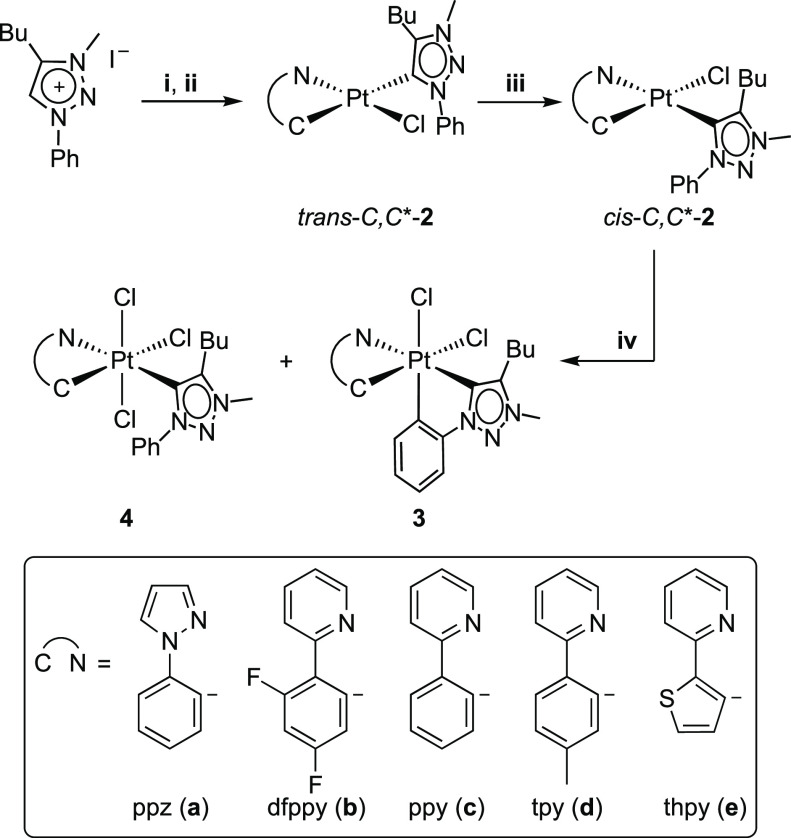
(i) Ag_2_O, (ii) [Pt_2_(μ-Cl)_2_(C^∧^N)_2_] (1), (iii) blue LEDs, and (iv) PhICl_2_.

Scheme 1 shows the synthetic route to the targeted bis-cyclometalated complexes (*OC*-6-54)-[PtCl_2_(C^∧^N)(trz)] (**3a**–**e**), where trz = cyclometalating 4-butyl-3-methyl-1-phenyl-1*H*-1,2,3-triazol-5-ylidene and C^∧^N = cyclometalating 1-phenylpyrazole (ppz, **a**), 2-(2,4-difluorophenyl)pyridine (dfppy, **b**), 2-phenylpyridine (ppy, **c**), 2-(*p*-tolyl)pyridine (tpy, **d**), or 2-(2-thienyl)pyridine (thpy, **e**), based on the previously described methodology for (*OC*-6-54)-[PtCl_2_(dtpyH)(trz)] (dtpyH = monocyclometalating 2,6-di(*p*-tolyl)pyridine).^64^ The reaction of dichlorido-bridged dimers [Pt_2_(μ-Cl)_2_(C^∧^N)_2_] (**1a**–**e**) with the in situ-generated silver carbene “AgI(trzH)” led to the selective formation of complexes trans-C,C*-[PtCl(C^∧^N)(trzH)] (trans-C,C*-**2a**–**e**), where the carbene coordinates in a *trans*-configuration to the metalated aryl of the C^∧^N ligand. The photoisomerization to the corresponding cis-C,C*-**2a**–**e** complexes was achieved by either irradiating acetone solutions of trans-C,C*-**2b**–**e** with visible light (λ = 454 nm) or irradiating a MeCN solution of trans-C,C*-**2a** with UV light (λ = 310 nm). In contrast to the related Pt(II) species *cis-N,N*-[PtCl(C^∧^N)(N^∧^CH)], which undergo a photochemical cyclometalation of the coordinated N^∧^CH ligand to give a bis-cyclometalated Pt(IV) hydride,^65^ the cyclometalation of the coordinated trzH ligand did not occur in any of the studied cases.

**Figure 1:**
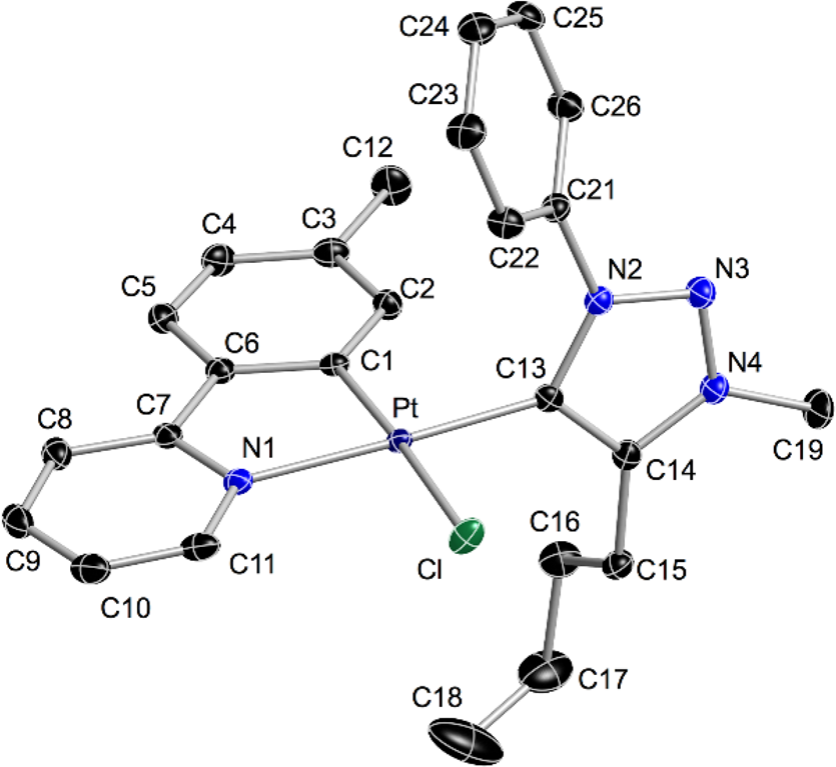
Structure of cis-C,C*-**2d** (thermal ellipsoids at 50% probability). Hydrogen atoms are omitted. Selected bond distances (Å) and angles (°) are as follows: Pt–C1, 1.9825(17); Pt–N1, 2.0669(15); Pt–C13, 1.9670(18); Pt–Cl, 2.4091(5); C1–Pt–N1, 81.13(7); and N1–Pt–C13, 174.67(6).

The photoisomerization process can be easily followed by ^1^H NMR spectrometry because the resonance of the proton *ortho* to the metalated carbon of the C^∧^N ligand is significantly shielded in the *cis*-isomers due to the diamagnetic current of the triazolylidene ring (e.g., 7.83 vs 6.31 ppm for *trans-* and cis-C,C*-**2d**, respectively). Further confirmation of the isomerization was provided by the X-ray diffraction study of complex cis-C,C*-**2d** (Figure 1).

**Scheme 2:**
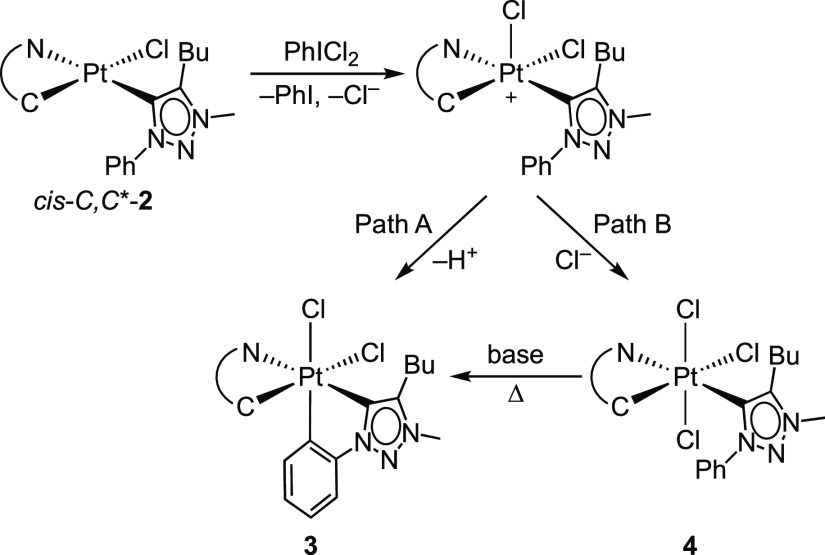


The treatment of cis-C,C*-**2a**–**e** with PhICl_2_ led to the bis-cyclometalated complexes **3a**–**e**, respectively, as the major products in all cases. However, the trichlorido complex (*OC*-6-41)-[PtCl_3_(C^∧^N)(trzH)] (**4a**–**e**) was also formed as a minor product (Scheme 1). This result differs from the reported reactions of the related *cis-* or *trans-N,N*-[PtCl(C^∧^N)(N^∧^CH)] complexes with PhICl_2_, which exclusively led to bis-cyclometalated Pt(IV) complexes.^19,24,66,67^ Complexes **3** and **4** were obtained in different molar ratios depending on the C^∧^N ligand (Table S2). The most favorable outcome was obtained with the dfppy ligand in a 95:5 ratio (**3b**:**4b**), whereas the tpy ligand led to the lowest molar proportion of the bis-cyclometalated complex in a 60:40 ratio (**3d**:**4d**). The formation of these mixtures can be attributed to two competing processes that take place from the pentacoordinate Pt(IV) intermediate arising from the formal addition of a Cl^+^ ion to cis-C,C*-**2a**–**e** (Scheme 2). The electrophilic metalation of the phenyl ring of the trzH ligand (path A) leads to **3**, whereas the coordination of the Cl^–^ ion released from the PhICl_2_ reagent in the vacant coordination site produces **4** (path B). Apparently, the electrophilic metalation is less favored for the present triazolylidene ligand compared to that for *N*-coordinated 2-arylpyridines. The fact that derivative **3b** was obtained in a higher molar proportion can be explained by the higher electrophilic character of the metal center in this case because of the diminished electron-donating ability of the dfppy ligand.

The isolation of complexes **3** from the above mixtures was only possible after several recrystallizations, resulting in low to moderate yields (from 13% for **3d** to 50% for **3b**). Complexes **4** could not be obtained in pure forms except for the tpy derivative **4d**; nevertheless, the ^1^H NMR spectra of enriched fractions allowed us to unequivocally establish their identities. In the case of the tpy derivative **4d**, the considerable shielding of the proton *ortho* to the metalated tolyl carbon (6.71 ppm, *J*_HPt_ = 33 Hz) indicates that it is directed toward the triazolylidene moiety and is affected by its ring current, implying that the mutual disposition of the tpy and carbene ligands is retained after the oxidative addition of PhICl_2_. In view of this configuration, we considered forcing the metalation of the phenyl group of the carbene ligand in complexes **4a** and **4c**–**e** at a high temperature in the presence of a base. Thus, by heating mixtures of **3** and **4** at 130 °C in 1,2-dichlorobenzene in the presence of Na_2_CO_3_, complexes **4** produced the corresponding complexes **3**, which could then be isolated in improved yields (38–64%).

**Figure 2:**
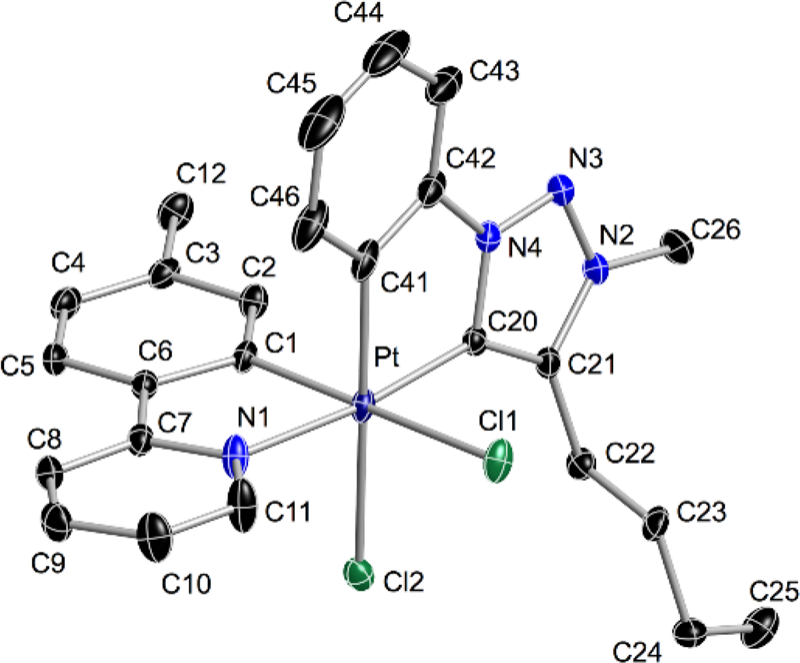
Thermal ellipsoid representation (50% probability) of the crystal structure of **3d**. Hydrogen atoms and solvent molecules are omitted. Selected bond distances (Å) and angles (°) are as follows: Pt–C1, 2.011(2); Pt–N1, 2.0866(19); Pt–C20, 1.987(2); Pt–C41, 2.019(2); Pt–Cl1, 2.4361(6); Pt–Cl2, 2.4295(6); C1–Pt–N1, 80.84(8); C20–Pt–N1, 173.32(8); and C20–Pt–C41, 80.80(10).

**Figure 3:**
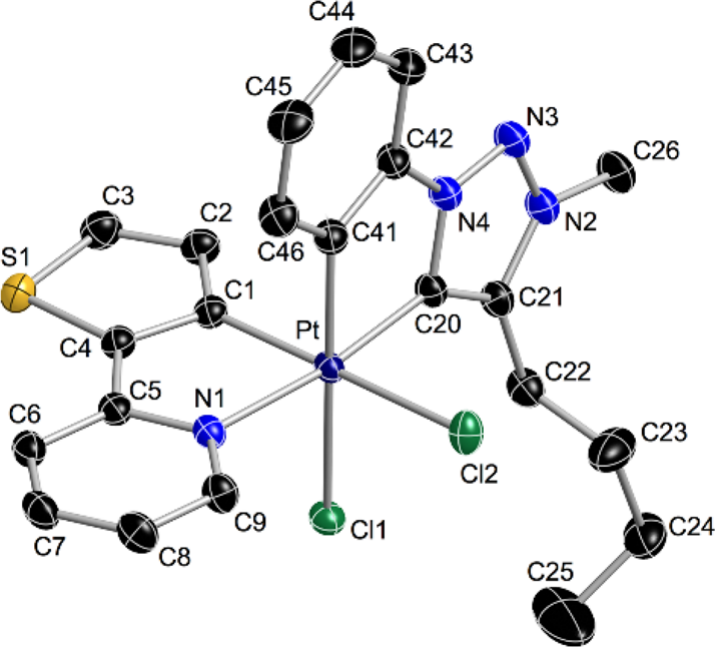
Thermal ellipsoid representation (50% probability) of the crystal structure of **3e**. Hydrogen atoms are omitted. Selected bond distances (Å) and angles (°) are as follows: Pt–C1, 1.992(2); Pt–N1, 2.1031(18); Pt–C20, 1.983(2); Pt–C41, 2.024(2); Pt–Cl1, 2.4227(5); Pt–Cl2, 2.4178(6); C1–Pt–N1, 80.16(8); C20–Pt–N1, 172.66(8); and C20–Pt–C41, 80.77(9).

The ^1^H NMR spectra of complexes **3** corroborated the presence of two metalated aryl groups, each of which gives a considerably shielded resonance flanked by ^195^Pt satellites arising from the proton *ortho* to the metalated carbon, which is affected by the diamagnetic current of an orthogonal ring. The crystal structures of **3d** and **3e** (Figures 2 and 3, respectively) are compatible with the NMR data, further confirming that the metalated aryls are mutually *cis*, while the carbene and pyridine moieties are *trans* to each other.

**Scheme 3:**
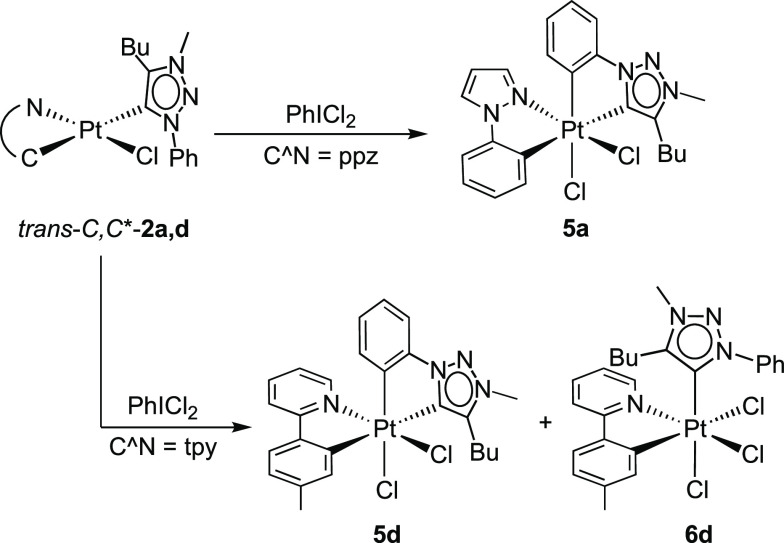


We also attempted the preparation of bis-cyclometalated complexes with a *trans*-arrangement of the carbene and aryl moieties to compare their photophysical properties with those of the isomeric complexes **3**. The reaction of trans-C,C*-**2a** with PhICl_2_ afforded a mixture from which complex (*OC*-6-42)-[PtCl_2_(ppz)(trz)] (**5a**; Scheme 3) could be isolated in a 23% yield, while the other products could not be identified. In the case of trans-C,C*-**2d**, the same reaction gave a mixture of the desired complex (*OC*-6-42)-[PtCl_2_(tpy)(trz)] (**5d**) and the trichlorido complex (*OC*-6-43)-[PtCl_3_(tpy)(trzH)] (**6d**) in a ca. 18:82 molar ratio; the mixture could be separated thanks to their different solubilities in MeOH, and the complexes were isolated in 11 and 67% yields, respectively. The ^1^H NMR spectrum of complex **6d** shows that the protons *ortho* to either the metalated *p*-tolyl or the coordinated N atom are not shielded by the triazolylidene ring, implying that the carbenic carbon is not coplanar with the tpy ligand. Reasonably, the cationic Pt(IV) intermediate complex isomerizes to avoid the *trans*-arrangement of the tolyl and triazolylidene groups, which explains the low yield in **5d**. A similar result was previously found upon the oxidation of an analogous complex bearing monocyclometalating 2,6-di(*p*-tolyl)pyridine with PhICl_2_.^64^

**Figure 4:**
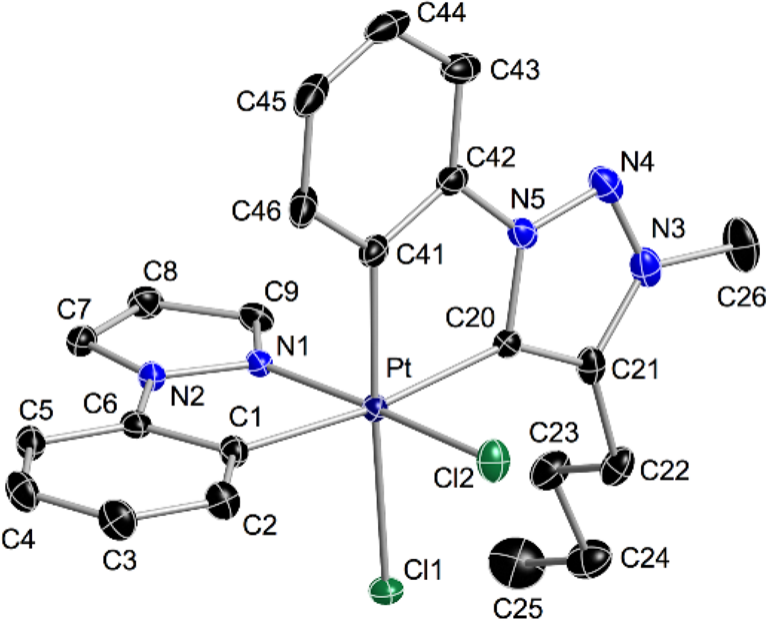
Thermal ellipsoid representation (50% probability) of the crystal structure of **5a**. Hydrogen atoms are omitted. Selected bond distances (Å) and angles (°) are as follows: Pt–C1, 2.050(2); Pt–N1, 2.006(2); Pt–C20, 2.078(2); Pt–C41, 2.025(2); Pt–Cl1, 2.4227(6); Pt–Cl2, 2.3177(6); C1–Pt–N1, 80.09(9); and C20–Pt–C41, 80.71(10).

**Figure 5:**
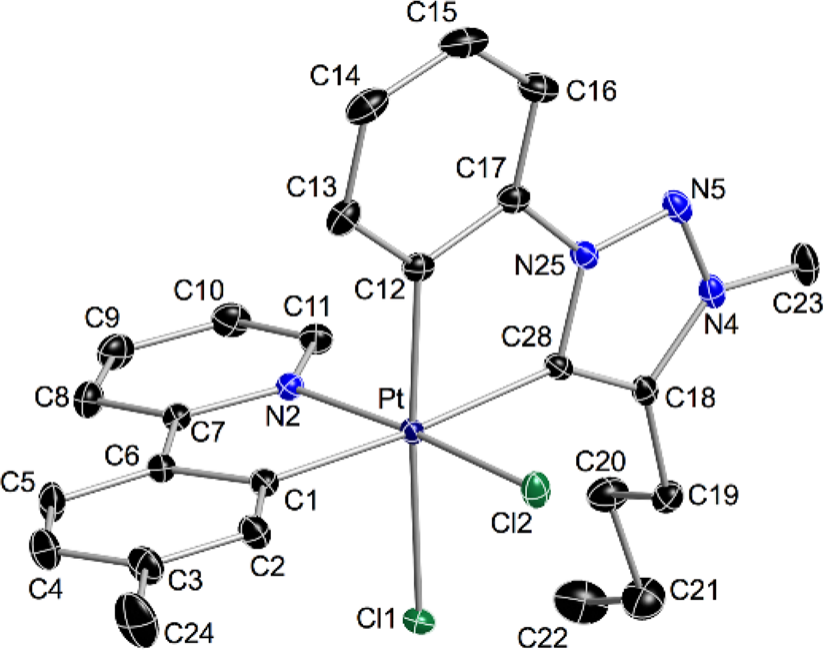
Thermal ellipsoid representation (50% probability) of the crystal structure of **5d**. Hydrogen atoms are omitted. Selected bond distances (Å) and angles (°) are as follows: Pt–C1, 2.0444(15); Pt–N1, 2.0363(13); Pt–C28, 2.0832(15); Pt–C12, 2.0285(15); Pt–Cl1, 2.4196(4); Pt–Cl2, 2.3293(4); C1–Pt–N2, 80.79(6); and C12–Pt–C28, 80.43(6).

The crystal structures of **5a** and **5d** (Figures 4 and 5, respectively) confirmed the expected ligand arrangement. In both cases, the Pt–C* bond is nearly 0.1 Å longer than those in complexes **3** because of the high *trans*-influence exerted by the metalated aryl ring of the C^∧^N ligand. Additionally, significantly longer Pt–C1 bond lengths were found, e.g., 2.044 Å for **5d** vs 2.011 Å for **3d**, as a consequence of the higher *trans*-influence of the triazolylidene ring relative to that of the chlorido ligand.

### Photophysical Properties

**Figure 6:**
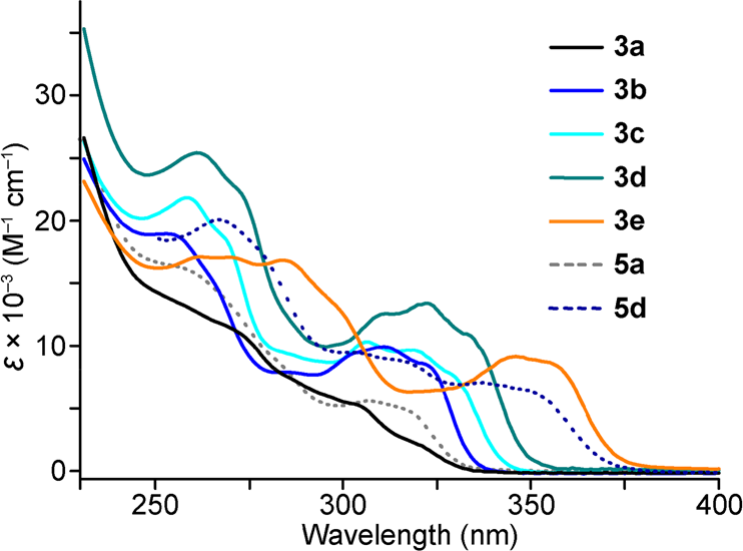
Electronic absorption spectra of complexes **3a**–**e**, **5a**, and **5d** in a CH_2_Cl_2_ solution at 298 K.

The electronic absorption spectra of **3a**–**e**, **5a**, and **5d** were registered in a CH_2_Cl_2_ solution at 298 K (Table 1 and Figure 6). Structured absorption bands are observed for complexes **3a**–**e** in the 250–360 nm range that can be ascribed to essentially ^1^LC transitions involving the cyclometalating ligands.^20,23,24^ The lowest-energy band resembles those observed for complexes [PtMe(Cl)(C^∧^N)_2_];^23^ its lowest maximum shifts from 320 to 354 nm along the sequence **3a** → **3e** in accordance with the decreasing energies of the lowest π–π* transition of the C^∧^N ligands and can therefore be ascribed to a primarily ^1^LC(C^∧^N) excitation. The absorption spectra of **5a** and **5d** differ from their respective isomeric complexes **3a** and **3d** mainly in regard to the lowest-energy feature, which appears to enclose additional absorptions. This is particularly evident for **5d**, whose lowest-energy feature is significantly red-shifted with respect to that of **3d**. We attribute these differences to LMCT transitions on the basis of TDDFT calculations (see below).

**Table 1:** Electronic Absorption Data for the Studied Complexes in a CH_2_Cl_2_ Solution (ca. 5 × 10^–5^ M) at 298 K

complex	λ_max_ (nm) (ε × 10^–2^ (M^–1^ cm^–1^))
**3a**	272 (111), 303 (54), 320 (21)
**3b**	255 (189), 303 (94), 311 (99), 321 (86)
**3c**	259 (218), 269 (sh, 184), 306 (103), 320 (95), 328 (79)
**3d**	262 (254), 271 (sh, 228), 310 (125), 322 (134), 333 (109)
**3e**	260 (170), 269 (171), 283 (168), 297 (sh, 135) 346 (91), 354 (87)
**5a**	264 (179), 299 (86), 314 (43)
**5d**	267 (200), 303 (94), 315 (sh, 87), 336 (71), 347 (65)

**Figure 7:**
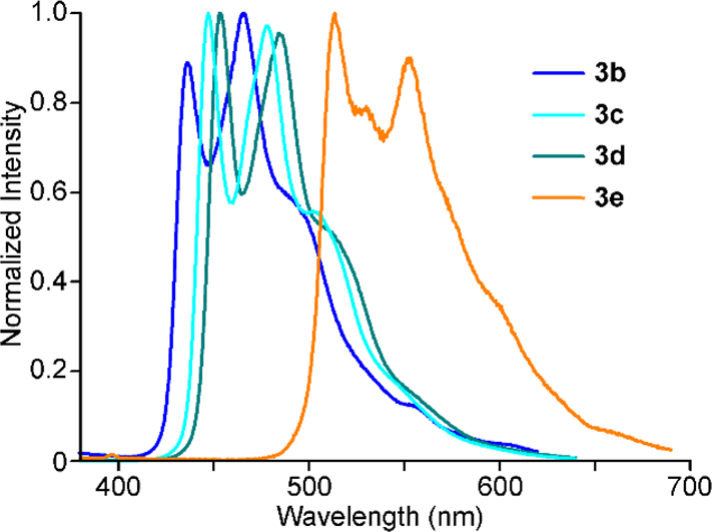
Emission spectra of complexes **3b**–**e** in deaerated CH_2_Cl_2_ solutions at 298 K.

The photoluminescence of **3a**–**e** was examined in a deaerated CH_2_Cl_2_ solution and poly(methyl methacrylate) (PMMA) matrices (2 wt %) at 298 K. Air-equilibrated samples were also examined to evaluate the luminescence quenching of these complexes by atmospheric oxygen. The obtained emission data are compiled in Table 2, and the spectra in the deaerated CH_2_Cl_2_ solution are shown in Figure 7. In all cases, the excitation spectra match the corresponding absorption profiles (Figure S29). The excitation and emission spectra in PMMA are almost identical to those in CH_2_Cl_2_ (Figure S30). In the absence of molecular oxygen, complexes **3b**–**e** show intense emissions in both media, whereas **3a** is not emissive in CH_2_Cl_2_ and only weakly so in PMMA. In all cases, the bands are vibronically structured, and the energy of the highest-energy peak correlates with the triplet emission of the respective N^∧^CH ligand (ppzH, 378 nm;^68^ dfppyH, 424 nm;^69^ ppyH, 430 nm;^70^ tpyH, 437 nm;^69^ and thpyH, 485 nm^70^). Therefore, the C^∧^N ligand is the chromophoric one in all cases, while the cyclometalating trz acts as a supporting ligand. The radiative lifetimes range from tens to hundreds of microseconds, which is consistent with triplet emissive states of an essentially ligand-centered character (^3^LC). The complexes bearing ppy-based ligands (**3b**–**d**) are the most efficient emitters, with quantum yields around 0.3 in solution that increase to almost unity in PMMA matrices; the latter are the highest values ever observed for Pt(IV) complexes. An analysis of their radiative and nonradiative rate constants (*k*_r_ and *k*_nr_, respectively) shows that the large increases in the quantum yields in PMMA are a consequence of the inhibition of molecular motion, which leads to dramatic decreases in the value of *k*_nr_. The significantly weaker emission of the ppz derivative **3a** can be attributed to the thermal population of a nonemissive ^3^LMCT excited state because of the high energy of the ^3^LC(ppz) state; a similar behavior has been reported for the tris-cyclometalated complexes *fac*-[Ir(ppz)_3_]^37^ and *fac*-[Pt(ppz)_3_]^+^.^19^ The lower efficiency of the thpy complex **3e** compared with those of **3b**–**d** can be explained by its lower emission energy, which must result in an increased nonradiative deactivation via a vibrational overlap with the ground state.

**Table 2:** Emission Data of Complexes **3a**–**e**

complex	medium^a^	λ_em_ (nm)^b^	Φ^c^	τ (μs)^d^	*k*_r_ × 10^–3^ (s^–1^)^e^	*k*_nr_ × 10^–3^ (s^–1^)^f^
**3a**	PMMA	417, 467, 487	0.044	46	0.96	21
3b	CH_2_Cl_2_	436, 466, 494 (sh), 554 (sh)	0.27	133	2.0	5.5
	PMMA	435, 464, 493	0.97	295	3.3	0.088
	CH_2_Cl_2_ (air)	436, 465, 495	0.012	4.3	2.8	233
	PMMA (air)	436, 465, 494	0.23	88 (48%), 207 (52%)		
3c	CH_2_Cl_2_	447, 478, 504 (sh)	0.31	110	2.8	6.2
	PMMA	446, 478, 504 (sh)	0.93	258	3.6	0.26
	CH_2_Cl_2_ (air)	448, 478, 503 (sh)	0.009	3.4	2.6	291
	PMMA (air)	447, 478, 504 (sh)	0.23	80 (41%), 178 (59%)		
3d	CH_2_Cl_2_	453, 485, 514 (sh)	0.26	140	1.8	5.3
	PMMA	452, 484, 511 (sh)	0.86	319	2.7	0.43
	CH_2_Cl_2_ (air)	453, 484, 513 (sh)	0.007	2.5	2.8	397
	PMMA (air)	453, 484, 513 (sh)	0.20	88 (52%), 213 (48%)		
**3e**	CH_2_Cl_2_	513, 530, 552	0.046	45	1.0	21
	PMMA	512, 528, 551	0.49	304	1.6	1.7
	PMMA (air)	513, 530, 552	0.11	86 (41%), 196 (39%)		

a Under the exclusion of oxygen, except where noted.

b The most intense peak is italicized.

c Quantum yield.

d Emission lifetime; relative amplitudes are given in parentheses for biexponential decays.

e Radiative rate constant, *k*_r_ = Φ/τ.

f Nonradiative rate constant, *k*_nr_ = (1 – Φ)/τ.

Air-equilibrated samples of **3b**–**d** showed measurable luminescence, with quantum yields around 0.01 in CH_2_Cl_2_ and 0.20 in PMMA. However, no emission could be detected from **3a** in any medium, and **3e** was emissive only in PMMA in the presence of atmospheric oxygen. Lifetimes dropped to a few microseconds in CH_2_Cl_2_. The calculated *k*_r_ values in this medium remained the same order of magnitude as those obtained from deaerated samples, but *k*_nr_ values increased by two orders of magnitude as a consequence of oxygen quenching. Much longer lifetimes were observed for samples in PMMA matrices; however, they could only be fitted to biexponential decays, which is probably because of the inhomogeneous oxygen distribution. The present data show that complexes of this kind could be used for luminescence-based applications in the presence of atmospheric oxygen as well as for the development of oxygen sensors.

**Chart 1:**
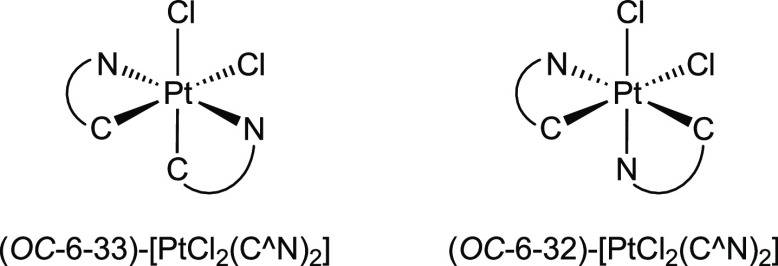


For comparison purposes, the luminescence of *C*_2_-symmetrical complexes (*OC*-6-33)-[PtCl_2_(C^∧^N)_2_] (Chart 1) with C^∧^N = ppz,^19^ dfppy,^19^ ppy,^66^ tpy^19^ and thpy^20^ was also studied in a deaerated CH_2_Cl_2_ solution and PMMA matrices (2 wt %) (Table 3). These compounds show moderate or weak emissions except for the thpy derivative, which was not emissive in solution, and the ppz derivative, which did not show an emission in any medium. The observed emission spectra are almost identical in shape to those of the corresponding complexes **3**, although they are slightly red-shifted (Figures S31 and S32). Single-exponential decays were observed for the ppy-based derivatives in the CH_2_Cl_2_ solution, whereas double-exponential decays were obtained in PMMA. Where possible, comparisons with complexes **3** show that lifetimes are significantly shorter for (*OC*-6-33)-[PtCl_2_(C^∧^N)_2_]. In all cases, the measured quantum yields are much lower than those of complexes **3**, implying that the replacement of one of the C^∧^N ligands by a cyclometalated trz mainly results in an enhancement of the emission efficiencies. This beneficial effect is primarily reflected in the *k*_nr_ values, which are generally one order of magnitude lower for complexes **3** relative to the value of the corresponding (*OC*-6-33)-[PtCl_2_(C^∧^N)_2_] complex, whereas variations in the value of *k*_r_ are much less significant. Reasonably, the stronger σ-donating ability of the carbene compared with that of the C^∧^N ligand pushes the metal dσ*-orbitals to higher energies in complexes **3**, implying both that the deactivating LMCT states lie at higher energies and that their thermal population from the emitting state is more difficult than those in (*OC*-6-33)-[PtCl_2_(C^∧^N)_2_] complexes, leading to lower nonradiative decay rates.

**Table 3:** Emission Data of *C*_2_-Symmetrical Complexes (*OC*-6-33)-[PtCl_2_(C^∧^N)_2_]

C^∧^N	medium^a^	λ_em_ (nm)^b^	Φ^c^	τ (μs)^d^	*k*_*r*_ × 10^–3^ (s^–1^)^e^	*k*_*nr*_ × 10^–3^ (s^–1^)^f^
dfppy	CH_2_Cl_2_	440, *470*, 506 (sh)	0.058	44	1.3	21
	PMMA	439, *469*, 500 (sh)	0.21	76.6 (29%), 218 (71%)		
ppy^g^	CH_2_Cl_2_	*450*, 481, 509 (sh)	0.11	27	4.1	33
	PMMA	*450*, 481, 509 (sh)	0.18	57.0 (28%), 157 (72%)		
tpy	CH_2_Cl_2_	*457*, 489, 524 (sh)	0.15	40	3.8	21
	PMMA	*457*, 487, 521 (sh)	0.22	84.7 (20%), 208 (80%)		
thpy	PMMA	*515*, 530 (sh), 553	0.054	111	0.5	8.5

a Under the exclusion of oxygen.

b The most intense peak is italicized.

c Quantum yield.

d Emission lifetime; relative amplitudes are given in parentheses for biexponential decays.

e Radiative rate constant, *k*_r_ = Φ/τ.

f Nonradiative rate constant, *k*_nr_ = (1 – Φ)/τ.

g Data in the CH_2_Cl_2_ solution from ref (21).

In contrast to complexes **3**, the isomeric **5a** and **5d**, where the carbene moiety is *trans* to the metalated aryl of the C^∧^N ligand, are not emissive in either the CH_2_Cl_2_ solution or PMMA films at room temperature. A similar behavior was previously observed for the homologous unsymmetrical (*OC*-6-32)-[PtCl_2_(C^∧^N)_2_] complexes (Chart 1), which was attributed to a thermally accessible and nonemissive ^3^LMCT excited state that provides an effective nonradiative deactivation pathway.^21^

### Electrochemistry

**Figure 8:**
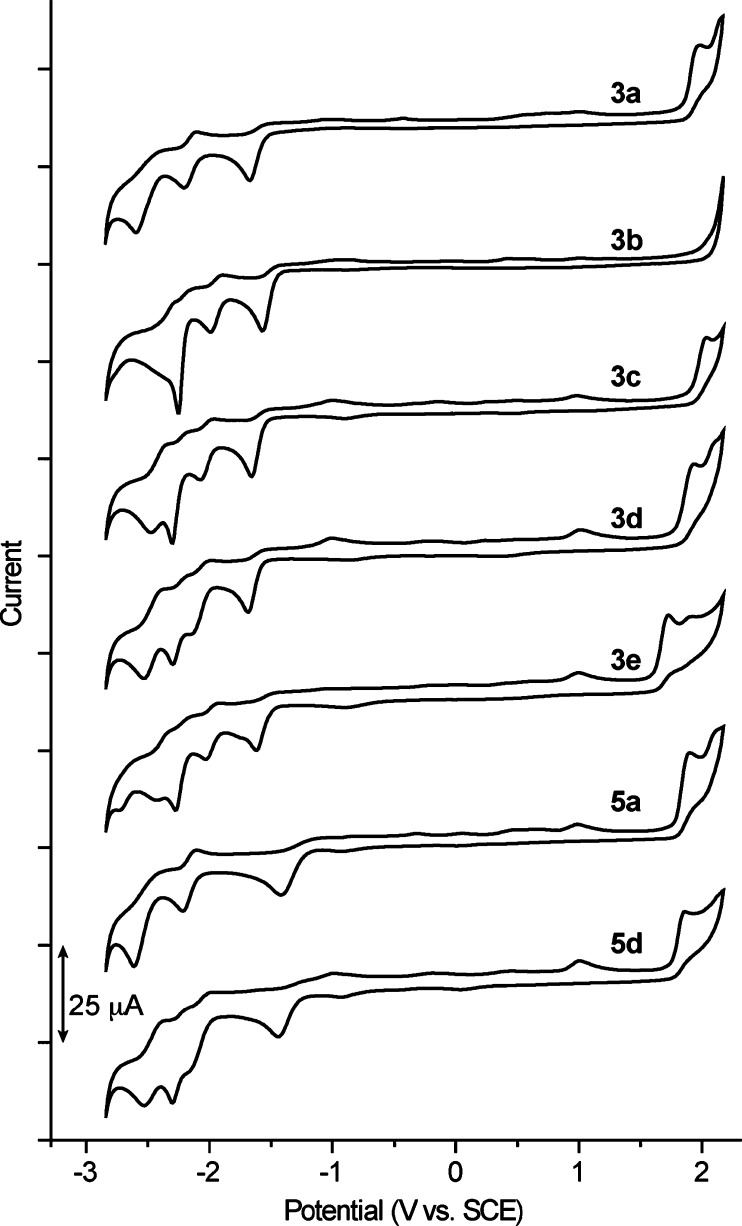
Cyclic voltammograms of complexes **3a**–**e**, **5a**, and **5d** in MeCN at 100 mV s^–1^.

The redox properties of the bis-cyclometalated complexes **3a**–**e**, **5a**, and **5d** were examined by means of cyclic voltammetry in a MeCN solution. The voltammograms are depicted in Figure 8, and the potentials of the most important redox processes and highest occupied and lowest unoccupied molecular orbital (HOMO and LUMO, respectively) energy estimations are compiled in Table 4. An irreversible oxidation peak was observed in the range from 1.71 to 2.03 V vs SCE except for the dfppy derivative **3b**, where the oxidation must fall outside the accessible potential range. The associated HOMO energies vary according to the sequence **3c** < **3a** < **3d** < **3e** and agree with previously determined C^∧^N-based π-orbital energies in cyclometalated Pt(IV) complexes.^19,20,27^ The isomeric pairs **3a/5a** and **3d/5d** possess identical HOMO energies, suggesting that the HOMO is also primarily a π-orbital of the C^∧^N ligand in complexes **5a** and **5d**. The first reduction is irreversible for all complexes and is visible in the range from −1.54 to −1.69 V vs SCE for **3a**–**e**, whereas for **5a** and **5d** it appears at distinctively less negative potentials (−1.41 or −1.43 V vs SCE, respectively). The LUMO energies are almost identical for **3a**, **3c**, and **3d** and somewhat lower for **3b** and **3e**. Since the ppz-, ppy-, and tpy-based LUMOs have been previously shown to have higher energies,^19^ it is likely that the LUMO in derivatives **3a**, **3c**, and **3d** is a π*-orbital of the trz ligand, as predicted by the DFT calculations for **3d** (see below). The LUMO energies found for **3b** and **3e** are compatible with a dfppy- and thpy-based orbital, respectively. In contrast, the significantly lower LUMO energies found for **5a** and **5d** imply that the LUMO is no longer ligand-localized in these complexes. Instead, it is assigned as a dσ*-orbital on the basis of DFT calculations (see below). In all cases, the first reduction is followed by a reversible or quasi-reversible wave in the *E*_1/2_ range from −1.95 to −2.16 V vs SCE, and additional irreversible reductions were also observed. The reversible wave was observed at identical *E*_1/2_ values for each of the pairs **3a/5a** and **3d/5d**, suggesting that the species produced as a consequence of the first irreversible reduction is not dependent on the ligand arrangement; however, its identity cannot be unambiguously established.

**Table 4:** Electrochemical Data^a^ and HOMO and LUMO Energy Estimations^b^ for Complexes **3a**–**e**, **5a**, and **5d**

complex	*E*_p,a_^c^	*E*_p,c_^d^	*E*_1/2_^e^	*E*_HOMO_	*E*_LUMO_	Δ*E*_HOMO–LUMO_
**3a**	1.98	–1.67, –2.59	–2.15	–6.54	–3.15	3.39
**3b**	–f	–1.56, –2.25	–1.95		–3.25	
**3c**	2.03	–1.65, –2.30, –2.48	–2.01	–6.89	–3.15	3.74
**3d**	1.93	–1.69, –2.30, –2.53	–2.06	–6.48	–3.13	3.35
**3e**	1.71, 1.91	–1.54, –2.27, –2.44	–1.97	–6.32	–3.20	3.12
**5a**	1.90	–1.41, –2.60	–2.16	–6.50	–3.48	3.02
**5d**	1.86	–1.43, –2.30, –2.53	–2.06	–6.44	–3.42	3.02

a In volts versus SCE, registered in a 0.1 M solution of (Bu_4_N)PF_6_ in dry MeCN at 100 mV s^–1^.

b In electronvolts.

c Irreversible anodic peak potentials.

d Irreversible cathodic peak potentials.

e For the reversible wave.

f Outside the solvent window.

### Computational Study

**Figure 9:**
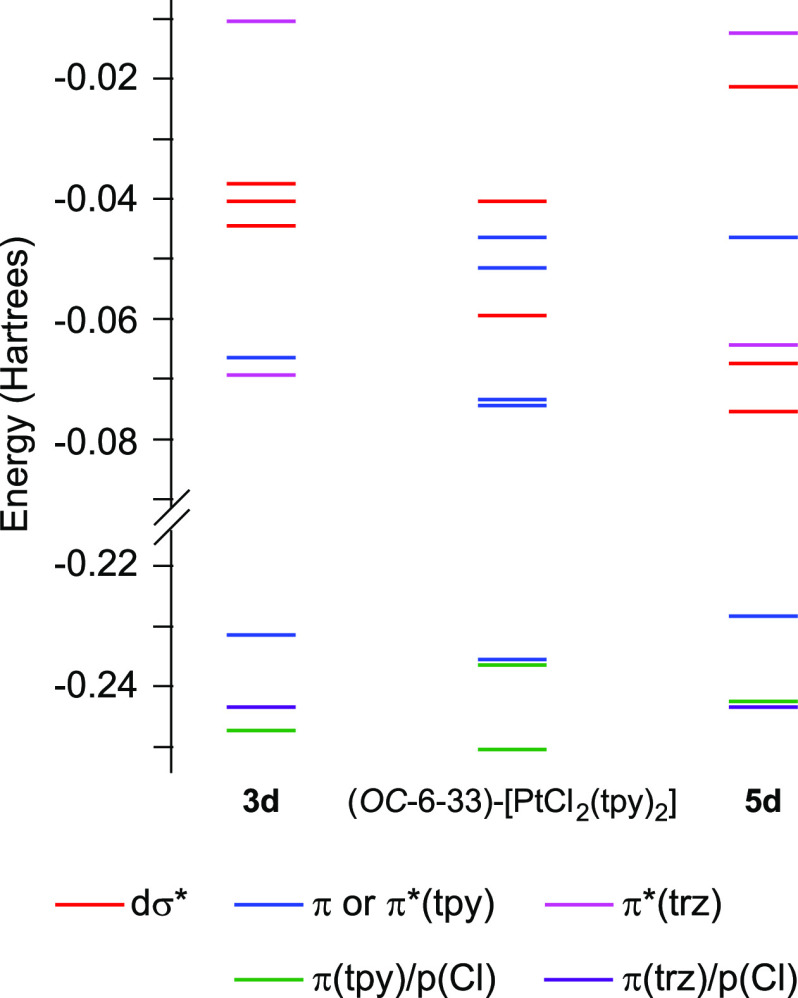
Orbital energy diagrams from DFT calculations for complexes **3d**, (*OC*-6-33)-[PtCl_2_(tpy)_2_], and **5d**.

DFT and TDDFT calculations were performed for complexes **3d**, (*OC*-6-33)-[PtCl_2_(tpy)_2_], and **5d** (see the Supporting Information for details). Frontier orbital energies and their main characters are presented in Figure 9. In the three cases, the HOMO is essentially a π-orbital of the tpy ligand(s), with some contribution from metal dπ*-orbitals (4% for **3d**, 7% for (*OC*-6-33)-[PtCl_2_(tpy)_2_], and 3% for **5d**). The LUMO and LUMO + 1 in **3d** are π-orbitals of the trz and tpy ligands, respectively, and those in (*OC*-6-33)-[PtCl_2_(tpy)_2_] correspond to π*-orbitals delocalized over the two tpy ligands. In contrast, the LUMO in **5d** is essentially a dσ*-orbital that is mostly distributed along the N–Pt–Cl axis and lies at a noticeably lower energy with respect to the trz-based LUMO of **3d**, which agrees with the electrochemical results. Notably, the lowest molecular orbital with a primarily dσ* character in complexes **3d** and (*OC*-6-33)-[PtCl_2_(tpy)_2_] is LUMO + 2, which has a significantly higher energy for the trz complex, implying that a major effect of the carbene is to increase the ligand-field splitting.

The TDDFT calculations reveal a ligand-to-ligand charge transfer (LLCT) from the tpy ligand to the trz ligand, π(tpy)−π*(trz), and a LC transition within the tpy ligand, π(tpy)−π*(tpy), as the lowest singlet excitations in complex **3d** (S_1_ and S_2_, respectively), whereas in (*OC*-6-33)-[PtCl_2_(tpy)_2_] only LC(tpy) excitations are predicted to contribute to the lowest-energy absorptions. The three lowest singlet excitations in complex **5d** are predicted to be weak and involve transitions from π(tpy), π(trz), or p(Cl) orbitals to dσ*-orbitals that can hence be designated as LMCT or LMCT/XMCT; a more intense excitation of primarily LC(tpy) character is predicted at a higher energy (S_4_). Therefore, the presence of low-energy LMCT absorptions explains the red-shifted lowest-energy feature in the absorption spectrum of **5d**.

**Figure 10:**
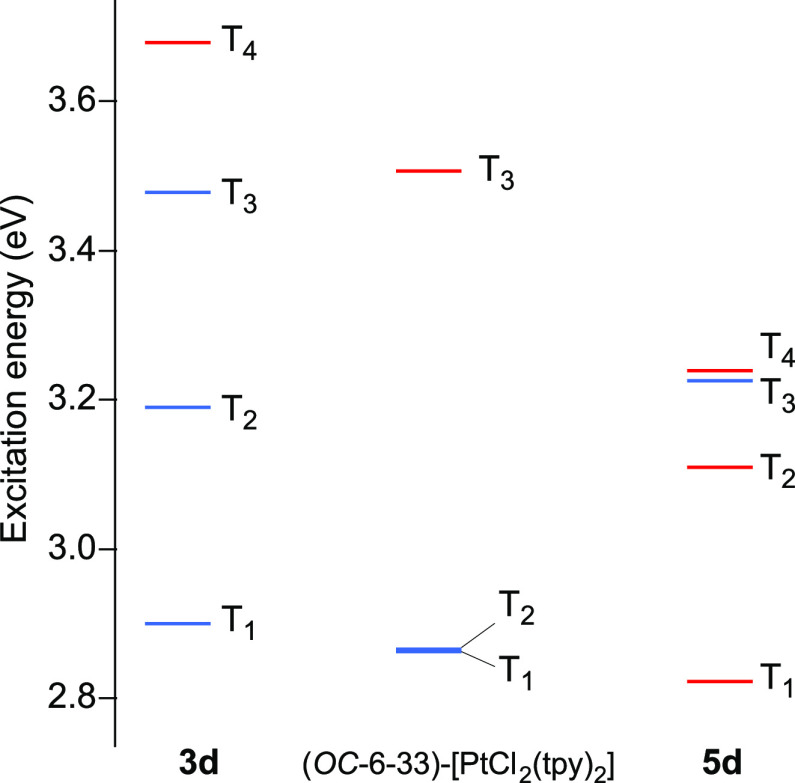
Lowest triplet excitation energies from TDDFT calculations at the ground-state geometry. The red lines correspond to essentially LMCT excitations.

The lowest triplet excitation energies are represented in Figure 10. The first triplet (T_1_) corresponds to an essentially LC(tpy) transition in complexes **3d** and (*OC*-6-33)-[PtCl_2_(tpy)_2_], with a somewhat lower energy for the latter complex that is in agreement with the observed variation in emission energies. The lowest ^3^LMCT excitations are T_4_ in **3d** and T_3_ in (*OC*-6-33)-[PtCl_2_(tpy)_2_]; the energy difference with respect to T_1_ is higher for **3d** (0.78 eV) than for (*OC*-6-33)-[PtCl_2_(tpy)_2_] (0.64 eV), implying that, consistent with the observed lower *k*_*nr*_ values, the thermal population from the emitting state should be less favorable for the carbene complex. In the case of **5d**, the first triplet excitation is a LMCT transition, which explains the lack of emission of this complex.

**Figure 11:**
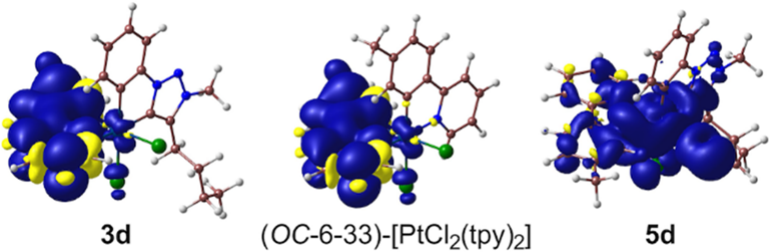
Spin-density distributions (0.001 e bohr^–3^) of the optimized lowest triplet excited states of **3d**, (*OC*-6-33)-[PtCl_2_(tpy)_2_], and **5d**.

For further insight, a geometry optimization of the lowest triplet excited state (T_1_) was carried out for the three studied complexes. The corresponding spin density distribution (Figure 11) matches the topology of a π–π* excitation within the tpy ligand in **3d** or one of the tpy ligands in (*OC*-6-33)-[PtCl_2_(tpy)_2_], which is consistent with an essentially ^3^LC(tpy) emitting state in these complexes, and the associated geometry variations relative to the ground state are mostly limited to the affected ligand (Table S13). The computed adiabatic energy differences with respect to the ground state are 2.78 eV for **3d** and 2.75 eV for (*OC*-6-33)-[PtCl_2_(tpy)_2_], which are a good match with the observed emission energies. The natural spin densities on the Pt atom are 0.0187 for **3d** and 0.0221 for (*OC*-6-33)-[PtCl_2_(tpy)_2_], indicating a small degree of metal orbital contribution and therefore a certain MLCT admixture in the essentially LC emitting state^21^ that is slightly higher for (*OC*-6-33)-[PtCl_2_(tpy)_2_]. This fact is consistent with both the increased metal orbital contribution to the HOMO in the latter complex and its lower emission energy that imply a higher energy of metal dπ orbitals, probably because the arylpyridine is a weaker π-acceptor than the cyclometalated trz.^52^ In the case of **5d**, the spin density distribution in the relaxed T_1_ state clearly corresponds to a LMCT state involving an electronic transition to a dσ* orbital, which causes severe geometry distortions that mostly result from Pt–ligand bond elongations (Table S14).

## Conclusions

Mixed-ligand Pt(IV) derivatives containing cyclometalating trz and C^∧^N ligands of different energies for the lowest π–π* transition have been synthesized by the oxidative addition of PhICl_2_ to Pt(II) precursors of the type *cis*- or trans-C,C*-[PtCl(C^∧^N)(trzH)]. The electrophilic metalation of the pendant phenyl group of the trz ligand upon oxidation proved to be more difficult in comparison to analogous reactions involving 2-arylpyridines and compete with the coordination of a chlorido ligand. The complexes (*OC*-6-54)-[PtCl_2_(C^∧^N)(trz)] that contain cyclometalating 2-arylpyridines (**3b**–**e**) exhibit strong phosphorescent emissions that originate from ^3^LC states primarily localized on the C^∧^N ligand, which can reach quantum yields of ca. 0.3 in a fluid solution and almost unity in PMMA matrices; the latter are the highest efficiencies ever observed for Pt(IV) complexes. Therefore, they constitute a class of strongly emissive compounds whose emission energies can be tuned by varying the C^∧^N ligand. A comparison between the photophysical properties of **3** and those of the homologous *C*_2_-symmetrical complexes (*OC*-6-33)-[PtCl_2_(C^∧^N)_2_] showed that the replacement of one of the C^∧^N ligands for trz results in lower nonradiative decay rates and higher quantum efficiencies. The computational results substantiate a higher energy of dσ* orbitals and deactivating ^3^LMCT states in complexes **3**, which are attributed to the strong σ-donor character of the trz ligand. In contrast, the isomeric complexes (*OC*-6-42)-[PtCl_2_(C^∧^N)(trz)] (**5**), featuring a *trans* arrangement of the carbene and aryl moieties, are not emissive because they present a ^3^LMCT state as the lowest triplet, which involves a low-lying dσ* orbital along the N–Pt–Cl axis.

## Experimental Section

### General Considerations

Unless otherwise noted, procedures were performed at room temperature under atmospheric conditions using synthesis-grade solvents. Reactions involving silver reagents were conducted under a N_2_ atmosphere in the dark. The dichlorido-bridged dimers **1a**(71) and **1b**–**e**,^28^ PhICl_2_,^72^ and the triazolium iodide salt^73^ were synthesized following published procedures. NMR spectra were registered on Bruker Advance 300, 400, or 600 MHz spectrometers. Chemical shifts (δ) are given in parts per million downfield from tetramethylsilane. Elemental analyses were determined using a LECO CHNS-932 microanalyzer. The irradiation of trans-C,C*-**2a** was carried out using a 36 W Philips UVB Narrowband lamp centered at 310 nm. Complexes trans-C,C*-**2b**–**e** were irradiated with Blue LEDs following the previously described experimental setup.^74^

### General Procedure for the Synthesis of trans-C,C*-[PtCl(C^∧^N)(trzH)] (trans-C,C*-**2**)

The triazolium salt (100 mg, 0.29 mmol) and Ag_2_O (37 mg, 0.16 mmol) were suspended in CH_2_Cl_2_ (10 mL), and the mixture was stirred for 14 h. The suspension was filtered through Celite, and [Pt_2_(μ-Cl)_2_(C^∧^N)_2_] (**1**) (0.15 mmol) was immediately added to the filtrate. The mixture was stirred in the dark for 1 h and filtered through Celite. The filtrate was evaporated to dryness, and the residue was washed with Et_2_O (3 × 5 mL) and vacuum-dried to give trans-C,C*-**2**.

#### trans-C,C*-**2a**

White solid, obtained from **1a** (109 mg). Yield: 100 mg, 58%. ^1^H NMR (300 MHz, CD_2_Cl_2_): δ 8.38 (dd, *J*_HH_ = 8.2, 1.7 Hz, 2H, H_arom_), 7.93 (dd, *J*_HH_ = 7.5, 1.8 Hz, 1H, H_arom_), 7.88 (d, *J*_HH_ = 3.0 Hz, 1H, H_arom_), 7.49–7.39 (m, 3H, H_arom_), 7.17–7.09 (m, 3H, H_arom_), 6.89 (d with satellites, *J*_HH_ = 2.3 Hz, *J*_HPt_ = 16 Hz, 1H, H_arom_), 6.26 (br t, *J*_HH_ = 2.6 Hz, 1H, H_arom_), 4.16 (s, 3H, NCH_3_), 3.23 (ddd, *J*_HH_ = 14.6, 9.6, 6.2 Hz, 1H, CH_2_), 2.90 (ddd, *J*_HH_ = 14.6, 9.6, 6.2 Hz, 1H, CH_2_), 1.91–1.69 (m, 2H, CH_2_), 1.45–1.33 (m, 2H, CH_2_), 0.87 (t, *J*_HH_ = 7.3 Hz, 3H, CH_3_). ^13^C NMR (75 MHz, CD_2_Cl_2_): δ 165.6 (C), 149.0 (C), 145.0 (C), 144.6 (C), 140.2 (C), 139.6 (CH), 134.9 (*J*_CPt_ = 34 Hz, CH), 129.8 (CH), 129.4 (CH), 126.2 (*J*_CPt_ = 35 Hz, CH), 125.6 (*J*_CPt_ = 38 Hz, CH), 124.7 (CH), 124.0 (CH), 110.5 (*J*_CPt_ = 16 Hz, CH), 107.4 (*J*_CPt_ = 40 Hz, CH), 36.8 (NCH_3_), 31.6 (CH_2_), 25.4 (CH_2_), 23.0 (CH_2_), 14.1 (CH_3_). Anal. Calcd for C_22_H_24_ClN_5_Pt: C, 44.79; H, 4.27; N, 11.87. Found: C, 44.81; H, 4.19; N, 11.80.

#### trans-C,C*-**2b**

Yellow solid, obtained from **1b** (116 mg). Yield: 141 mg, 62%. ^1^H NMR (600 MHz, CD_2_Cl_2_): δ 8.38–8.35 (m, 2H, H_arom_), 8.05–7.96 (m, 2H, H_arom_), 7.78 (br t, *J*_HH_ = 8.0 Hz, 1H, H_arom_), 7.65 (dd, *J*_HH_ = 8.8, 2.5 Hz, 1H, H_arom_), 7.48–7.38 (m, 3H, H_arom_), 6.72 (ddd, *J*_HH_ = 7.4, 6.0, 1.5 Hz, 1H, H_arom_), 6.56 (ddd, *J*_HH_ = 12.7, 8.9, 2.5 Hz, 1H, H_arom_), 4.19 (s, 3H, NCH_3_), 3.22 (ddd, *J*_HH_ = 14.7, 9.6, 6.1 Hz, 1H, CH_2_), 2.88 (ddd, *J*_HH_ = 14.7, 9.6, 6.1 Hz, 1H, CH_2_), 1.91–1.81 (m, 1H, CH_2_), 1.80–1.70 (m, 1H, CH_2_), 1.43–1.33 (m, 2H, CH_2_), 0.85 (t, *J*_HH_ = 7.3 Hz, 3H, CH_3_). ^13^C APT NMR (151 MHz, CD_2_Cl_2_): δ 169.2 (C), 166.9 (d, *J*_CF_ = 7 Hz, C), 165.6 (C), 164.2 (dd, *J*_CF_ = 256, 11 Hz, C), 161.2 (dd, *J*_CF_ = 260, 12 Hz, C), 151.6 (CH), 148.7 (C), 140.2 (C), 138.3 (CH), 130.1 (CH), 129.5 (CH), 124.7 (CH), 123.2 (d, *J*_CF_ = 20 Hz, CH), 122.9 (CH), 115.7 (d, *J*_CF_ = 17 Hz, CH), 99.4 (t, *J*_CF_ = 27 Hz, CH), 37.0 (NCH_3_), 31.5 (CH_2_), 25.6 (CH_2_), 23.1 (CH_2_), 14.1 (CH_3_). ^19^F NMR (282 MHz, CD_2_Cl_2_): δ −108.16 (m, 1H), −112.45 (m, 1H). Anal. Calcd for C_24_H_23_ClF_2_N_4_Pt: C, 45.32; H, 3.65; N, 8.81. Found: C, 45.14; H, 3.56; N, 8.74.

#### trans-C,C*-**2c**

Yellow solid, obtained from **1c** (112 mg). Yield: 135 mg, 78%. ^1^H NMR (600 MHz, CD_2_Cl_2_): δ 8.46–8.43 (m, 2H, H_arom_), 8.02 (dd, *J*_HH_ = 7.5, 1.2 Hz, 1H, H_arom_), 7.96 (ddd with satellites, *J*_HH_ = 5.9, 1.6, 0.8 Hz, *J*_HPt_ = 48 Hz, 1H, H_arom_), 7.74 (ddd, *J*_HH_ = 8.7, 7.4, 1.5 Hz, 1H, H_arom_), 7.64 (d, *J*_HH_ = 8.1 Hz, 1H, H_arom_), 7.49 (d, *J*_HH_ = 7.7 Hz, 1H, H_arom_), 7.46–7.42 (m, 2H, H_arom_), 7.42–7.38 (m, 1H, H_arom_), 7.25 (td, *J*_HH_ = 7.3, 1.2 Hz, 1H, H_arom_), 7.09 (td, *J*_HH_ = 7.5, 1.3 Hz, 1H, H_arom_), 6.68 (ddd, *J*_HH_ = 7.4, 5.9, 1.5 Hz, 1H, H_arom_), 4.18 (s, 3H, NCH_3_), 3.25 (ddd, *J*_HH_ = 14.7, 9.6, 6.0 Hz, 1H, CH_2_), 2.89 (ddd, *J*_HH_ = 14.7, 9.5, 6.1 Hz, 1H, CH_2_), 192–1.83 (m, 1H, CH_2_), 1.80–1.72 (m, 1H, CH_2_), 1.43–1.33 (m, 2H, CH_2_), 0.85 (t, *J*_HH_ = 7.3 Hz, 3H, CH_3_). ^13^C APT NMR (151 MHz, CD_2_Cl_2_): δ 171.4 (C), 170.3 (C), 160.1 (C), 151.3 (CH), 148.7 (C), 146.3 (C), 140.3 (C), 137.6 (CH), 134.1 (CH), 130.1 (CH), 129.9 (CH), 129.4 (CH), 124.5 (CH), 123.7 (CH), 123.3 (CH), 122.7 (CH), 119.5 (*J*_CPt_ = 43 Hz, CH), 36.9 (NCH_3_), 31.5 (CH_2_), 25.5 (CH_2_), 23.0 (CH_2_), 14.1 (CH_3_). Anal. Calcd for C_24_H_25_ClN_4_Pt: C, 48.04; H, 4.20; N, 9.34. Found: C, 48.02; H, 4.16; N, 9.47.

#### trans-C,C*-**2d**

Yellow solid, obtained from **1d** (116 mg). Yield: 127 mg, 59%. ^1^H NMR (300 MHz, CD_2_Cl_2_): δ 8.47–8.42 (m, 2H, H_arom_), 7.92 (ddd with satellites, *J*_HH_ = 5.9, 1.5, 0.7 Hz, *J*_HPt_ = 53 Hz, 1H, H_arom_), 7.83 (br s with satellites, *J*_HPt_ = 26 Hz, 1H, H_arom_), 7.70 (ddd, *J*_HH_ = 8.2, 7.3, 1.6 Hz, 1H, H_arom_), 7.58 (ddd, *J*_HH_ = 8.3, 1.6, 0.7 Hz, 1H, H_arom_), 7.47–7.35 (m, 4H, H_arom_), 6.91 (ddd, *J*_HH_ = 7.9, 1.8, 0.7 Hz, 1H, H_arom_), 6.63 (ddd, *J*_HH_ = 7.4, 5.9, 1.6 Hz, 1H, H_arom_), 4.17 (s, 3H, NCH_3_), 3.25 (ddd, *J*_HH_ = 14.7, 9.5, 6.1 Hz, 1H, CH_2_), 2.88 (ddd, *J*_HH_ = 14.6, 9.4, 6.2 Hz, 1H, CH_2_), 2.36 (s, 3H, CH_3_), 1.93–1.66 (m, 2H, CH_2_), 1.44–1.27 (m, 2H, CH_2_), 0.84 (t, *J*_HH_ = 7.3 Hz, 3H, CH_3_). ^13^C APT NMR (75 MHz, CD_2_Cl_2_): δ 171.5 (C), 170.3 (C), 159.8 (C), 151.2 (*J*_CPt_ = 18 Hz, CH), 148.6 (C), 143.6 (C), 140.2 (C), 137.5 (CH), 134.8 (*J*_CPt_ = 50 Hz, CH), 129.8 (CH), 129.4 (CH), 124.6 (CH), 124.4 (CH), 123.3 (*J*_CPt_ = 28 Hz, CH), 122.2 (*J*_CPt_ = 46 Hz, CH), 119.2 (*J*_CPt_ = 45 Hz, CH), 36.8 (NCH_3_), 31.4 (CH_2_), 25.5 (CH_2_), 23.0 (CH_2_), 22.3 (CH_3_), 14.1 (CH_3_). Anal. Calcd for C_25_H_27_ClN_4_Pt: C, 48.90; H, 4.43; N, 9.12. Found: C, 48.86; H, 4.52; N, 8.83.

#### trans-C,C*-**2e**

Orange solid, obtained from **1e** (117 mg). Yield: 141 mg, 80%. ^1^H NMR (600 MHz, CD_2_Cl_2_): δ 8.42–8.40 (m, 2H, H_arom_), 7.72 (ddd with satellites, *J*_HH_ = 5.9, 1.5, 0.8 Hz, *J*_HPt_ = 50 Hz, 1H, H_arom_), 7.61 (ddd, *J*_HH_ = 8.1, 7.4, 1.5 Hz, 1H, H_arom_), 7.48–7.40 (m, 4H, H_arom_), 7.35 (d, *J*_HH_ = 4.6 Hz, 1H, H_arom_), 7.27 (ddd, *J*_HH_ = 8.0, 1.5, 0.8 Hz, 1H, H_arom_), 6.51 (ddd, *J*_HH_ = 7.4, 5.9, 1.5 Hz, 1H, H_arom_), 4.18 (s, 3H, NCH_3_), 3.24 (ddd, *J*_HH_ = 14.7, 9.6, 6.0 Hz, 1H, CH_2_), 2.90 (ddd, *J*_HH_ = 14.7, 9.6, 6.1 Hz, 1H, CH_2_), 1.91–1.83 (m, 1H, CH_2_), 1.80–1.72 (m, 1H, CH_2_), 1.42–1.35 (m, 2H, CH_2_), 0.86 (t, *J*_HH_ = 7.3 Hz, 3H, CH_3_). ^13^C APT NMR (151 MHz, CD_2_Cl_2_): δ 168.5 (C), 168.0 (C), 165.2 (C), 151.3 (CH), 148.6 (C), 141.6 (C), 140.2 (C), 138.5 (CH), 133.9 (*J*_CPt_ = 78 Hz, CH), 130.0 (CH), 129.4 (CH), 128.0 (CH), 124.6 (CH), 120.3 (CH), 118.2 (CH), 36.9 (NCH_3_), 31.5 (CH_2_), 25.5 (CH_2_), 23.0 (CH_2_), 14.1 (CH_3_). Anal. Calcd for C_22_H_23_ClN_4_PtS: C, 43.60; H, 3.83; N, 9.24; S, 5.29. Found: C, 43.50; H, 3.87; N, 9.28; S, 5.09.

### General Procedure for the Synthesis of cis-C,C*-[PtCl(C^∧^N)(trzH)] (cis-C,C*-**2**)

A deaerated solution of trans-C,C*-**2** in acetone (20 mL) was irradiated with blue LEDs for 16–72 h under a N_2_ atmosphere. The solvent was evaporated to dryness, and the residue was washed with Et_2_O (3 × 3 mL) and vacuum-dried to give cis-C,C*-**2**. For the ppz-derivative **2a**, MeCN (5 mL) and UV light were used.

#### cis-C,C*-**2a**

White solid, obtained from trans-C,C*-**2a** (50 mg) after 16 h of UV irradiation. Yield: 34 mg, 68%. ^1^H NMR (400 MHz, CD_2_Cl_2_): δ 8.35–8.28 (m, 2H, H_arom_), 8.06 (d, *J*_HH_ = 2.2 Hz, 1H, H_arom_), 7.94 (d, *J*_HH_ = 2.6 Hz, 1H, H_arom_), 7.46–7.39 (m, 3H, H_arom_), 7.11 (d, *J*_HH_ = 7.6 Hz, 1H, H_arom_), 6.98 (t, *J*_HH_ = 7.4 Hz, 1H, H_arom_), 6.69 (t, *J*_HH_ = 7.3 Hz, 1H, H_arom_), 6.55 (t, *J*_HH_ = 2.5 Hz, 1H, H_arom_), 6.46 (d, *J*_HH_ = 7.6 Hz, 1H, H_arom_), 4.18 (s, 3H, NCH_3_), 3.11 (ddd, *J*_HH_ = 15.0, 9.6, 5.8 Hz, 1H, CH_2_), 2.89 (ddd, *J*_HH_ = 15.0, 9.6, 6.3 Hz, 1H, CH_2_), 1.91–1.67 (m, 2H, CH_2_), 1.43–1.33 (m, 2H, CH_2_), 0.86 (t, *J*_HH_ = 7.3 Hz, 3H, CH_3_). ^13^C APT NMR (100 MHz, CD_2_Cl_2_): δ 146.6 (C), 146.5 (C), 144.6 (C), 140.4 (C), 139.0 (*J*_CPt_ = 48 Hz, CH), 135.8 (*J*_CPt_ = 86 Hz, CH), 129.9 (CH), 129.2 (CH), 128.1 (C), 126.3 (CH), 126.0 (CH), 125.4 (CH), 123.2 (CH), 111.6 (*J*_CPt_ = 28 Hz, CH), 107.1 (CH), 37.1 (NCH_3_), 31.1 (CH_2_), 25.8 (*J*_CPt_ = 23 Hz, CH_2_), 23.1 (CH_2_), 14.1 (CH_3_). Anal. Calcd for C_22_H_24_ClN_5_Pt: C, 44.79; H, 4.27; N, 11.87. Found: C, 44.77; H, 4.25; N, 11.69.

#### cis-C,C*-**2b**

Yellow solid, obtained from trans-C,C*-**2b** (54 mg) after 72 h of irradiation. Yield: 25 mg, 47%. ^1^H NMR (600 MHz, CD_2_Cl_2_): δ 9.60 (br d, *J* = 5.8 Hz, 1H, H_arom_), 8.29–8.24 (m, 2H, H_arom_), 8.05 (d, *J* = 8.2 Hz, 1H, H_arom_), 7.87 (td, *J* = 8.1, 1.3 Hz, 1H, H_arom_), 7.46–7.41 (m, 3H, H_arom_), 7.28 (ddd, *J* = 7.3, 5.6, 1.4 Hz, 1H, H_arom_), 6.48 (ddd, *J* = 12.7, 9.2, 2.4 Hz, 1H, H_arom_), 6.04 (dd with satellites, *J* = 9.1, 2.4 Hz, *J*_HPt_ = 78 Hz, 1H, H_arom_), 4.19 (s, 3H, NCH_3_), 3.09 (ddd, *J*_HH_ = 15.0, 9.7, 5.8 Hz, 1H, CH_2_), 2.87 (ddd, *J*_HH_ = 14.7, 9.7, 6.3 Hz, 1H, CH_2_), 1.91–1.82 (m, 1H, CH_2_), 1.79–1.70 (m, 1H, CH_2_), 1.43–1.33 (m, 2H, CH_2_), 0.87 (t, *J*_HH_ = 7.3 Hz, 3H, CH_3_). ^13^C APT NMR (151 MHz, CD_2_Cl_2_): δ 164.1 (dd, *J*_CF_ = 254, 13 Hz, C), 162.9 (d, *J*_CF_ = 7 Hz, C), 161.0 (dd, *J*_CF_ = 258, 13 Hz, C), 149.4 (CH), 147.5 (d with satellites, *J*_CF_ = 7 Hz, *J*_CPt_ ∼ 1160 Hz, C), 147.1 (C), 146.5 (C), 140.3 (C), 139.4 (CH), 130.0 (CH), 129.3 (CH), 129.1 (C), 125.3 (CH), 122.6 (CH), 122.5 (d, *J*_CF_ = 18 Hz, CH), 117.1 (d with satellites, *J*_CF_ = 18 Hz, *J*_CPt_ = 101 Hz, CH), 98.7 (t, *J*_CF_ = 27 Hz, CH), 37.2 (NCH_3_), 31.0 (CH_2_), 25.7 (CH_2_), 23.1 (CH_2_), 14.0 (CH_3_). ^19^F NMR (282 MHz, CD_2_Cl_2_): δ −109.98 (m, 1H), −110.97 (m, 1H). Anal. Calcd for C_24_H_23_ClF_2_N_4_Pt: C, 45.32; H, 3.65; N, 8.81. Found: C, 45.51; H, 3.75; N, 9.05.

#### cis-C,C*-**2c**

Yellow solid, obtained from trans-C,C*-**2c** (60 mg) after 16 h of irradiation. Yield: 45 mg, 75%. ^1^H NMR (600 MHz, CD_2_Cl_2_): δ 9.54 (ddd, *J*_HH_ = 5.6, 1.7, 0.8 Hz, 1H, H_arom_), 8.35–8.31 (m, 2H, H_arom_), 7.85–7.82 (m, 1H, H_arom_), 7.69 (d, *J*_HH_ = 8.1 Hz, 1H, H_arom_), 7.46 (dd, *J*_HH_ = 7.8, 1.2 Hz, 1H, H_arom_), 7.44–7.38 (m, 3H, H_arom_), 7.25 (ddd, *J*_HH_ = 7.1, 5.7, 1.3 Hz, 1H, H_arom_), 6.97 (td, *J*_HH_ = 7.5, 1.2 Hz, 1H, H_arom_), 6.80 (td, *J*_HH_ = 7.4, 1.4 Hz, 1H, H_arom_), 6.48 (dd, with satellites, *J*_HH_ = 7.6, 1.2 Hz, *J*_HPt_ = 67 Hz, 1H, H_arom_), 4.18 (s, 3H, NCH_3_), 3.09 (ddd, *J*_HH_ = 14.6, 9.7, 5.8 Hz, 1H, CH_2_), 3.33 (ddd, *J*_HH_ = 14.7, 9.7, 6.3 Hz, 1H, CH_2_), 1.90–1.83 (m, 1H, CH_2_), 1.78–1.71 (m, 1H, CH_2_), 1.42–1.33 (m, 2H, CH_2_), 0.86 (t, *J*_HH_ = 7.3 Hz, 3H, CH_3_). ^13^C APT NMR (151 MHz, CD_2_Cl_2_): δ 166.1 (C), 149.2 (CH), 148.2 (C), 146.4 (C), 145.4 (C), 143.3 (*J*_CPt_ ∼ 1138 Hz, C), 140.5 (C), 138.9 (CH), 135.2 (*J*_CPt_ = 105 Hz, CH), 130.5 (*J*_CPt_ = 80 Hz, CH), 129.8 (CH), 129.2 (CH), 125.3 (CH), 124.0 (*J*_CPt_ = 42 Hz, CH), 122.8 (CH), 122.6 (CH), 118.6 (CH), 37.1 (NCH_3_), 31.0 (CH_2_), 25.8 (CH_2_), 23.1 (CH_2_), 14.1 (CH_3_). Anal. Calcd for C_24_H_25_ClN_4_Pt: C, 48.04; H, 4.20; N, 9.34. Found: C, 48.07; H, 4.34; N, 9.41.

#### cis-C,C*-**2d**

Yellow solid, obtained from trans-C,C*-**2d** (70 mg) after 16 h of irradiation. Yield: 52 mg, 75%. ^1^H NMR (300 MHz, CD_2_Cl_2_): δ 9.50 (ddd with satellites, *J*_HH_ = 5.7, 1.7, 0.8 Hz, *J*_HPt_ = 21 Hz, 1H, H_arom_), 8.35–8.28 (m, 2H, H_arom_), 7.79 (ddd, *J*_HH_ = 8.2, 7.4, 1.7 Hz, 1H, H_arom_), 7.63 (dt, *J*_HH_ = 8.1, 1.2 Hz, 1H, H_arom_), 7.47–7.38 (m, 3H, H_arom_), 7.35 (d with satellites, *J*_HH_ = 7.9 Hz, *J*_HPt_ ∼ 7 Hz, 1H, H_arom_), 7.20 (ddd, *J*_HH_ = 7.2, 5.7, 1.4 Hz, 1H, H_arom_), 6.79 (ddd, *J*_HH_ = 7.9, 1.8, 0.7 Hz, 1H, H_arom_), 6.31 (s with satellites, *J*_HPt_ = 70 Hz, 1H, H_arom_), 4.18 (s, 3H, NCH_3_), 3.13 (ddd, *J*_HH_ = 14.9, 9.4, 5.7 Hz, 1H, CH_2_), 2.88 (ddd, *J*_HH_ = 14.5, 9.4, 6.6 Hz, 1H, CH_2_), 2.12 (s, 3H, CH_3_), 1.97–1.65 (m, 2H, CH_2_), 1.44–1.27 (m, 2H, CH_2_), 0.85 (t, *J*_HH_ = 7.3 Hz, 3H, CH_3_). ^13^C APT NMR (75 MHz, CD_2_Cl_2_): δ 166.1 (C), 149.0 (*J*_CPt_ = 20 Hz, CH), 148.3 (C), 146.4 (C), 143.1 (C), 142.7 (C), 140.5 (C), 138.8 (CH), 136.0 (*J*_CPt_ = 107 Hz, CH), 129.7 (CH), 129.1 (CH), 125.3 (CH), 123.9 (*J*_CPt_ = 45 Hz, CH), 123.7 (CH), 122.1 (*J*_CPt_ = 20 Hz, CH), 118.2 (*J*_CPt_ = 29 Hz, CH), 37.2 (NCH_3_), 30.9 (CH_2_), 25.8 (*J*_CPt_ = 24 Hz, CH_2_), 23.0 (CH_2_), 21.8 (CH_3_), 14.0 (CH_3_). Anal. Calcd for C_25_H_27_ClN_4_Pt: C, 48.90; H, 4.43; N, 9.12. Found: C, 48.70; H, 4.41; N, 8.86.

#### cis-C,C*-**2e**

Orange solid, obtained from trans-C,C*-**2e** (70 mg) after 40 h of irradiation. Yield: 68 mg, 98%. ^1^H NMR (600 MHz, CD_2_Cl_2_): δ 9.28 (d, *J*_HH_ = 5.8 Hz, 1H, H_arom_), 8.31–8.27 (m, 2H, H_arom_), 7.71 (td, *J*_HH_ = 7.7, 1.6 Hz, 1H, H_arom_), 7.45–7.41 (m, 3H, H_arom_), 7.31 (d, *J*_HH_ = 7.8 Hz, 1H, H_arom_), 7.14 (br d, *J*_HH_ = 4.7 Hz, 1H, H_arom_), 7.09 (br t, *J*_HH_ = 6.6 Hz, 1H, H_arom_), 6.15 (d, *J*_HH_ = 4.8 Hz, 1H, H_arom_), 4.16 (s, 3H, NCH_3_), 3.09 (ddd, *J*_HH_ = 15.0, 9.6, 5.8 Hz, 1H, CH_2_), 2.87 (ddd, *J*_HH_ = 15.0, 9.5, 6.2 Hz, 1H, CH_2_), 1.88–1.79 (m, 1H, CH_2_), 1.76–1.67 (m, 1H, CH_2_), 1.42–1.33 (m, 2H, CH_2_), 0.87 (t, *J*_HH_ = 7.3 Hz, 3H, CH_3_). ^13^C APT NMR (151 MHz, CD_2_Cl_2_): δ 161.8 (C), 149.1 (CH), 146.8 (*J*_CPt_ = 90 Hz, C), 145.8 (*J*_CPt_ = 1150 Hz, C), 143.3 (C), 140.4 (C), 139.8 (C), 139.4 (CH), 133.3 (*J*_CPt_ = 150 Hz, CH), 129.9 (CH), 129.2 (CH), 128.3 (*J*_CPt_ = 94 Hz, CH), 125.2 (CH), 120.1 (CH), 117.2 (CH), 37.2 (NCH_3_), 31.1 (CH_2_), 25.8 (CH_2_), 23.0 (CH_2_), 14.1 (CH_3_). Anal. Calcd for C_22_H_23_ClN_4_PtS: C, 43.60; H, 3.83; N, 9.24; S, 5.29. Found: C, 43.73; H, 3.98; N, 9.19; S, 5.19.

### General Procedure for the Synthesis of (*OC*-6-54)-[PtCl_2_(C^∧^N)(trz)] (**3**)

To a solution of cis-C,C*-2 in CH_2_Cl_2_ (5 mL) was added PhICl_2_, and the mixture was stirred for 30 min. Partial evaporation under reduced pressure (2 mL) and the addition of Et_2_O (20 mL) led to the precipitation of a pale-yellow solid, which was filtered off and vacuum dried. The obtained product was placed in a Carius tube with 1,2-dichlorobenzene (1 mL) and Na_2_CO_3_ (30 mg), and the suspension was heated at 130 °C for 16 h under a N_2_ atmosphere. After cooling to room temperature, Et_2_O (10 mL) was added, and the resulting suspension was filtered. The collected solid was extracted with CH_2_Cl_2_ (5 × 5 mL). The partial evaporation of the resulting solution under reduced pressure (2 mL) and the addition of Et_2_O (10 mL) led to the precipitation of a solid, which was filtered off and vacuum dried to give **3**.

#### (*OC*-6-54)-[PtCl_2_(ppz)(trz)] (**3a**)

White solid, obtained from cis-C,C*-**2a** (55 mg, 0.093 mmol) and PhICl_2_ (31 mg, 0.112 mmol). Yield: 28 mg, 48%. ^1^H NMR (600 MHz, CD_2_Cl_2_): δ 8.44 (d, *J*_HH_ = 2.4 Hz, 1H, H_arom_), 8.26 (d, *J*_HH_ = 2.8 Hz, 1H, H_arom_), 7.61 (d, *J*_HH_ = 7.8 Hz, 1H, H_arom_), 7.34 (d, *J*_HH_ = 8.0 Hz, 1H, H_arom_), 7.14 (t, *J*_HH_ = 7.6 Hz, 1H, H_arom_), 7.11 (t, *J*_HH_ = 7.7 Hz, 1H, H_arom_), 6.97–6.93 (m, 1H, H_arom_), 6.87 (s, 1H, H_arom_), 6.84–6.79 (m, 1H, H_arom_), 6.41 (d with satellites, *J*_HH_ = 7.8 Hz, *J*_HPt_ = 49 Hz, 1H, H_arom_), 6.21 (d with satellites, *J*_HH_ = 7.9 Hz, *J*_HPt_ = 43 Hz, 1H, H_arom_), 4.27 (s, 3H, NCH_3_), 3.51–3.43 (m, 1H, CH_2_), 3.36–3.30 (m, 1H, CH_2_), 1.86–1.74 (m, 2H, CH_2_), 1.57–1.47 (m, 2H, CH_2_), 0.97 (t, *J*_HH_ = 7.3 Hz, 3H, CH_3_). ^13^C APT NMR (151 MHz, CD_2_Cl_2_): δ 145.4 (*J*_CPt_ = 85 Hz, C), 140.8 (C), 139.6 (C), 138.6 (*J*_CPt_ = 22 Hz, CH), 132.2 (*J*_CPt_ = 41 Hz, CH), 130.7 (CH), 130.4 (*J*_CPt_ = 44 Hz, CH), 128.4 (*J*_CPt_ = 797 Hz, C), 127.9 (CH), 127.7 (CH), 126.4 (2CH), 124.9 (C), 116.0 (*J*_CPt_ = 31 Hz, CH), 113.7 (*J*_CPt_ = 21 Hz, CH), 109.2 (CH), 37.5 (NCH_3_), 31.8 (CH_2_), 24.1 (CH_2_), 23.1 (CH_2_), 14.1 (CH_3_). Anal. Calcd for C_22_H_23_Cl_2_N_5_Pt: C, 42.38; H, 3.72; N, 11.23. Found: C, 42.23; H, 3.82; N, 10.94.

#### (*OC*-6-54)-[PtCl_2_(dfppy)(trz)] (**3b**)

In this case, a different synthetic procedure from the general method was followed. To a solution of cis-C,C*-**2b** (60 mg, 0.078 mmol) in CH_2_Cl_2_ (5 mL) was added PhICl_2_ (26 mg, 0.094 mmol), and the mixture was stirred for 30 min. Partial evaporation under reduced pressure (2 mL) and the addition of Et_2_O (20 mL) led to the precipitation of a white solid, which was filtered off, washed with MeOH (2 × 1 mL) and Et_2_O (2 × 3 mL), and vacuum dried to give **3b**. Yield: 26 mg, 50%. ^1^H NMR (600 MHz, CD_2_Cl_2_): δ 9.84 (ddd with satellites, *J* = 5.7, 1.7, 0.8 Hz, *J*_PtH_ = 15 Hz, 1H, H_arom_), 8.39 (d, *J* = 8.2 Hz, 1H, H_arom_), 8.14 (td, *J* = 8.0, 1.7 Hz, 1H, H_arom_), 7.63 (dd with satellites, *J* = 7.9, 1.3 Hz, *J*_PtH_ = 10 Hz, 1H, H_arom_), 7.60 (ddd, *J* = 7.3, 5.7, 1.4 Hz, 1H, H_arom_), 7.15 (td, *J* = 7.7, 1.2 Hz, 1H, H_arom_), 6.92 (td with satellites, *J* = 7.7, 1.4 Hz, *J*_PtH_ ∼ 8 Hz, 1H, H_arom_), 6.64 (ddd, *J* = 12.3, 8.9, 2.4 Hz, 1H, H_arom_), 6.15 (dd with satellites, *J* = 7.9, 1.2 Hz, *J*_PtH_ = 43 Hz, 1H, H_arom_), 6.04 (ddd with satellites, *J* = 8.1, 2.4, 0.9 Hz, *J*_PtH_ = 54 Hz, 1H, H_arom_), 4.29 (s, 3H, NCH_3_), 3.44–3.34 (m, 2H, CH_2_), 1.90–1.82 (m, 1H, CH_2_), 1.82–1.73 (m, 1H, CH_2_), 1.55–1.48 (m, 2H, CH_2_), 0.98 (t, *J*_HH_ = 7.4 Hz, 3H, CH_3_). ^13^C APT NMR (151 MHz, CD_2_Cl_2_): δ 163.2 (dd, *J*_CF_ = 360, 12 Hz, C), 161.5 (dd, *J*_CF_ = 364, 13 Hz, C), 159.9 (d, *J*_CF_ = 7 Hz, C), 148.8 (CH), 145.6 (*J*_CPt_ = 78 Hz, C), 142.2 (d, *J*_CF_ = 8 Hz, C), 141.1 (CH), 141.0 (*J*_CPt_ = 12 Hz, C), 131.7 (C), 130.3 (*J*_CPt_ = 45 Hz, CH), 129.9 (CH), 128.2 (*J*_CPt_ = 798 Hz, C), 126.6 (CH), 124.8–124.5 (m, 2CH), 116.2 (*J*_CPt_ = 32 Hz, CH), 114.6 (d, *J*_CPt_ ∼ 52 Hz, *J*_CF_ = 23 Hz, CH), 102.0 (t, *J*_CF_ = 27 Hz, CH), 37.6 (NCH_3_), 31.8 (CH_2_), 24.1 (CH_2_), 23.1 (CH_2_), 14.1 (CH_3_). ^19^F NMR (282 MHz, CD_2_Cl_2_): δ −105.90 (m, 1H), −107.80 (m, 1H). Anal. Calcd for C_24_H_22_Cl_2_F_2_N_4_Pt: C, 43.00; H, 3.31; N, 8.36. Found: C, 43.06; H, 3.28; N, 8.31.

#### (*OC*-6-54)-[PtCl_2_(ppy)(trz)] (**3c**)

White solid, obtained from cis-C,C*-**2c** (60 mg, 0.100 mmol) and PhICl_2_ (33 mg, 0.120 mmol). Yield: 32 mg, 48%. ^1^H NMR (600 MHz, CD_2_Cl_2_): δ 9.80 (d with satellites, *J*_HH_ = 6.5 Hz, *J*_HPt_ = 14 Hz, 1H, H_arom_), 8.11 (td, *J*_HH_ = 7.8, 1.6 Hz, 1H, H_arom_), 8.05 (d, *J*_HH_ = 8.1 Hz, 1H, H_arom_), 7.70 (dd, *J*_HH_ = 7.8, 1.6 Hz, 1H, H_arom_), 7.61 (dd with satellites, *J*_HH_ = 7.9, 1.4 Hz, *J*_HPt_ = 9 Hz, 1H, H_arom_), 7.57 (ddd, *J*_HH_ = 7.3, 5.7, 1.5 Hz, 1H, H_arom_), 7.14–7.09 (m, 2H, H_arom_), 6.92 (td with satellites, *J*_HH_ = 7.6, 1.4 Hz,, *J*_HPt_ ∼ 8 Hz, 1H, H_arom_), 6.89 (td with satellites, *J*_HH_ = 7.6, 1.3 Hz, *J*_HPt_ ∼ 8 Hz, 1H, H_arom_), 6.43 (dd with satellites, *J*_HH_ = 7.8, 0.6 Hz, *J*_HPt_ = 46 Hz, 1H, H_arom_), 6.16 (dd with satellites, *J*_HH_ = 7.9, 1.2 Hz, *J*_HPt_ = 44 Hz, 1H, H_arom_), 4.27 (s, 3H, NCH_3_), 3.46 (ddd, *J*_HH_ = 14.3, 10.4, 5.6 Hz, 1H, CH_2_), 3.33 (ddd, *J*_HH_ = 14.3, 10.4, 5.8 Hz, 1H, CH_2_), 1.88–1.74 (m, 2H, CH_2_), 1.51 (h, *J*_HH_ = 7.4 Hz, 2H, CH_2_), 0.97 (t, *J*_HH_ = 7.4 Hz, 3H, CH_3_). ^13^C APT NMR (151 MHz, CD_2_Cl_2_): δ 162.8 (*J*_CPt_ = 52 Hz, C), 148.6 (CH), 145.5 (*J*_CPt_ = 82 Hz, C), 141.3 (C), 141.1 (C), 140.6 (CH), 139.9 (*J*_CPt_ = 792 Hz, C), 132.5 (*J*_CPt_ = 1112 Hz, C), 131.9 (*J*_CPt_ = 53 Hz, CH), 131.2 (*J*_CPt_ = 53 Hz, CH), 130.1 (*J*_CPt_ = 46 Hz, CH), 129.9 (CH), 128.6 (C), 126.3 (CH), 125.8 (2 × CH), 124.6 (*J*_CPt_ = 16 Hz, CH), 120.9 (*J*_CPt_ = 21 Hz, CH), 115.9 (*J*_CPt_ = 33 Hz, CH), 37.5 (NCH_3_), 31.7 (CH_2_), 24.1 (CH_2_), 23.1 (CH_2_), 14.1 (CH_3_). Anal. Calcd for C_24_H_24_Cl_2_N_4_Pt·0.33CH_2_Cl_2_: C, 44.10; H, 3.75; N, 8.45. Found: C, 44.08; H, 3.82; N, 8.70.

#### (*OC*-6-54)-[PtCl_2_(tpy)(trz)] (**3d**)

White solid, obtained from cis-C,C*-**2d** (74 mg, 0.121 mmol) and PhICl_2_ (36 mg, 0.133 mmol). Yield: 50 mg, 56%. ^1^H NMR (600 MHz, CD_2_Cl_2_): δ 9.76 (d with satellites, *J*_HH_ = 6.2 Hz, *J*_HPt_ = 14 Hz, 1H, H_arom_), 8.10–8.06 (m, 1H, H_arom_), 8.00 (d, *J*_HH_ = 8.2 Hz, 1H, H_arom_), 7.62 (dd with satellites, *J*_HH_ = 7.9, 1.2 Hz, *J*_HPt_ = 10 Hz, 1H, H_arom_), 7.59 (d, *J*_HH_ = 7.9 Hz, 1H, H_arom_), 7.52 (ddd, *J*_HH_ = 7.2, 5.7, 1.3 Hz, 1H, H_arom_) 7.15–7.11 (m, 1H, H_arom_), 6.94 (d, *J*_HH_ = 7.9 Hz, 1H, H_arom_), 6.89 (td with satellites, *J*_HH_ = 7.7, 1.3 Hz, *J*_HPt_ = 8 Hz, 1H, H_arom_), 6.26 (s with satellites, *J*_HPt_ = 46 Hz, 1H, H_arom_), 6.16 (dd with satellites, *J*_HH_ = 7.8, 1.2 Hz, *J*_HPt_ = 44 Hz, 1H, H_arom_), 4.29 (s, 3H, NCH_3_), 3.52 (ddd, *J*_HH_ = 14.4, 10.2, 5.6 Hz, 1H, CH_2_), 3.32 (ddd, *J*_HH_ = 14.4, 10.2, 5.9 Hz, 1H, CH_2_), 2.14 (s, 3H, CH_3_), 1.91–1.75 (m, 2H, CH_2_), 1.56–1.47 (m, 2H, CH_2_), 0.98 (t, *J*_HH_ = 7.3 Hz, 3H, CH_3_). ^13^C APT NMR (151 MHz, CD_2_Cl_2_): δ 163.0 (*J*_CPt_ = 50 Hz, C), 148.5 (CH), 145.5 (*J*_CPt_ = 82 Hz, C), 142.8 (*J*_CPt_ = 53 Hz, C), 141.2 (C), 140.5 (CH), 140.0 (C), 138.6 (C), 132.6 (C), 131.9 (*J*_CPt_ = 53 Hz, CH), 130.1 (*J*_CPt_ = 45 Hz, CH), 130.0 (CH), 128.7 (*J*_CPt_ = 817 Hz, C), 126.7 (CH), 126.2 (CH), 125.6 (*J*_CPt_ = 32 Hz, CH), 124.1 (*J*_CPt_ = 17 Hz, CH), 120.6 (*J*_CPt_ = 22 Hz, CH), 116.0 (*J*_CPt_ = 33 Hz, CH), 37.6 (NCH_3_), 31.8 (CH_2_), 24.1 (CH_2_), 23.1 (CH_2_), 21.9 (CH_3_), 14.2 (CH_3_). Anal. Calcd for C_25_H_26_Cl_2_N_4_Pt·CH_2_Cl_2_: C, 42.58; H, 3.85; N, 7.61. Found: C, 42.21; H, 3.96; N, 7.57.

#### (*OC*-6-54)-[PtCl_2_(thpy)(trz)] (**3e**)

Beige solid, obtained from cis-C,C*-**2e** (70 mg, 0.116 mmol) and PhICl_2_ (38 mg, 0.139 mmol). Yield: 28 mg, 38%. ^1^H NMR (600 MHz, CD_2_Cl_2_): δ 9.63–9.58 (m, 1H, H_arom_), 8.01 (td, *J*_HH_ = 7.8, 1.6 Hz, 1H, H_arom_), 7.68 (d, *J*_HH_ = 7.9 Hz, 1H, H_arom_), 7.59 (dd with satellites, *J*_HH_ = 7.9, 1.4 Hz, *J*_HPt_ ∼ 10 Hz, 1H, H_arom_), 7.42 (ddd, *J*_HH_ = 7.3, 5.2, 1.2 Hz, 1H, H_arom_), 7.29 (d with satellites, *J*_HH_ = 4.8 Hz, *J*_HPt_ = 9 Hz, 1H, H_arom_), 7.14 (ddd, *J*_HH_ = 8.4, 7.5, 1.0 Hz, 1H, H_arom_), 6.93 (td, *J*_HH_ = 7.7, 1.3 Hz, *J*_HPt_ ∼ 7 Hz,1H, H_arom_), 6.26 (dd with satellites, *J*_HH_ = 7.9, 1.2 Hz, *J*_HPt_ ∼ 43 Hz, 1H, H_arom_), 6.22 (d with satellites, *J*_HH_ = 4.9 Hz, *J*_HPt_ = 18 Hz, 1H, H_arom_), 4.26 (s, 3H, NCH_3_), 3.51 (ddd, *J*_HH_ = 14.3, 10.1, 5.9 Hz, 1H, CH_2_), 3.29 (ddd, *J*_HH_ = 14.4, 10.2, 6.1 Hz, 1H, CH_2_), 1.83–1.73 (m, 2H, CH_2_), 1.55–1.47 (m, 2H, CH_2_), 0.98 (t, *J*_HH_ = 7.4 Hz, 3H, CH_3_). ^13^C APT NMR (151 MHz, CD_2_Cl_2_): δ 158.8 (*J*_CPt_ = 39 Hz, C), 148.5 (CH), 146.1 (*J*_CPt_ = 86 Hz, C), 140.9 (CH), 138.1 (C), 130.3 (*J*_CPt_ = 43 Hz, CH), 130.2 (CH), 129.7 (*J*_CPt_ = 64 Hz, CH), 128.8 (*J*_CPt_ = 83 Hz, CH), 128.3 (C), 127.6 (C), 126.4 (CH), 122.3 (*J*_CPt_ = 15 Hz, CH), 119.9 (*J*_CPt_ = 17 Hz, CH), 115.8 (*J*_CPt_ = 33 Hz, CH), 37.5 (NCH_3_), 32.0 (CH_2_), 24.0 (CH_2_), 23.1 (CH_2_), 14.1 (CH_3_) (two of the quaternary carbons were not observed). Anal. Calcd for C_22_H_22_Cl_2_N_4_PtS: C, 41.26; H, 3.46; N, 8.75; S, 5.01. Found: C, 41.26; H, 3.37; N, 8.67; S, 5.07.

#### (*OC*-6-41)-[PtCl_3_(tpy)(trzH)] (**4d**)

To a solution of cis-C,C*-**2d** (73 mg, 0.098 mmol) in CH_2_Cl_2_ (5 mL) was added PhICl_2_ (30 mg, 0.109 mmol), and the mixture was stirred at room temperature for 30 min. The mixture was concentrated under reduced pressure (2 mL). To the mixture was added Et_2_O (20 mL), whereupon a pale-yellow precipitate formed that was filtered off. Extraction with MeOH (2 × 1 mL) and evaporation to dryness led to an analytically pure sample of **4d**. ^1^H NMR (400 MHz, CD_2_Cl_2_): δ 9.72–9.67 (1H, H_arom_), 7.95–7.80 (4H, H_arom_), 7.64–7.59 (1H, H_arom_), 7.53–7.46 (1H, H_arom_), 7.43–7.32 (3H, H_arom_), 7.07–7.02 (1H, H_arom_), 6.71 (*J*_PtH_ = 33 Hz, 1H, H_arom_), 4.26 (3H, NCH_3_), 3.36–3.27 (1H, CH_2_), 2.82–2.72 (1H, CH_2_), 2.37 (3H, CH_3_), 2.14–1.89 (2H, CH_2_), 1.76–1.60 (1H, CH_2_), 1.40–1.30 (1H, CH_2_), 0.95–0.85 (3H, CH_3_) (signal multiplicity could not be determined for this complex because its very low solubility resulted in a poorly resolved spectrum). Anal. Calcd for C_25_H_27_Cl_3_N_4_Pt·CH_2_Cl_2_: C, 40.56; H, 3.80; N, 7.28. Found: C, 40.66; H, 3.79; N, 7.37.

#### (*OC*-6-42)-[PtCl_2_(ppz)(trz)] (**5a**)

To a solution of trans-C,C*-**2a** (100 mg, 0.170 mmol) in CH_2_Cl_2_ (10 mL) was added PhICl_2_ (47 mg, 0.170 mmol), and the mixture was stirred for 30 min. The partial evaporation of the solvent under reduced pressure (2 mL) and the addition of Et_2_O (20 mL) led to the precipitation of a white solid, which was filtered off, washed with MeOH (3 × 1 mL) and Et_2_O (2 × 3 mL), and vacuum dried to give **5a**. Yield: 25 mg, 23%. ^1^H NMR (600 MHz, CD_2_Cl_2_): δ 8.17 (dd, *J*_HH_ = 7.5, 1.2 Hz, 1H, H_arom_), 8.06 (d, *J*_HH_ = 2.9 Hz, 1H, H_arom_), 7.65 (dd, *J*_HH_ = 7.9, 1.5 Hz, 1H, H_arom_), 7.44–7.36 (m, 3H, H_arom_), 7.11–7.05 (m, 2H, H_arom_), 6.87 (td, *J*_HH_ = 7.6, 1.5 Hz, 1H, H_arom_), 6.47 (t, *J*_HH_ = 2.7 Hz, 1H, H_arom_), 6.39 (dd with satellites, *J*_HH_ = 7.9, 1.2 Hz, *J*_PtH_ = 43 Hz, 1H, H_arom_), 4.25 (s, 3H, NCH_3_), 3.45 (ddd, *J*_HH_ = 14.3, 9.9, 6.1 Hz, 1H, CH_2_), 3.34 (ddd, *J*_HH_ = 14.3, 9.9, 6.0 Hz, 1H, CH_2_), 1.85–1.76 (m, 2H, CH_2_), 1.56–1.50 (m, 2H, CH_2_), 0.99 (t, *J*_HH_ = 7.4 Hz, 3H, CH_3_). ^13^C APT NMR (151 MHz, CD_2_Cl_2_): δ 150.8 (C), 149.2 (C), 142.4 (C), 142.1 (C), 141.3 (C), 138.7 (*J*_CPt_ = 45 Hz, CH), 134.3 (CH), 132.6 (CH), 130.1 (*J*_CPt_ = 46 Hz, CH), 128.4 (*J*_CPt_ = 24 Hz, CH), 127.8 (*J*_CPt_ = 19 Hz, CH), 126.7 (CH), 126.1 (CH), 122.6 (C), 116.4 (*J*_CPt_ = 25 Hz, CH), 112.7 (CH), 109.0 (*J*_CPt_ = 25 Hz, CH), 37.0 (NCH_3_), 32.5 (CH_2_), 23.9 (CH_2_), 23.1 (CH_2_), 14.2 (CH_3_). Anal. Calcd for C_22_H_23_Cl_2_N_5_Pt·0.25CH_2_Cl_2_: C, 41.45; H, 3.67; N, 10.86. Found: C, 41.39; H, 3.91; N, 11.04.

#### (*OC*-6-42)-[PtCl_2_(tpy)(trz)] (**5d**) and (*OC*-6-43)-[PtCl_3_(tpy)(trzH)] (**6d**)

To a solution of trans-C,C*-**2d** (160 mg, 0.261 mmol) in CH_2_Cl_2_ (15 mL) was added PhICl_2_ (79 mg, 0.287 mmol), and the mixture was stirred for 30 min. The partial evaporation of the solvent under reduced pressure (2 mL) and the addition of Et_2_O (20 mL) led to the precipitation of a pale-yellow solid, which was collected by filtration. The ^1^H NMR spectrum of this material revealed a mixture of complexes **5d** and **6d** in a 1:4 molar ratio, which was stirred in MeOH (20 mL) for 10 min to give a suspension. The insoluble white solid was collected by filtration, washed with Et_2_O (2 × 3 mL), and vacuum-dried to give **5d**. The filtrate was evaporated to dryness, and the pale-yellow residue was washed with Et_2_O (3 × 3 mL) and vacuum-dried to give **6d**.

##### Data for **5d**

Yield: 18 mg, 11%. ^1^H NMR (600 MHz, CD_2_Cl_2_): δ 8.06 (br s, 1H, H_arom_), 7.94 (d with satellites, *J*_HH_ = 5.9 Hz, *J*_HPt_ = 35 Hz, 1H, H_arom_), 7.85 (d, *J*_HH_ = 7.5 Hz, 1H, H_arom_), 7.75 (ddd, *J*_HH_ = 8.5, 7.3, 1.5 Hz, 1H, H_arom_), 7.67 (d, *J*_HH_ = 7.9 Hz, 1H, H_arom_), 7.64 (dd, *J*_HH_ = 7.9, 1.5 Hz, 1H, H_arom_), 7.21 (dd, *J*_HH_ = 7.9, 1.2 Hz, 1H, H_arom_), 7.07 (td, *J*_HH_ = 7.6, 1.2 Hz, 1H, H_arom_), 6.89–6.83 (m, 2H, H_arom_), 6.37 (dd with satellites, *J*_HH_ = 8.0, 1.2 Hz, *J*_HPt_ = 42 Hz, 1H, H_arom_), 4.26 (s, 3H, NCH_3_), 3.42–3.38 (m, 2H, CH_2_), 1.92–1.76 (m, 2H, CH_2_), 1.56–1.50 (m, 2H, CH_2_), 0.99 (t, *J*_HH_ = 7.4 Hz, 3H, CH_3_). ^13^C APT NMR (151 MHz, CD_2_Cl_2_): δ 167.7 (C), 158.7 (C), 156.3 (C), 149.9 (CH), 148.6 (C), 143.2 (C), 142.7 (C), 140.4 (C), 140.1 (CH), 134.2 (CH), 132.1 (CH), 129.9 (*J*_CPt_ = 44 Hz, CH), 126.6 (CH), 126.5 (CH), 125.4 (CH), 123.4 (CH), 121.1 (CH), 116.3 (CH), 37.0 (NCH_3_), 32.3 (CH_2_), 24.1 (CH_2_), 23.2 (CH_2_), 22.5 (CH_3_), 14.2 (CH_3_). Anal. Calcd for C_25_H_26_Cl_2_N_4_Pt: C, 46.30; H, 4.04; N, 8.64. Found: C, 46.19; H, 4.16; N, 8.34.

##### Data for **6d**

Yield: 120 mg, 67%. ^1^H NMR (600 MHz, CD_2_Cl_2_): δ 9.56 (d with satellites, *J*_HH_ = 6.0 Hz, *J*_HPt_ = 31 Hz, 1H, H_arom_), 7.71 (t, *J*_HH_ = 8.0 Hz, 1H, H_arom_), 7.63 (s with satellites, *J*_HPt_ = 28 Hz, 1H, H_arom_), 7.31 (d, *J*_HH_ = 8.0 Hz, 1H, H_arom_), 7.24–7.16 (m, 2H, H_arom_), 7.01–6.96 (m, 2H, H_arom_), 6.84 (br t, *J*_HH_ = 7.6 Hz, 1H, H_arom_), 6.77 (br t, *J*_HH_ = 7.6 Hz, 1H, H_arom_), 6.38 (d, *J*_HH_ = 7.6 Hz, 1H, H_arom_), 6.14 (d, *J*_HH_ = 7.9 Hz, 1H, H_arom_), 4.08 (s, 3H, NCH_3_), 3.85 (ddd, *J*_HH_ = 14.6, 12.3, 4.4 Hz, 1H, CH_2_), 3.28 (ddd, *J*_HH_ = 14.6, 12.3, 4.5 Hz, 1H, CH_2_), 2.41 (s, 3H, CH_3_), 1.83–1.68 (m, 2H, CH_2_), 1.66–1.58 (m, 2H, CH_2_), 1.07 (t, *J*_HH_ = 7.3 Hz, 3H, CH_3_). ^13^C APT NMR (151 MHz, CD_2_Cl_2_): δ 164.9 (C), 150.1 (C), 149.0 (CH), 142.7 (C), 142.6 (C), 140.7 (CH), 139.9 (C), 137.4 (C), 131.8 (*J*_CPt_ = 26 Hz, CH), 129.8 (CH), 129.2 (CH), 129.0 (CH), 127.3 (CH), 127.0 (CH), 126.1 (*J*_CPt_ = 27 Hz, CH), 125.8 (CH), 123.4 (*J*_CPt_ = 27 Hz, CH), 121.4 (*J*_CPt_ = 30 Hz, CH), 117.6 (C), 37.9 (NCH_3_), 31.9 (CH_2_), 27.1 (CH_2_), 23.4 (CH_2_), 22.3 (CH_3_), 14.2 (CH_3_). Anal. Calcd for C_25_H_27_Cl_3_N_4_Pt: C, 43.84; H, 3.97; N, 8.18. Found: C, 43.79; H, 4.11; N, 8.01.

## Supporting Information Available

Photophysical characterization, X-ray structure determinations, electrochemical measurements, crystallographic data, NMR spectra, excitation and emission spectra, and computational methods and data (PDF)

## Accession Codes

CCDC 2061761–2061762 and 2061767–2061769 contain the supplementary crystallographic data for this paper. These data can be obtained free of charge via www.ccdc.cam.ac.uk/data_request/cif, or by emailing data_request@ccdc.cam.ac.uk, or by contacting The Cambridge Crystallographic Data Centre, 12 Union Road, Cambridge CB2 1EZ, UK; fax: +44 1223 336033.
