# Supplementary material for: Strongly Luminescent Pt(IV) Complexes with a Mesoionic N-Heterocyclic Carbene Ligand: Tuning Their Photophysical Properties
Source: Inorg Chem. 2021 May 10;60(11):7900–13. doi: 10.1021/acs.inorgchem.1c00410 (PMC8893362; doi:10.1021/acs.inorgchem.1c00410)
Supplement: Supplementary file 1 — ic1c00410_si_001.pdf [file ic1c00410_si_001.pdf]

SUPPORTING INFORMATION

Strongly Luminescent Pt(IV) Complexes with a Mesoionic *N*-Heterocyclic Carbene Ligand: Tuning their Photophysical Properties

Ángela Vivancos,[†] Adrián Jiménez-García,[†] Delia Bautista[‡] and Pablo González-Herrero*[,†]

[†]Departamento de Química Inorgánica, Facultad de Química, Universidad de Murcia, Campus de Espinardo, 19, 30100 Murcia, Spain.

[‡]Área Científica y Técnica de Investigación, Universidad de Murcia, Campus de Espinardo, 21, 30100 Murcia, Spain.

Contents:

1. Photophysical characterization 2
2. X-Ray structure determinations 2
3. Electrochemical measurements 4
4. NMR spectra of new compounds 5
5. Excitation and emission spectra 31
6. Computational methods 34
7. Computational data 35
   7.1. Complex **3d** 35
   7.2. Complex (*OC*-6-33)-[$PtCl_2(tpy)_2$] 40
   7.3. Complex **5d** 45
   7.4. Supplementary computational data 49
8. References 54

1. Photophysical characterization

UV-vis absorption spectra were recorded on a Perkin-Elmer Lambda 750S spectrophotometer. Excitation and emission spectra were recorded on a Jobin Yvon Fluorolog 3-22 spectrofluorometer. Emission data in PMMA matrices were registered using quartz slides as sample holders. Lifetimes were determined with an IBH FluoroHub controller in MCS mode and the Fluorolog's FL-1040 phosphorimeter pulsed xenon lamp as excitation source; the estimated uncertainty is ±10% or better. Emission quantum yields (Φ) were determined using a Hamamatsu C11347 Absolute PL Quantum Yield Spectrometer; the estimated uncertainty is ±5% or better. Unless otherwise specified, emission data were obtained under rigorous exclusion of oxygen, by bubbling argon through the solutions for 30 min or placing the PMMA films under argon.

2. X-Ray structure determinations

Single crystals of *cis-C,C**-**2d**, **3d**·CH_2Cl_2, **3e**, **5a** and **5d** suitable for X-ray diffraction were obtained by the liquid-liquid diffusion method from CH_2Cl_2/Et_2O. The data were collected on a Bruker D8 QUEST diffractometer with monochromated Mo-*K*α radiation performing φ and ω scans. The structures were solved by direct methods and refined anisotropically on F^2 using the program SHELXL-2018.[1] Methyl hydrogens were included as part of rigid idealized methyl groups allowed to rotate but not tip; other hydrogens were included using a riding model. Numerical details are given in the Supporting Information (Table S1). *Special features of refinement:* In **3d**, the structure contains one resolved molecule of dichloromethane that is disordered over two positions, ca 55:45%. The butyl fragment is disordered over two positions with a ca. 54:46% occupancy distribution. In **3e**, the butyl ligand is disordered over two positions, 79:21%; the structure contains a poorly-resolved region of residual electron density, which could not be adequately modelled and therefore was "removed" using the program SQUEEZE,[2,3] which is part of the PLATON system. The void volume per cell was 136 Å^3, with a void electron count per cell of 112. This additional solvent was not taken account of when calculating derived parameters such as the formula weight, because the nature of the solvent was uncertain.

Table S1. Crystallographic data for *cis-C,C**-**2d**, **3d**·CH_2Cl_2, **3e**, **5a** and **5d**.

	*cis-C,C**-**2d**	**3d**·CH_2Cl_2	**3e**	**5a**	**5d**
formula	$C_{25}H_{27}ClN_4Pt$	$C_{26}H_{28}Cl_4N_4Pt$	$C_{22}H_{22}Cl_2N_4PtS$	$C_{22}H_{23}Cl_2N_5Pt$	$C_{25}H_{26}Cl_2N_4Pt$
fw	614.04	733.41	640.48	623.44	648.49
T (K)	100(2)	100(2)	100(2)	100(2)	100(2)
λ	0.71073	0.71073	0.71073	0.71073	0.71073
cryst syst	Monoclinic	Triclinic	Triclinic	Monoclinic	Monoclinic
space group	$P2_1/c$	P-1	P-1	$P2_1/n$	$P2_1/c$
a (Å)	15.308(2)	8.1545(10)	8.0245(4)	11.4100(14)	13.1655(11)
b (Å)	9.8691(13)	10.9536(14)	10.9249(5)	12.5588(16)	11.8515(10)
c (Å)	17.150(3)	16.1105(19)	15.4363(7)	30.859(4)	16.5800(14)
α (°)	90	107.517(4)	74.001(2)	90	90
β (°)	115.884(4)	92.117(4)	88.469(2)	100.025(4)	111.814(4)
γ (°)	90	102.156(4)	75.476(2)	90	90
V (Å^3)	2331.0(6)	1333.7(3)	1257.96(10)	4354.5(9)	2401.8(4)
Z	4	2	2	8	4
ρ_{calcd} (Mg m^{-3})	1.750	1.826	1.691	1.902	1.793
μ (mm^{-1})	6.154	5.684	5.888	6.710	6.085
R1[a]	0.0155	0.0204	0.0182	0.0230	0.0142
wR2[b]	0.0350	0.0499	0.0476	0.0440	0.0300

[a]R1 = $\Sigma||F_o|-|F_c||/\Sigma|F_o|$ for reflections with $I > 2\sigma(I)$. [b]wR2 = $[\Sigma[w(F_o^2-F_c^2)^2/\Sigma[w(F_o^2)^2]]^{0.5}$ for all reflections; $w^{-1} = \sigma^2(F^2) + (aP)^2 + bP$, where $P = (2F_c^2 + F_o^2)/3$ and a and b are constants set by the program.

3. Electrochemical measurements

Cyclic voltammograms were registered with a potentiostat/galvanostat AUTOLAB-100 (Echo-Chemie, Utrecht), employing a three-electrode electrochemical cell equipped with a glassy carbon working electrode (Metrohm, 2 mm diameter), an Ag/AgCl/3 M KCl electrode reference, and a glassy carbon rod counter electrode. The measurements were carried out at 298 K under an argon atmosphere, using degassed 1 mM solutions of the complexes in extra-dry MeCN (Acros Organics) and 0.1 M $(Bu_4N)PF_6$ as the electrolyte. Before each experiment, the working electrode was polished with alumina slurry (0.05 μm) and rinsed with water and acetone. The electrodes were activated electrochemically in the background solution by means of several voltammetric cycles at 1 V s^{-1} between –2.8 V and 2.2 V. At the end of each experiment, the reference electrode was checked against the Fc^+/Fc redox couple. Potentials are given against the standard calomel electrode (SCE).

The HOMO/LUMO energies were estimated from the onset values of the oxidation and reduction waves, respectively, referenced against Fc^+/Fc (0.40 V vs SCE in MeCN), using a formal potential of 5.1 eV for the Fc^+/Fc couple in the Fermi scale:[4]

$$E_{\mathrm{HOMO}} = -(E_{onset,ox} + 5.1 - 0.4)\ \mathrm{eV};\ E_{\mathrm{LUMO}} = -(E_{onset,red} + 5.1 - 0.4)\ \mathrm{eV}$$

4. NMR spectra of new compounds

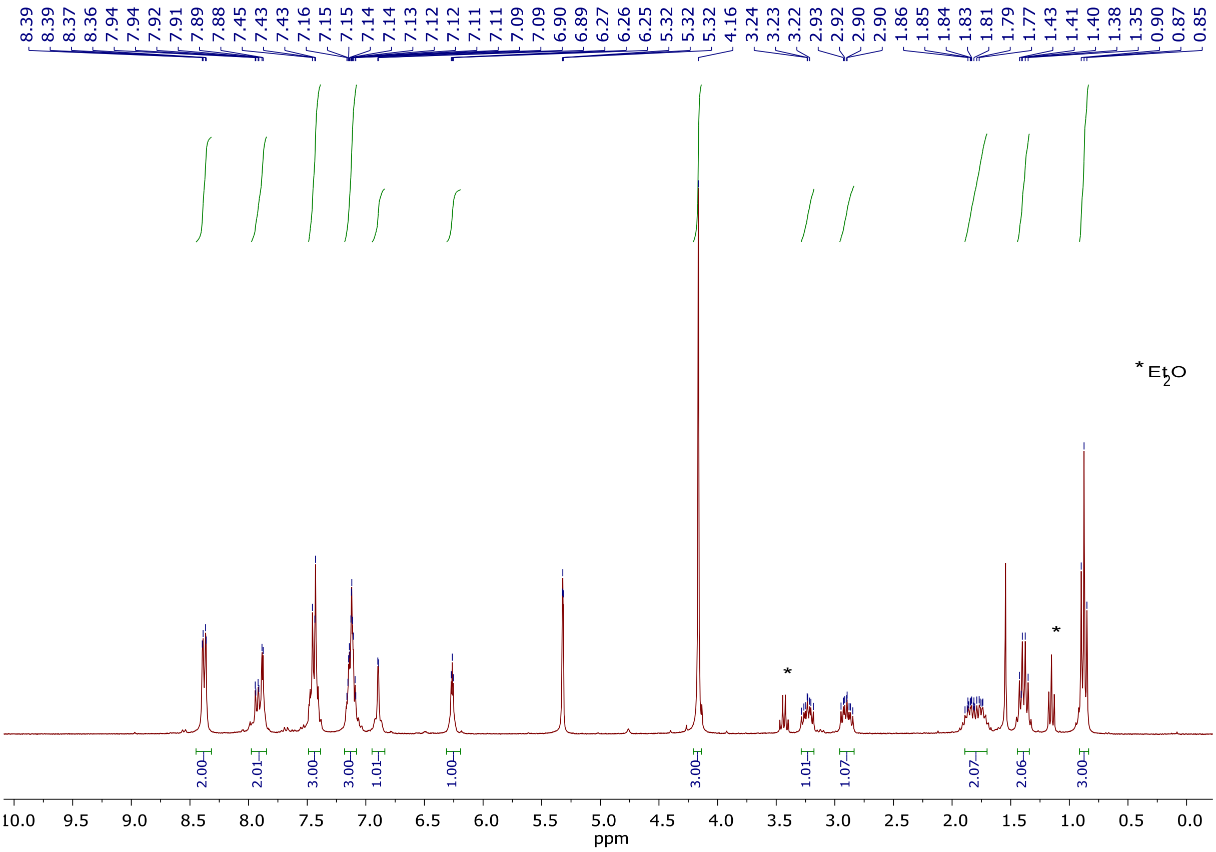

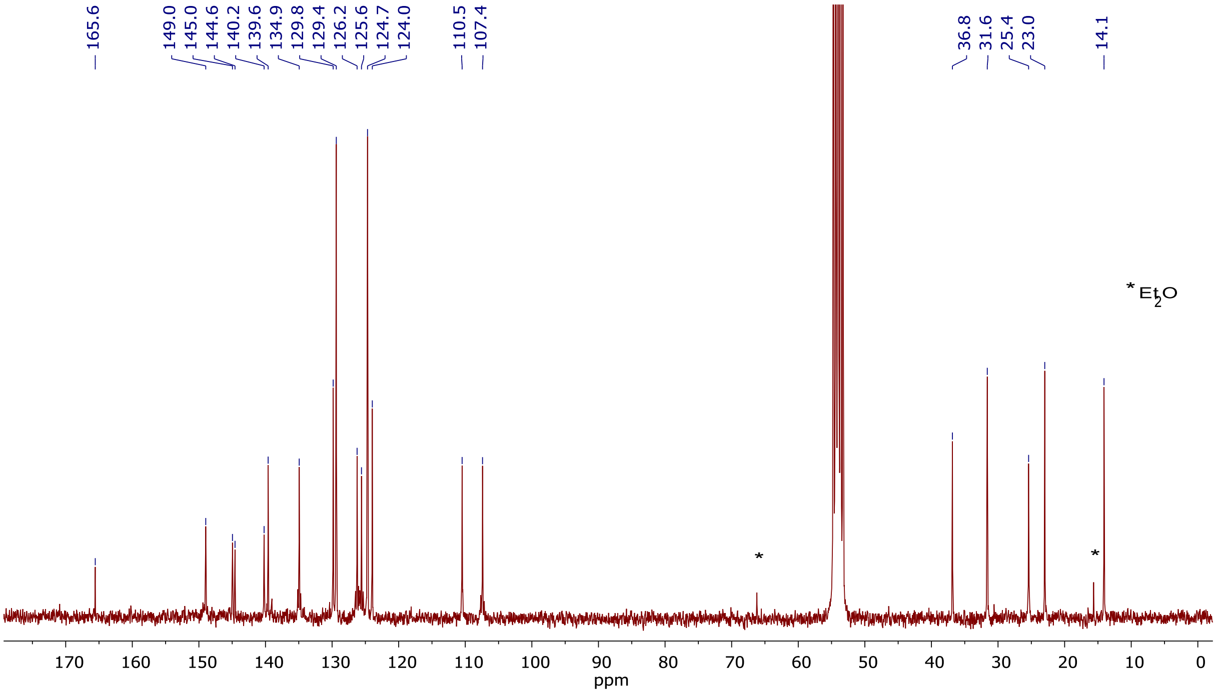

Figure S1. ^{1}H (top) and $^{13}C\{^1H\}$ APT (bottom) NMR spectra of complex *trans-C,C***-**2a** (CD_2Cl_2, 300 and 75 MHz, respectively)

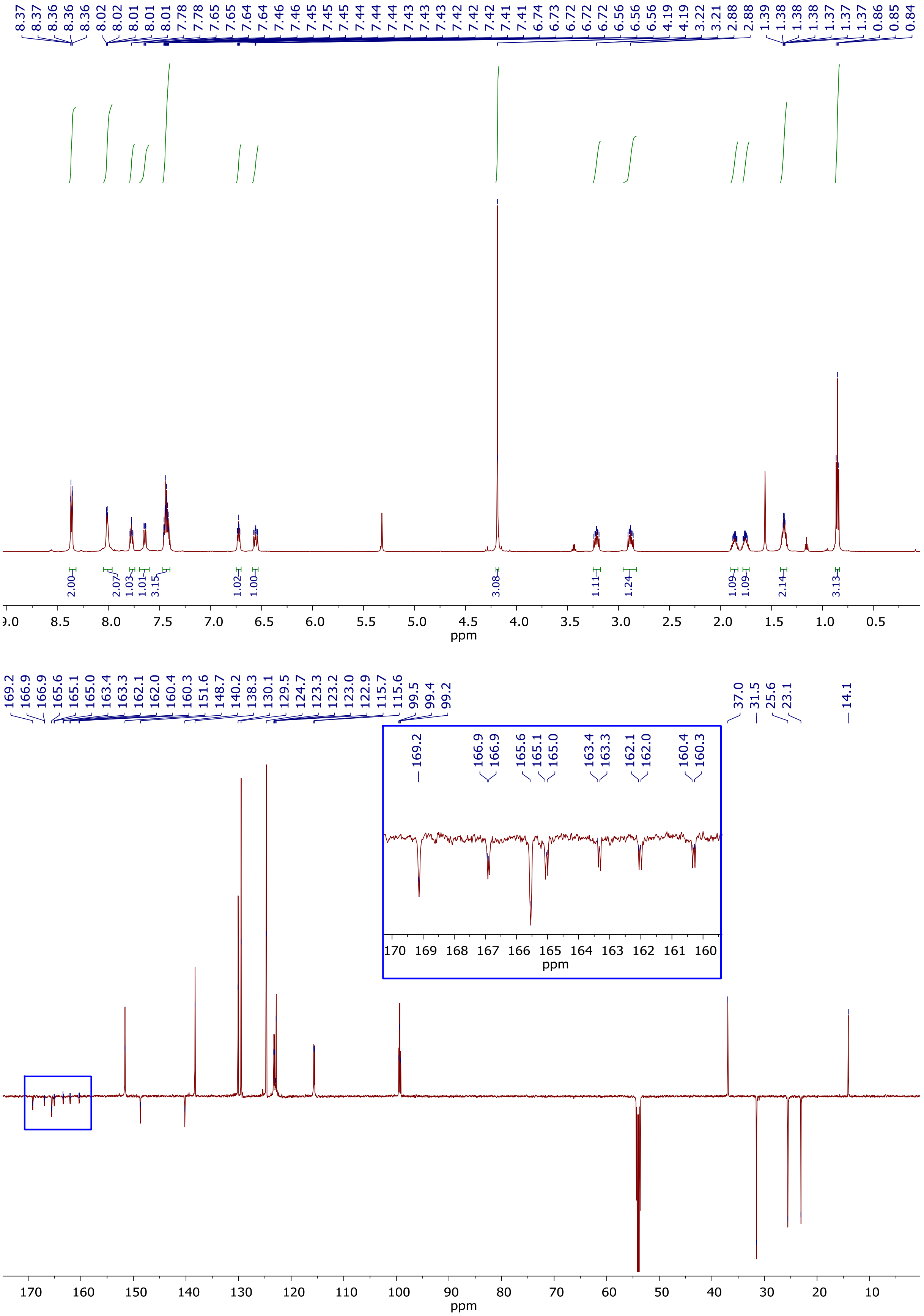

Figure S2. ^{1}H (top) and ^{13}C{^{1}H} APT (bottom) NMR spectra of complex *trans-C,C**-**2b** (CD_2Cl_2, 600 and 151 MHz, respectively).

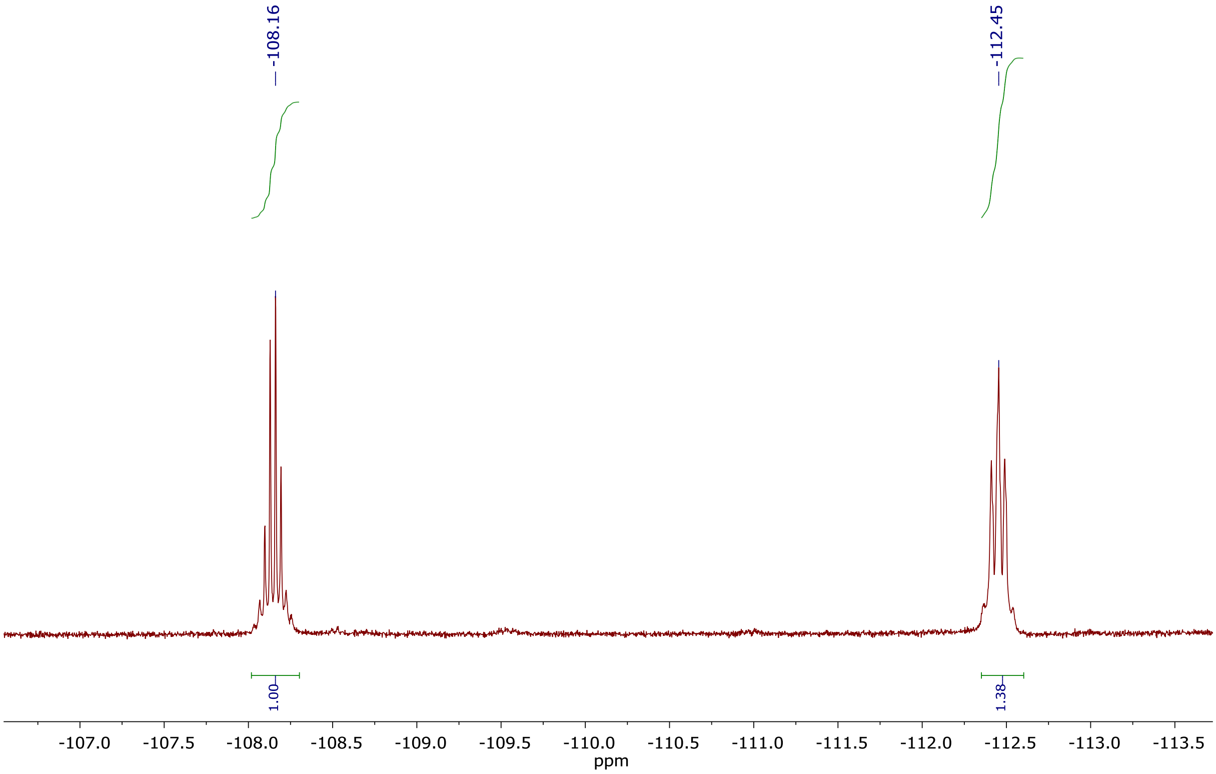

Figure S3. ^{19}F NMR spectrum of complex *trans-C,C**-**2b** (CD_2Cl_2, 282 MHz).

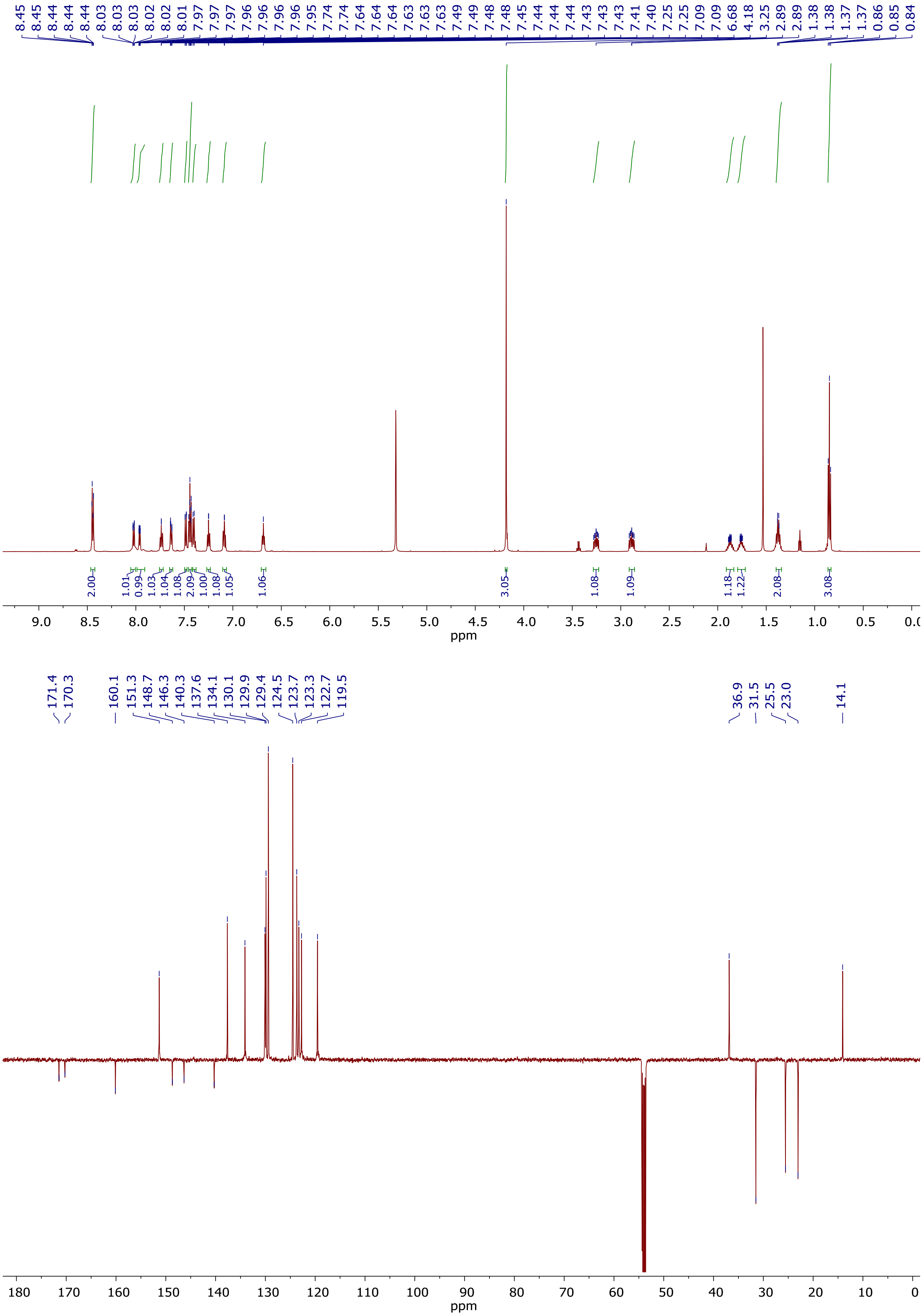

Figure S4. ^{1}H (top) and $^{13}C\{^{1}H\}$ APT (bottom) NMR spectra of complex *trans-C,C**-**2c** (CD_2Cl_2, 600 and 151 MHz, respectively).

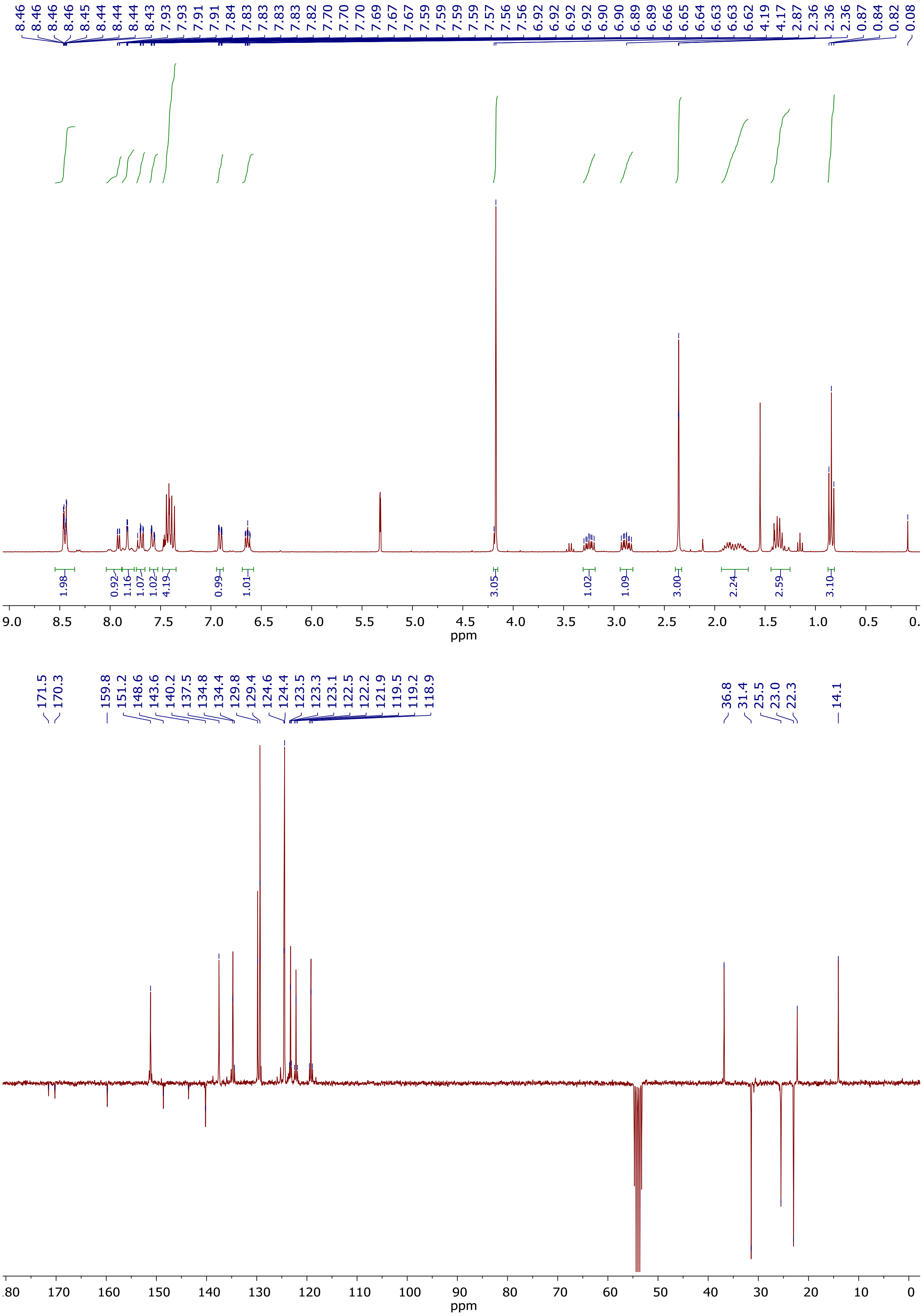

Figure S5. ^{1}H (top) and ^{13}C{^{1}H} APT (bottom) NMR spectra of complex *trans-C,C**-**2d** (CD_2Cl_2, 300 and 75 MHz, respectively).

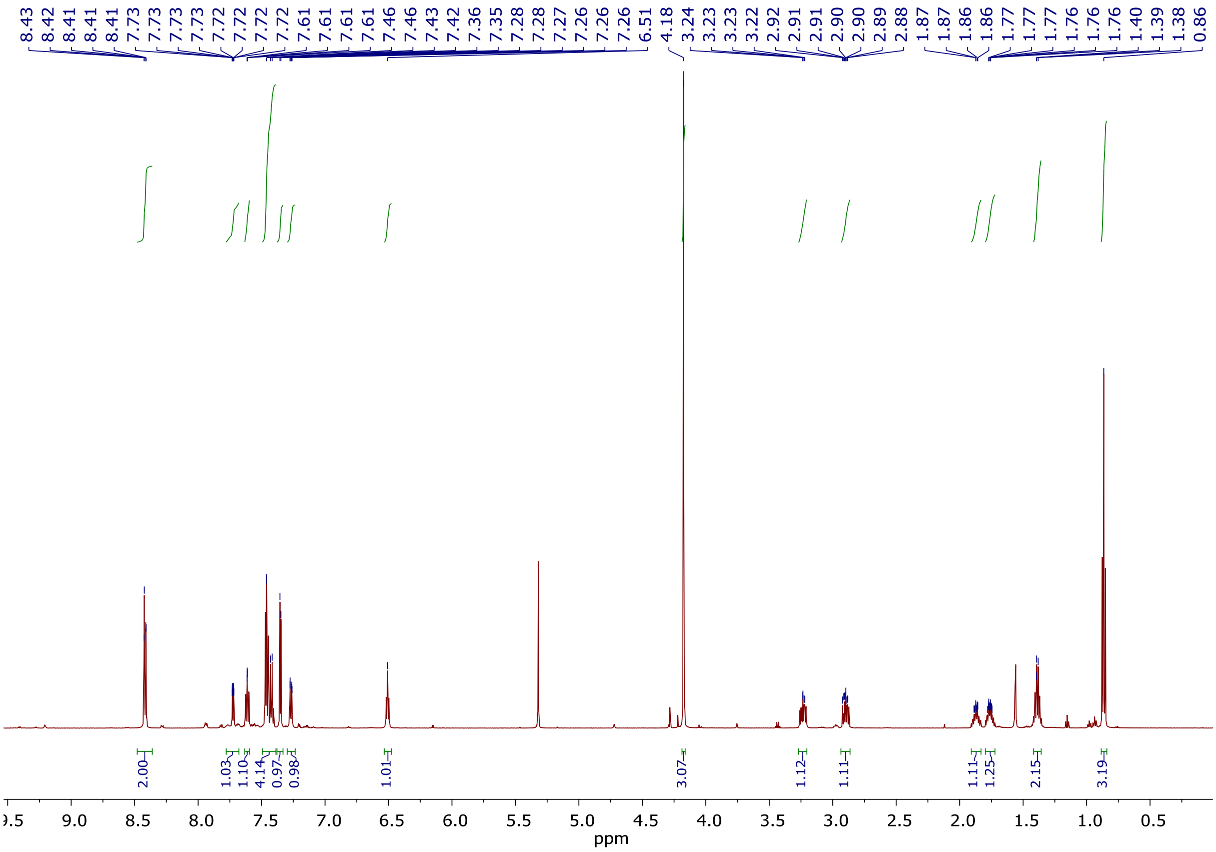

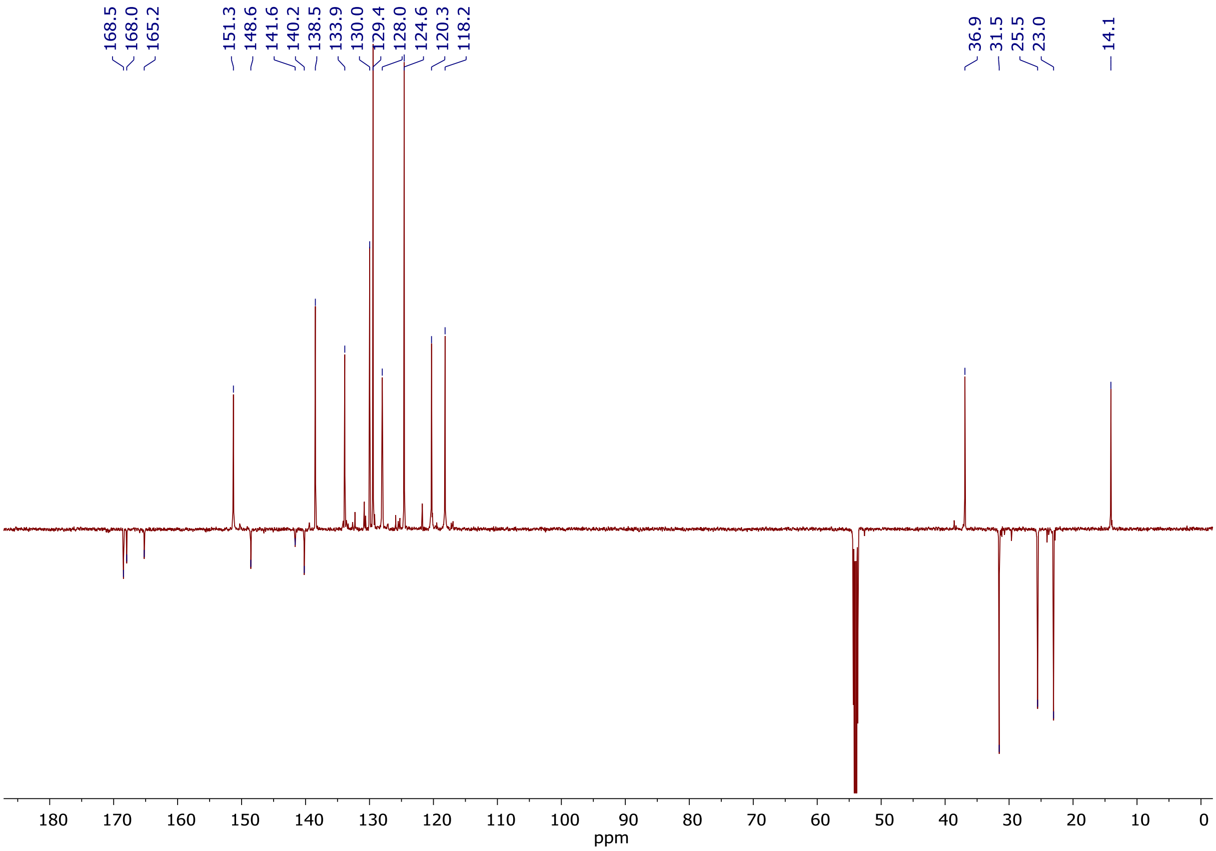

Figure S6. ^{1}H (top) and $^{13}C\{^{1}H\}$ APT (bottom) NMR spectra of complex *trans-C,C**-**2e** (CD_2Cl_2, 600 and 151 MHz, respectively).

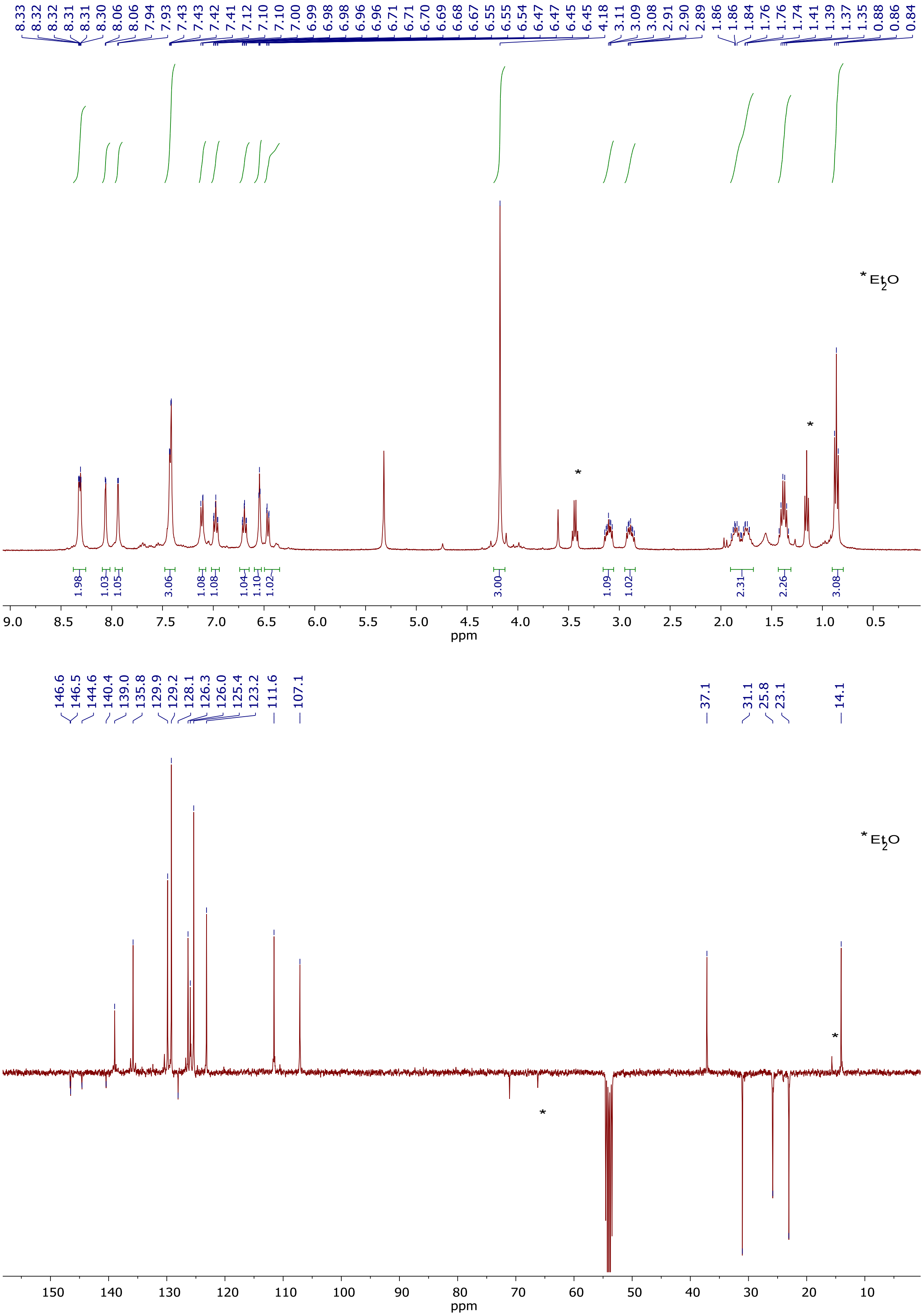

Figure S7. ^{1}H (top) and $^{13}C\{^{1}H\}$ APT (bottom) NMR spectra of complex *cis-C,C**-**2a** (CD_2Cl_2, 400 and 100 MHz, respectively).

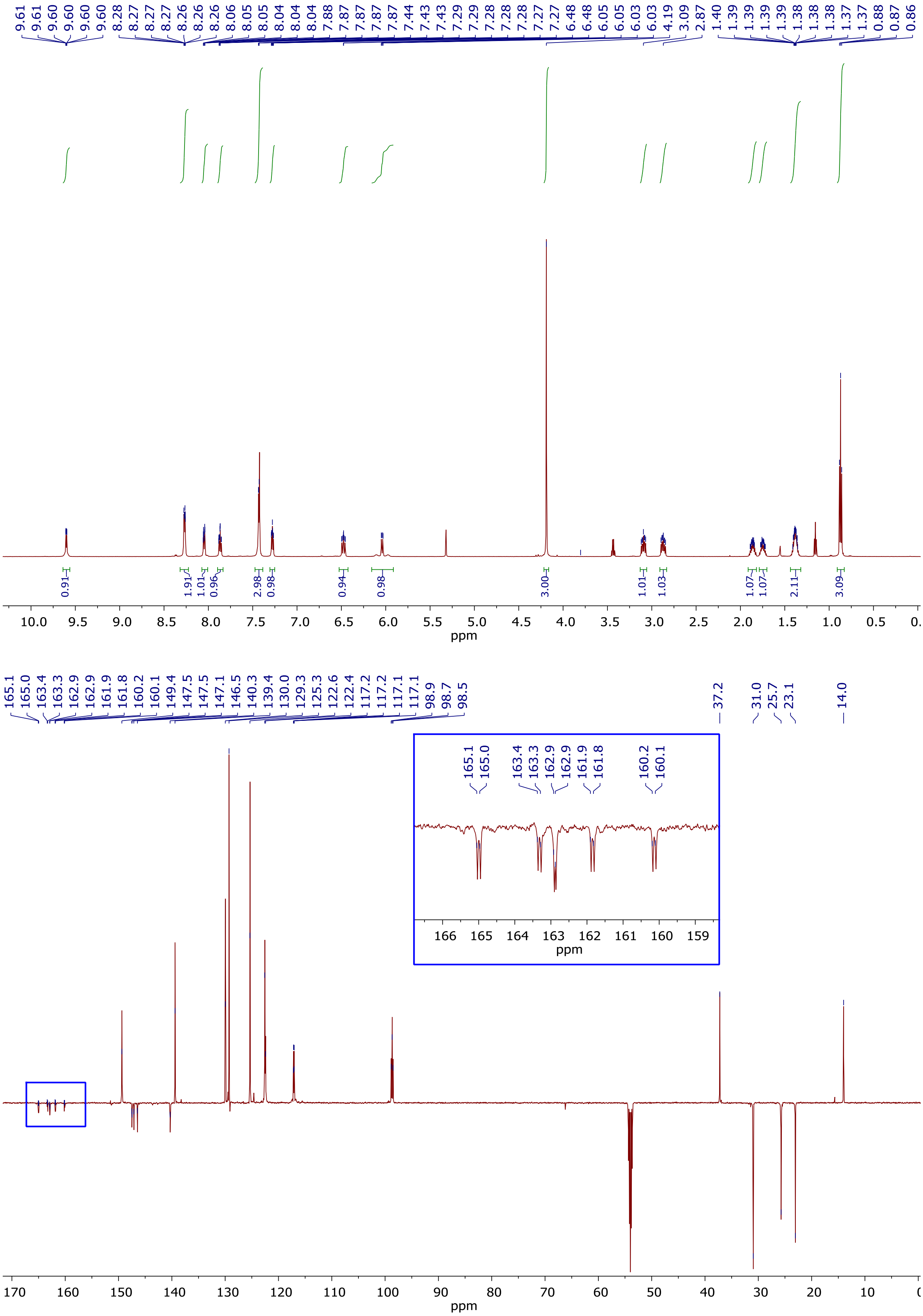

Figure S8. ^{1}H (top) and $^{13}C\{^{1}H\}$ APT (bottom) NMR spectra of complex *cis-C,C**-**2b** (CD_2Cl_2, 600 and 151 MHz, respectively).

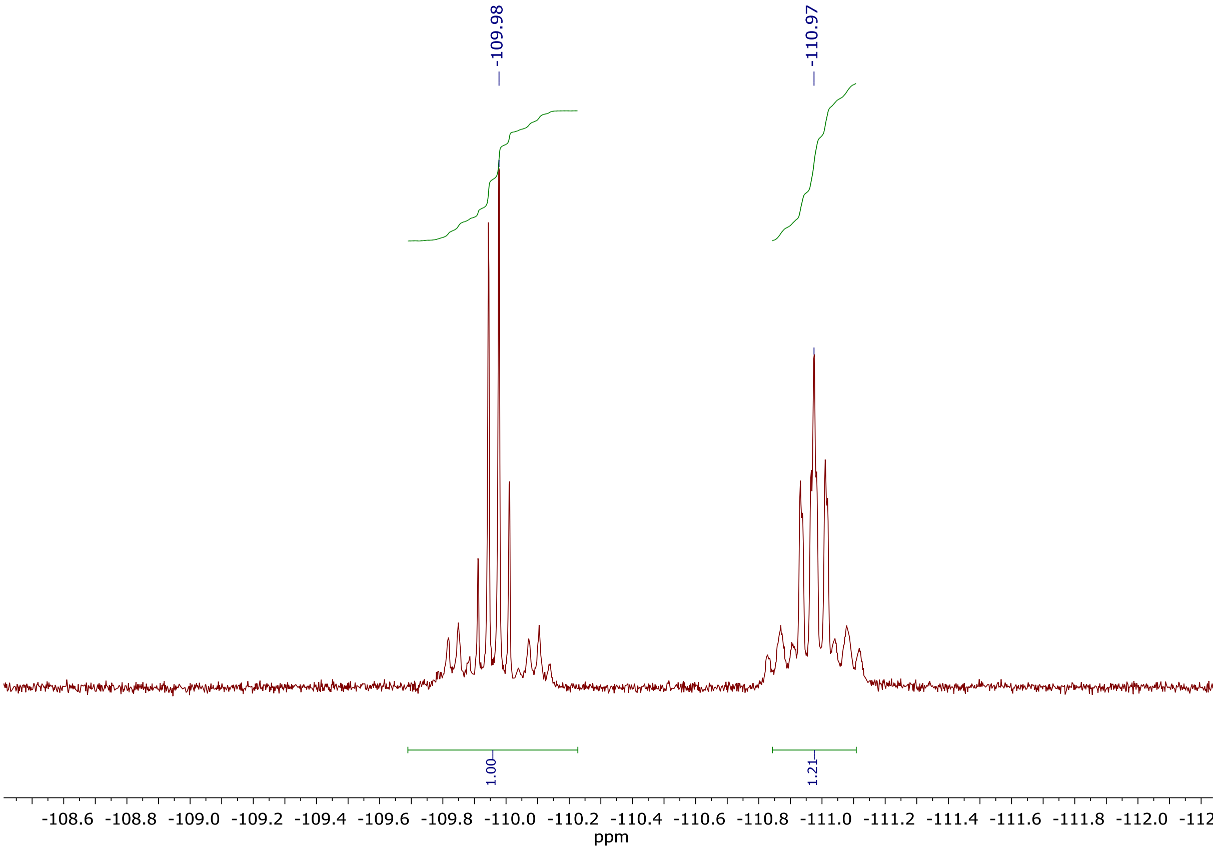

Figure S9. ^{19}F NMR spectrum of complex *cis-C,C**-**2b** (CD_2Cl_2, 282 MHz).

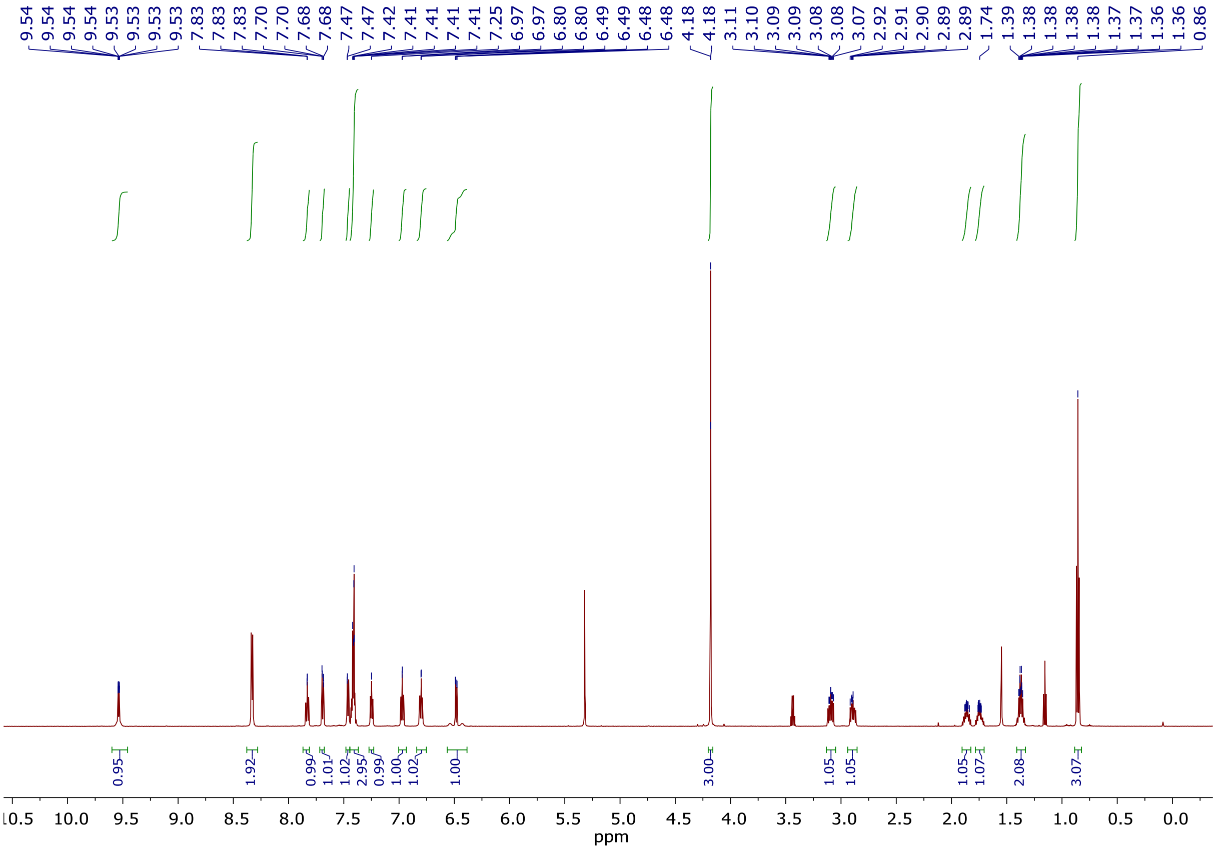

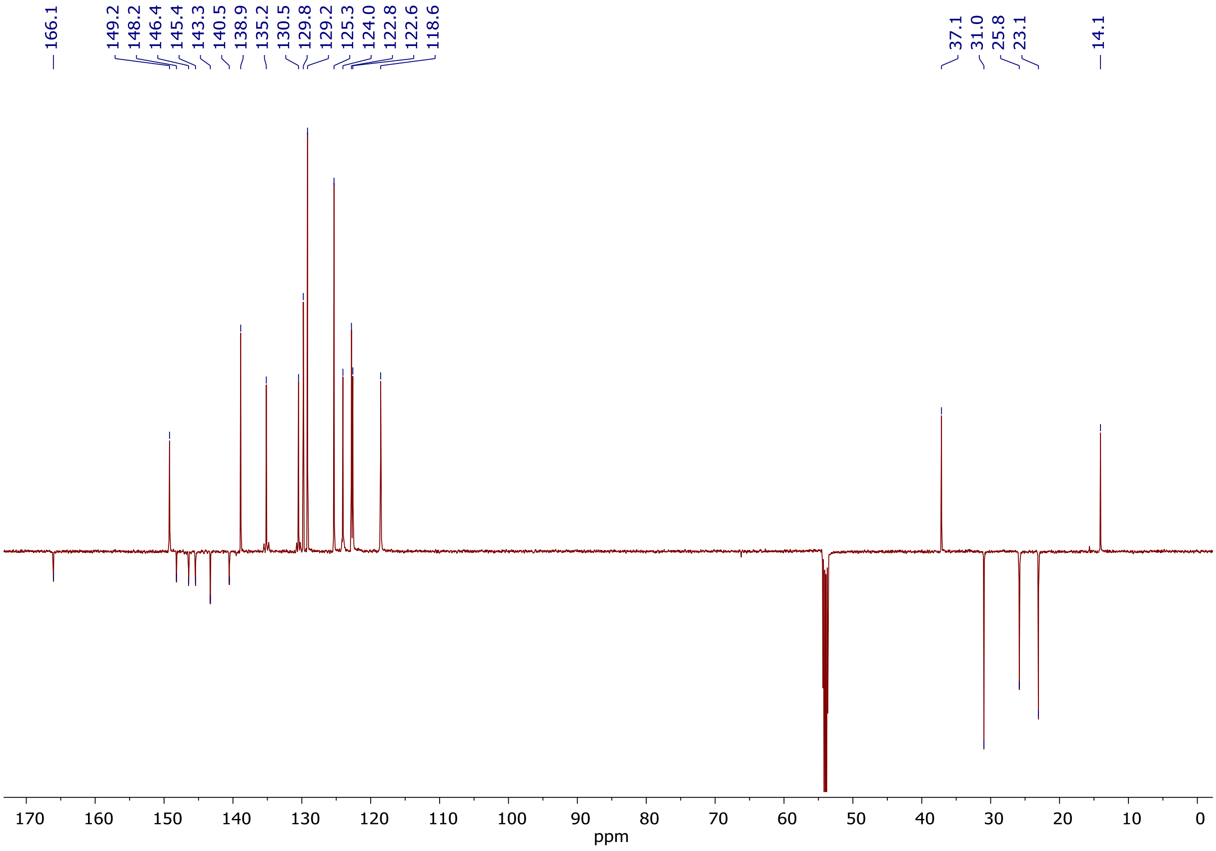

Figure S10. ^{1}H (top) and $^{13}C\{^{1}H\}$ APT (bottom) NMR spectra of complex *cis-C,C**-**2c** (CD_2Cl_2, 600 and 151 MHz, respectively).

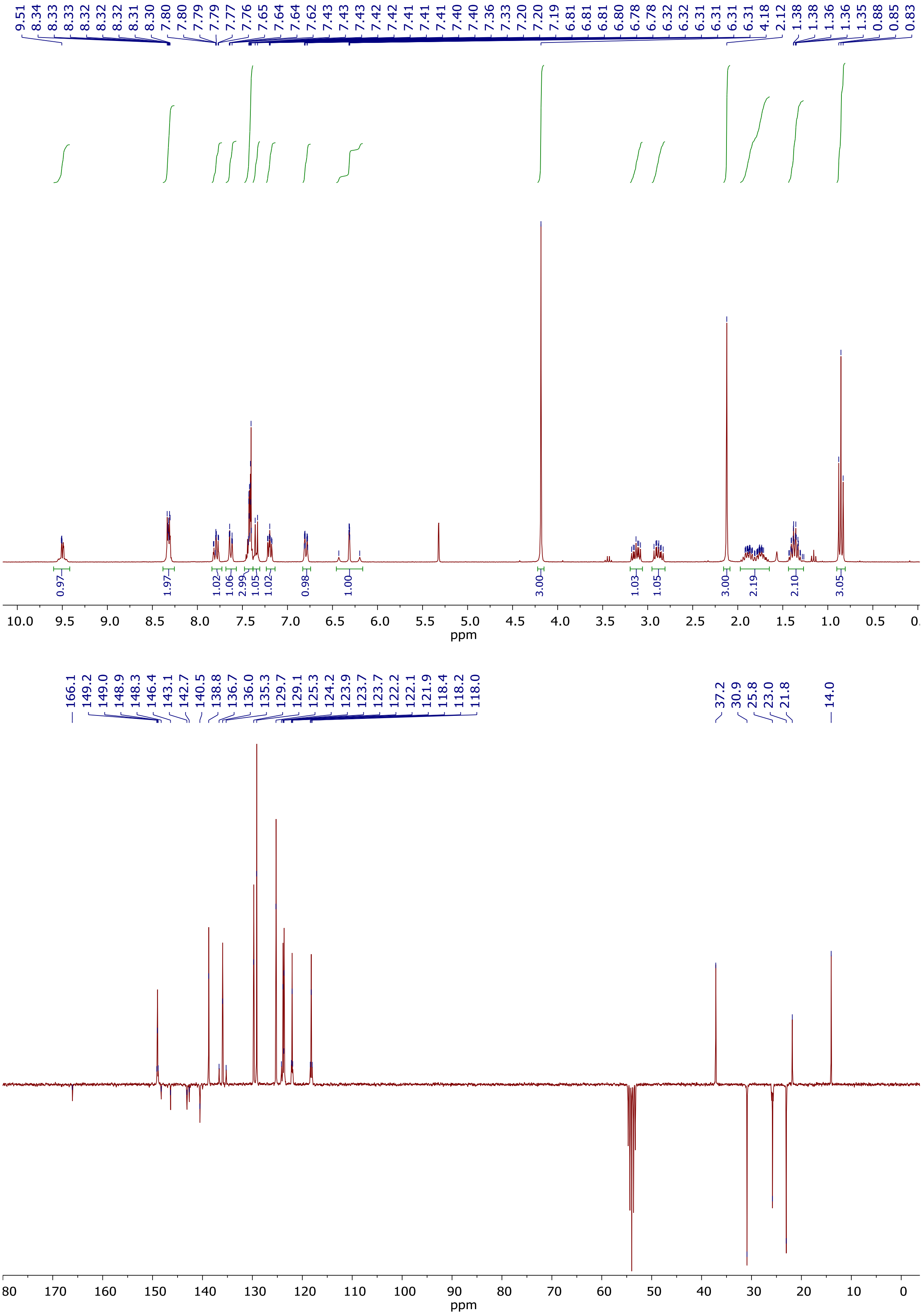

Figure S11. ^{1}H (top) and ^{13}C{^{1}H} APT (bottom) NMR spectra of complex *cis-C,C***-**2d** (CD_2Cl_2, 300 and 75 MHz, respectively).

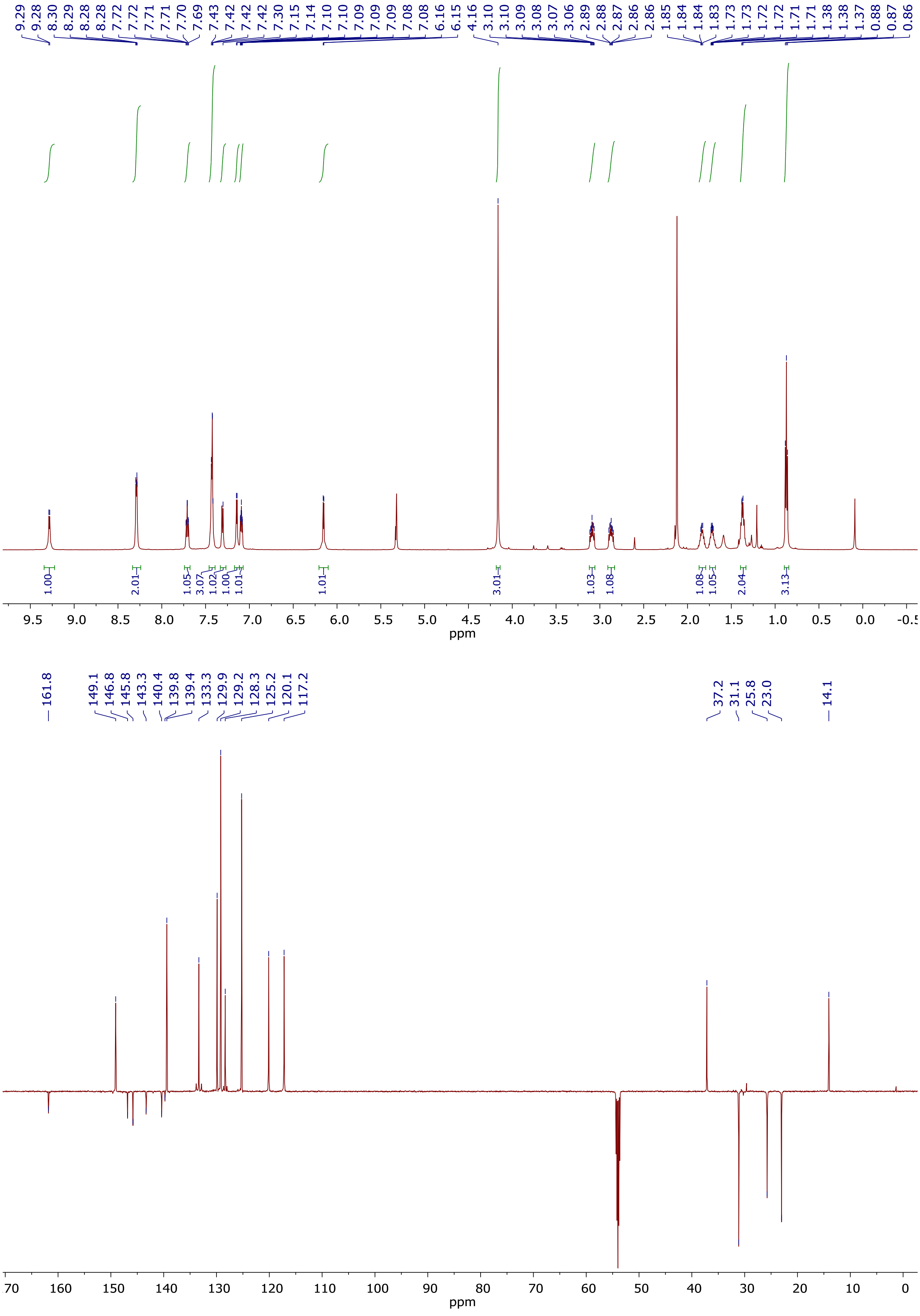

Figure S12. ^{1}H (top) and $^{13}C\{^{1}H\}$ APT (bottom) NMR spectra of complex *cis-C,C**-**2e** (CD_2Cl_2, 600 and 151 MHz, respectively).

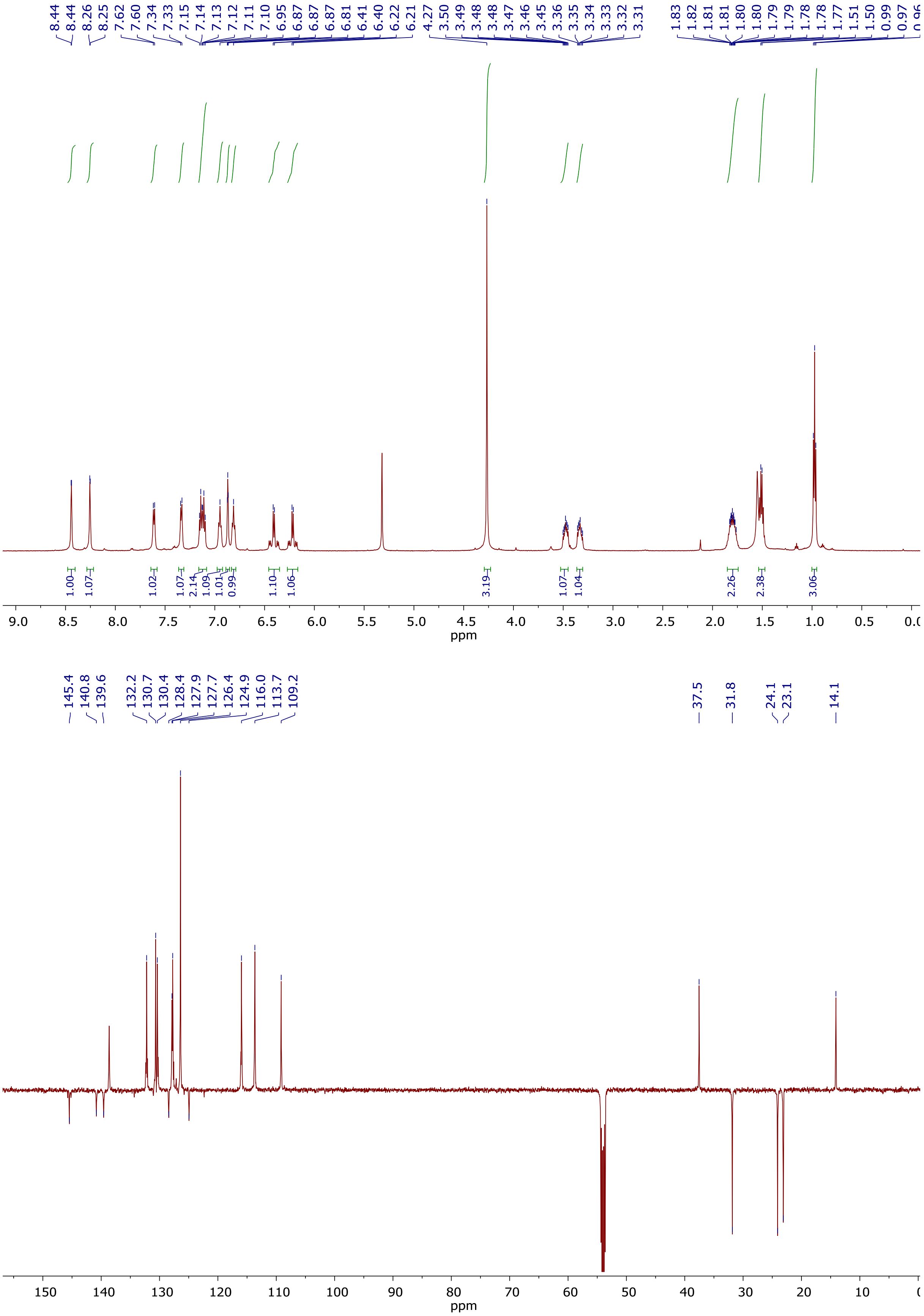

Figure S13. ^{1}H (top) and $^{13}C\{^{1}H\}$ APT (bottom) NMR spectra of complex **3a** (CD_2Cl_2, 600 and 151 MHz, respectively).

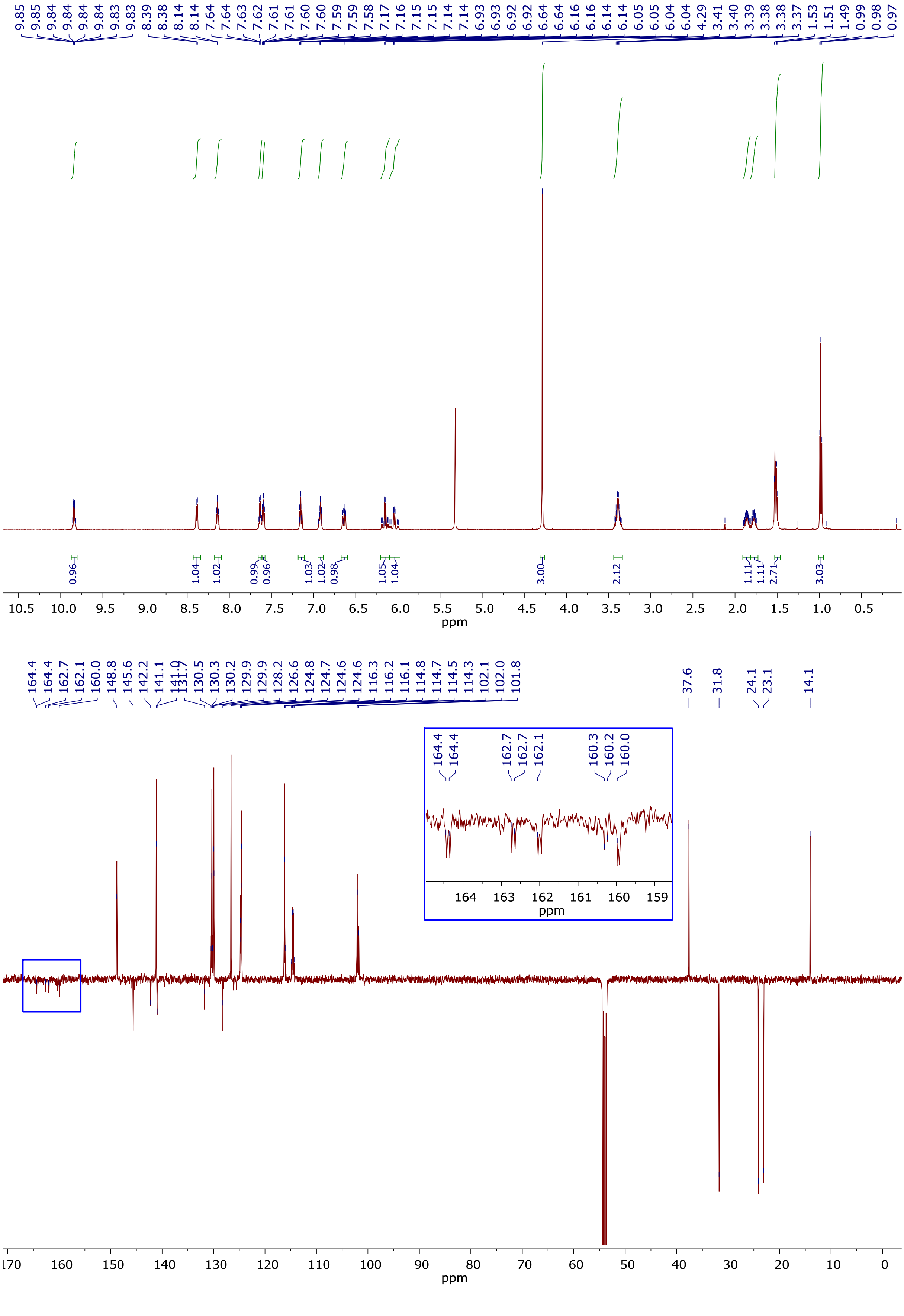

Figure S14. ^{1}H (top) and $^{13}C\{^{1}H\}$ APT (bottom) NMR spectra of complex **3b** (CD_2Cl_2, 600 and 151 MHz, respectively).

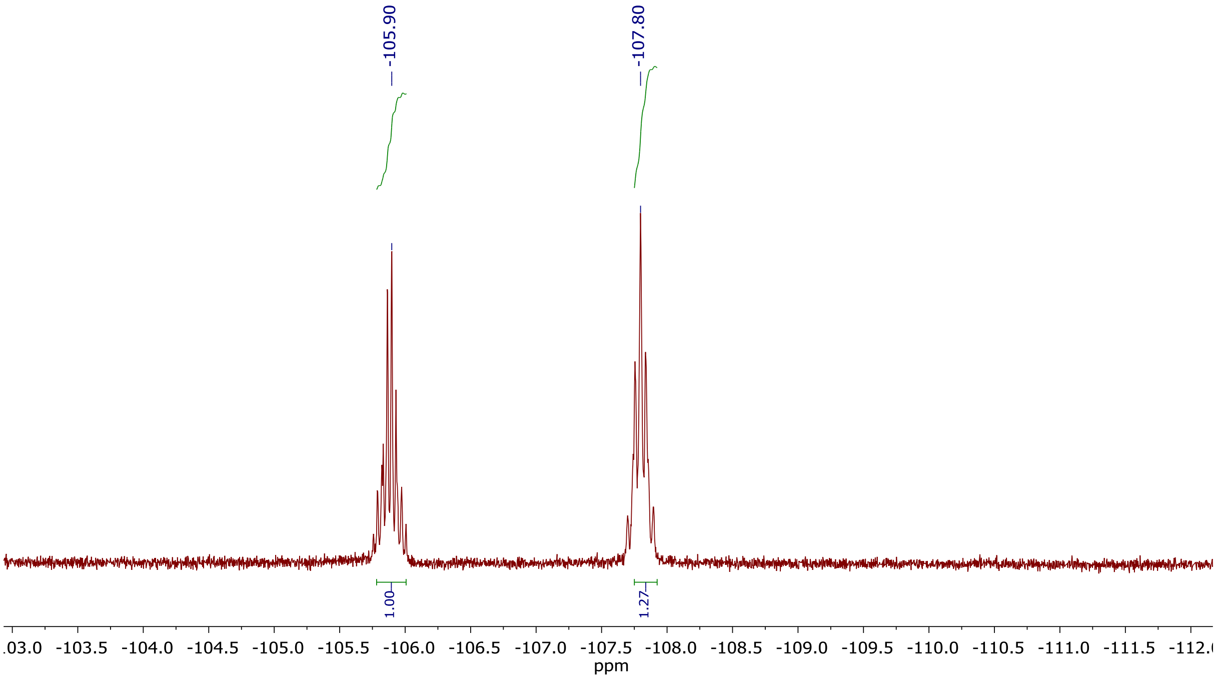

Figure S15. ^{19}F NMR spectrum of complex **3b** (CD_2Cl_2, 282 MHz).

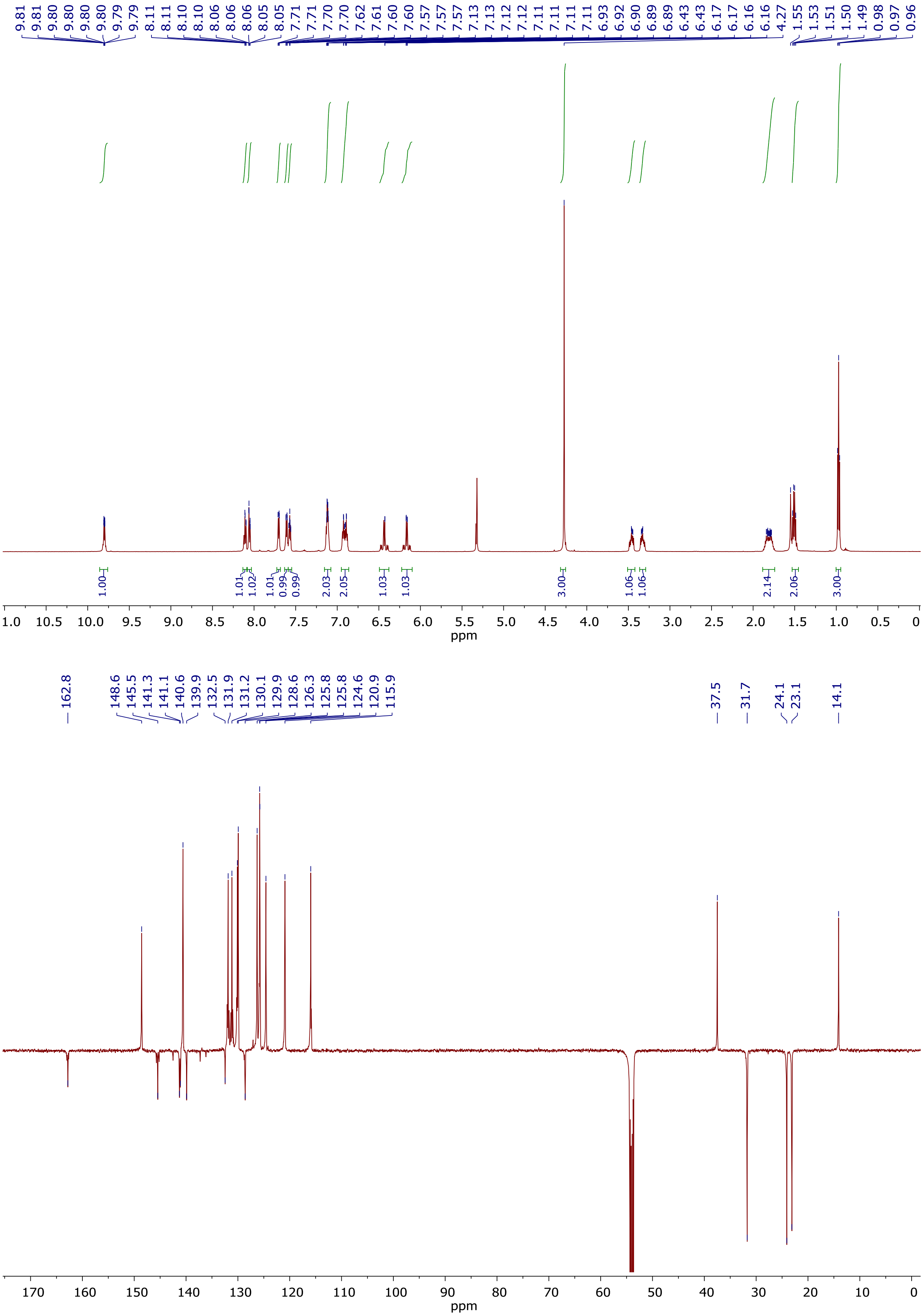

Figure S16. ^{1}H (top) and $^{13}C\{^{1}H\}$ APT (bottom) NMR spectra of complex **3c** (CD_2Cl_2, 600 and 151 MHz, respectively).

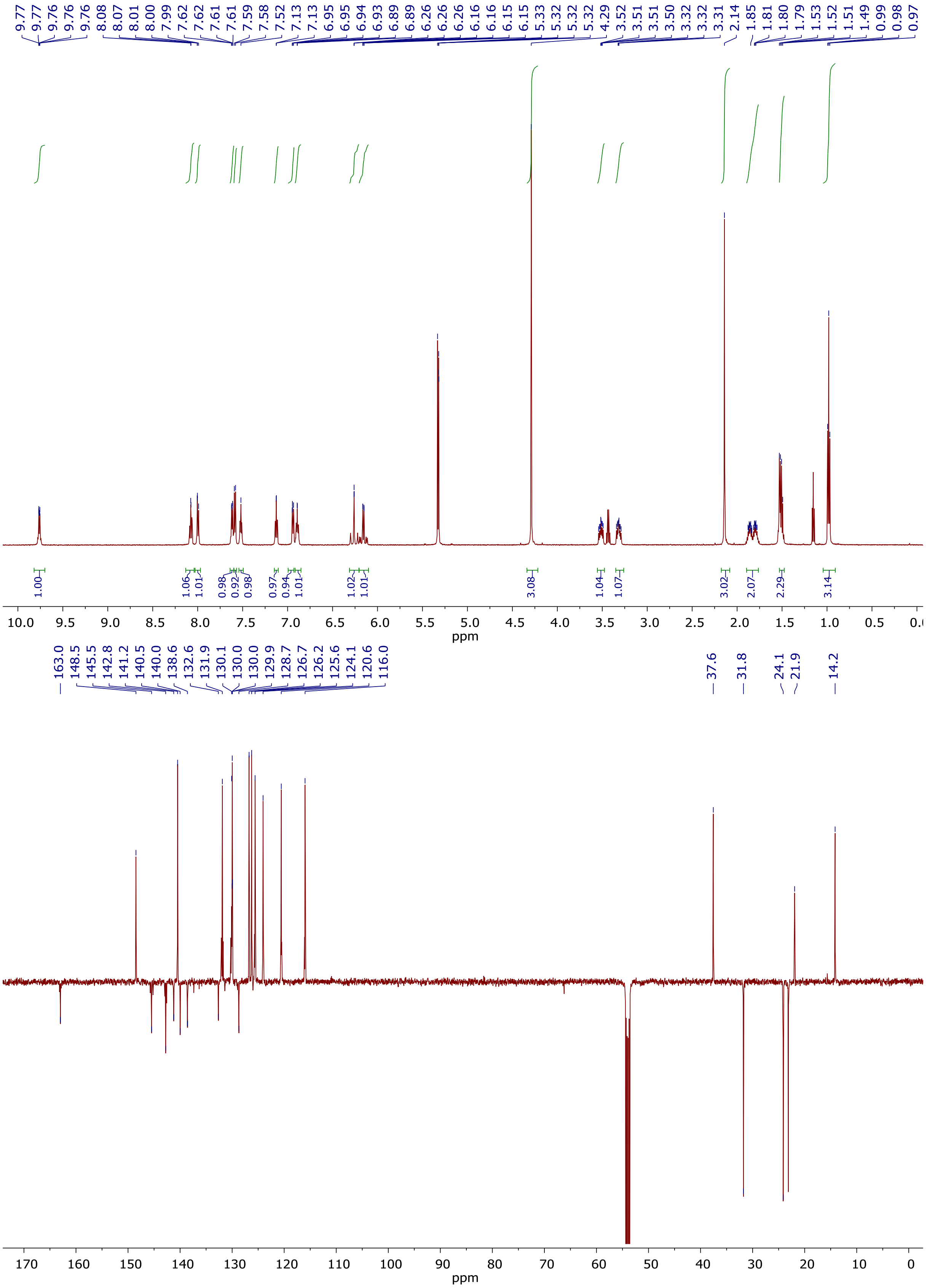

Figure S17. ^{1}H (top) and $^{13}C\{^{1}H\}$ APT (bottom) NMR spectra of complex **3d** (CD_2Cl_2, 600 and 151 MHz, respectively).

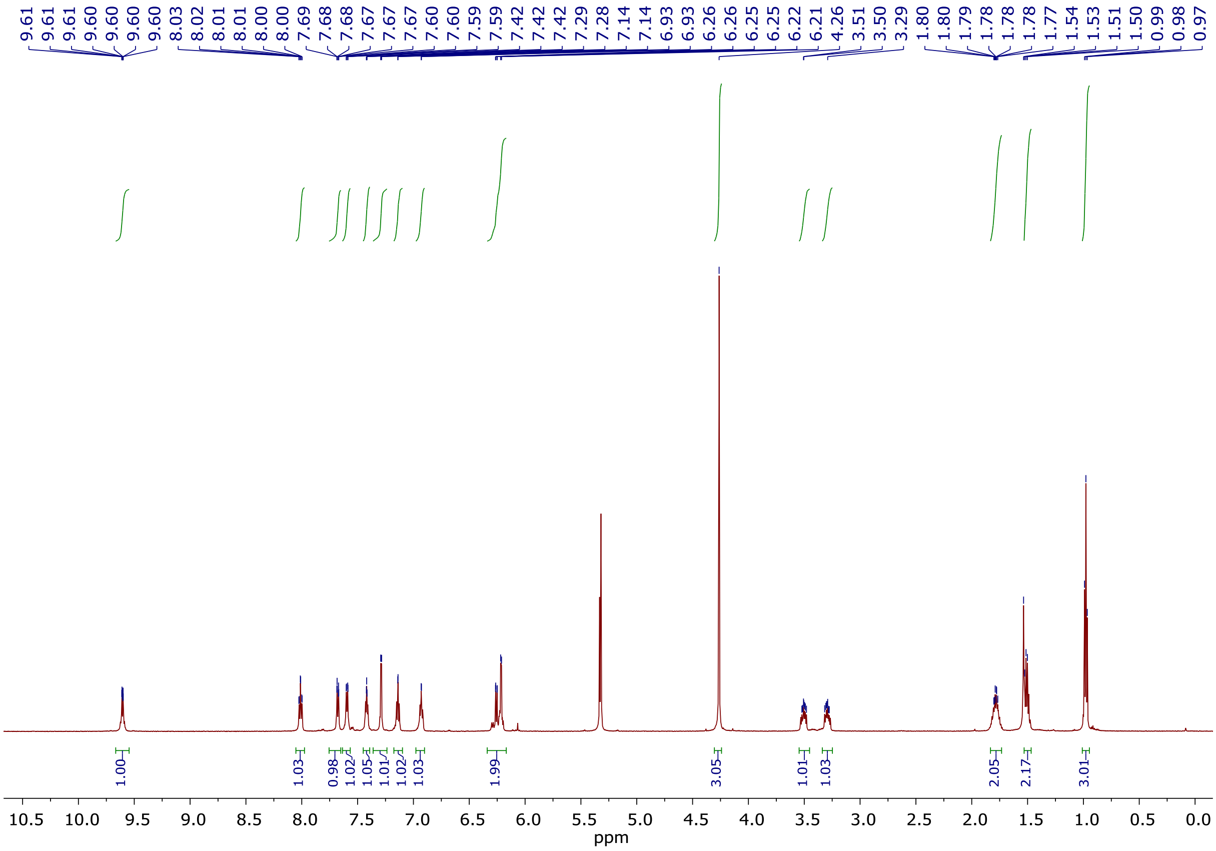

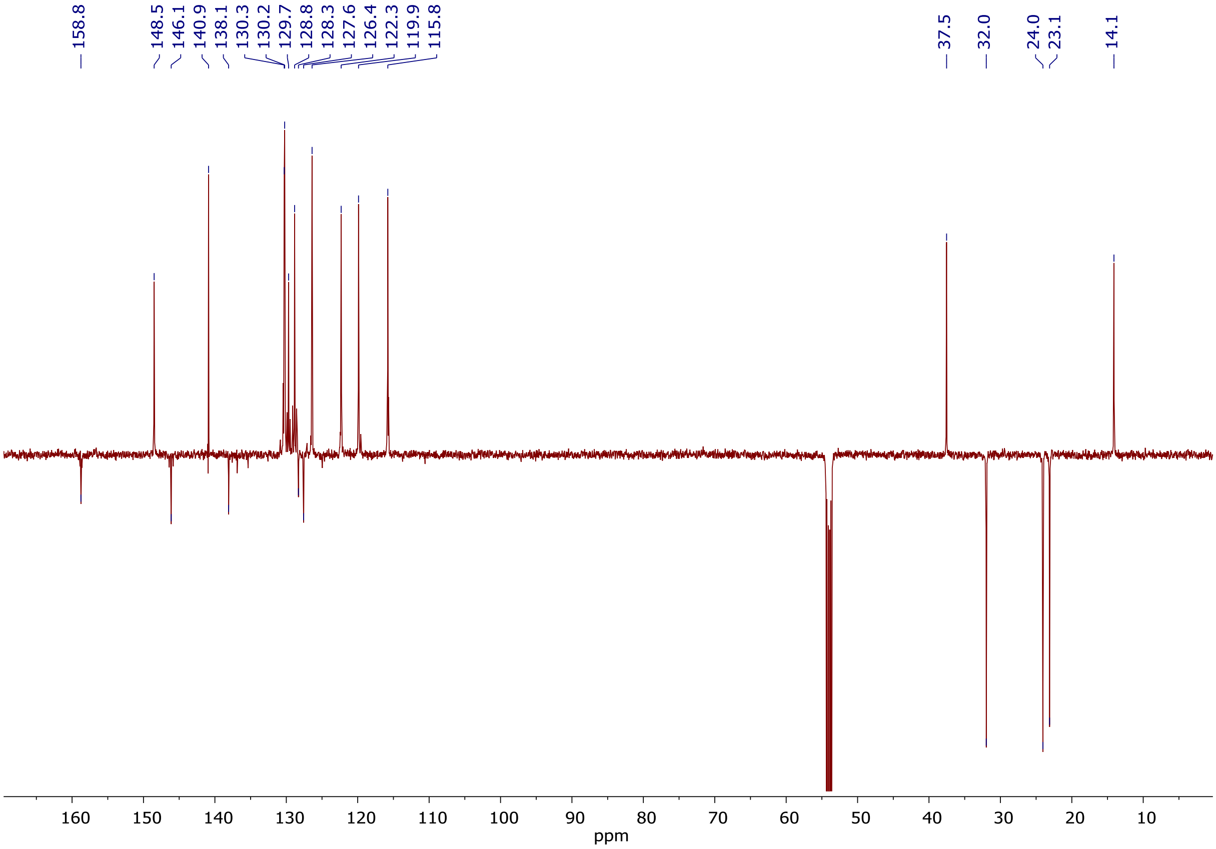

Figure S18. ^{1}H (top) and $^{13}C\{^{1}H\}$ APT (bottom) NMR spectra of complex **3e** (CD_2Cl_2, 600 and 151 MHz, respectively).

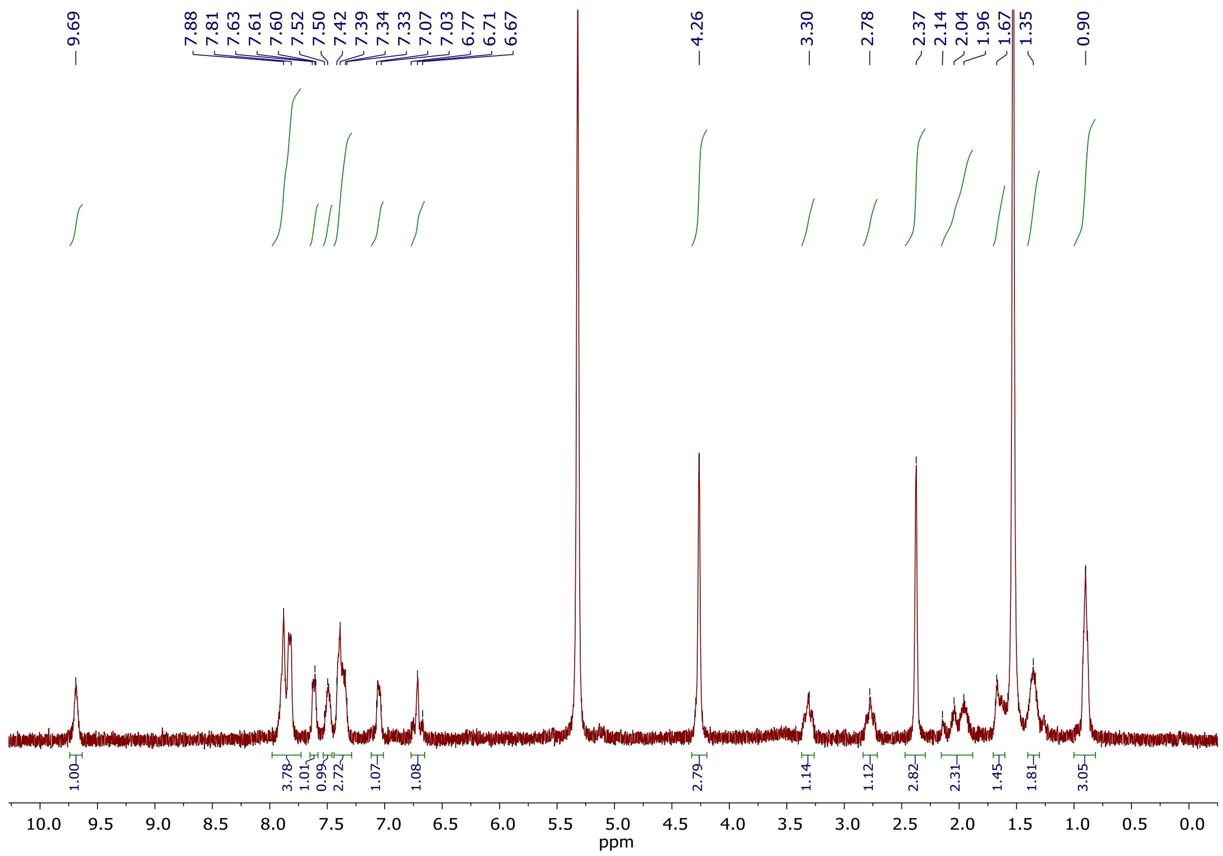

Figure S19. ^{1}H NMR spectrum of complex **4d** (CD_2Cl_2, 400 MHz).

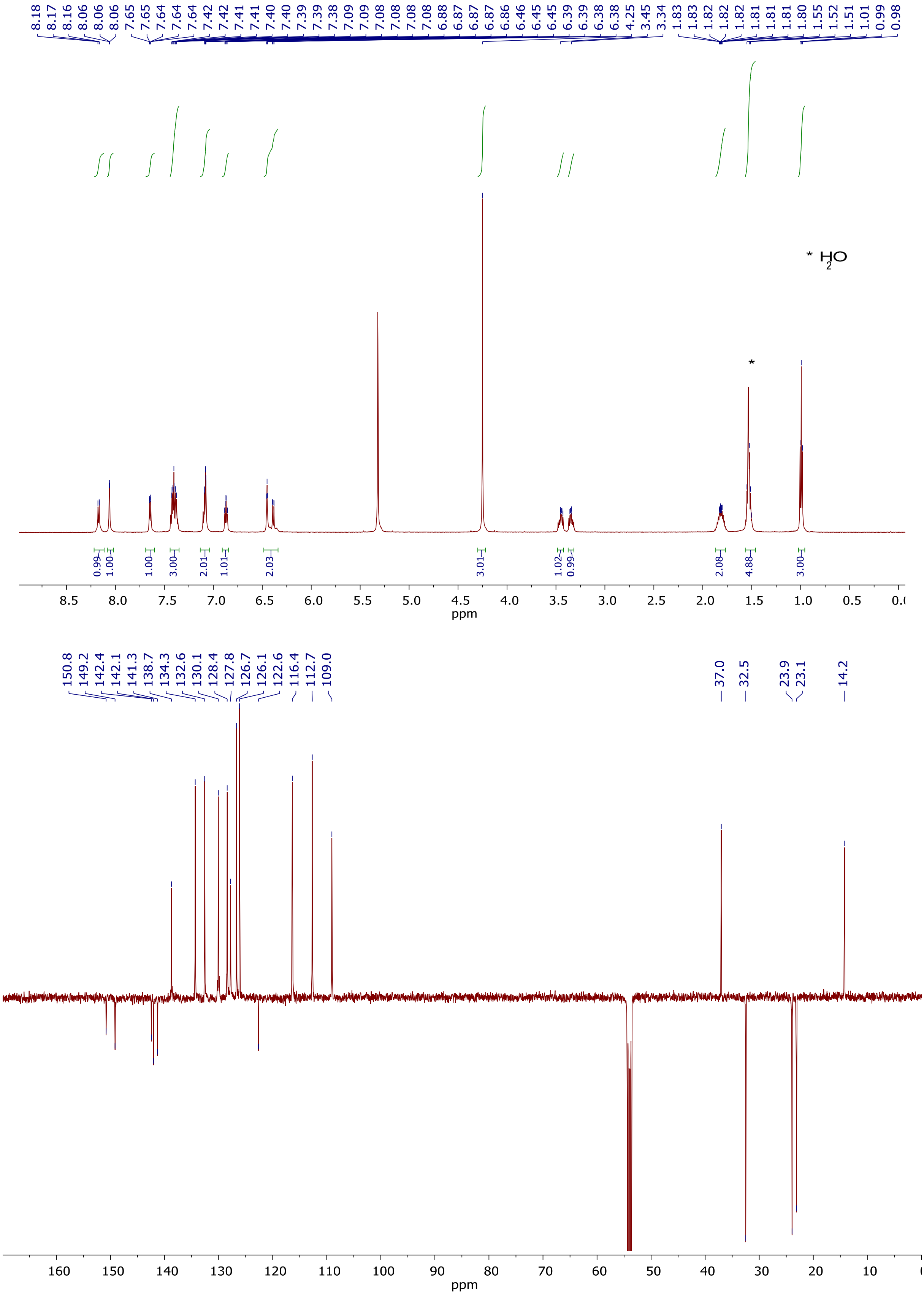

Figure S20. ^{1}H (top) and ^{13}C{^{1}H} APT (bottom) NMR spectra of complex **5a** (CD_2Cl_2, 600 and 151 MHz, respectively).

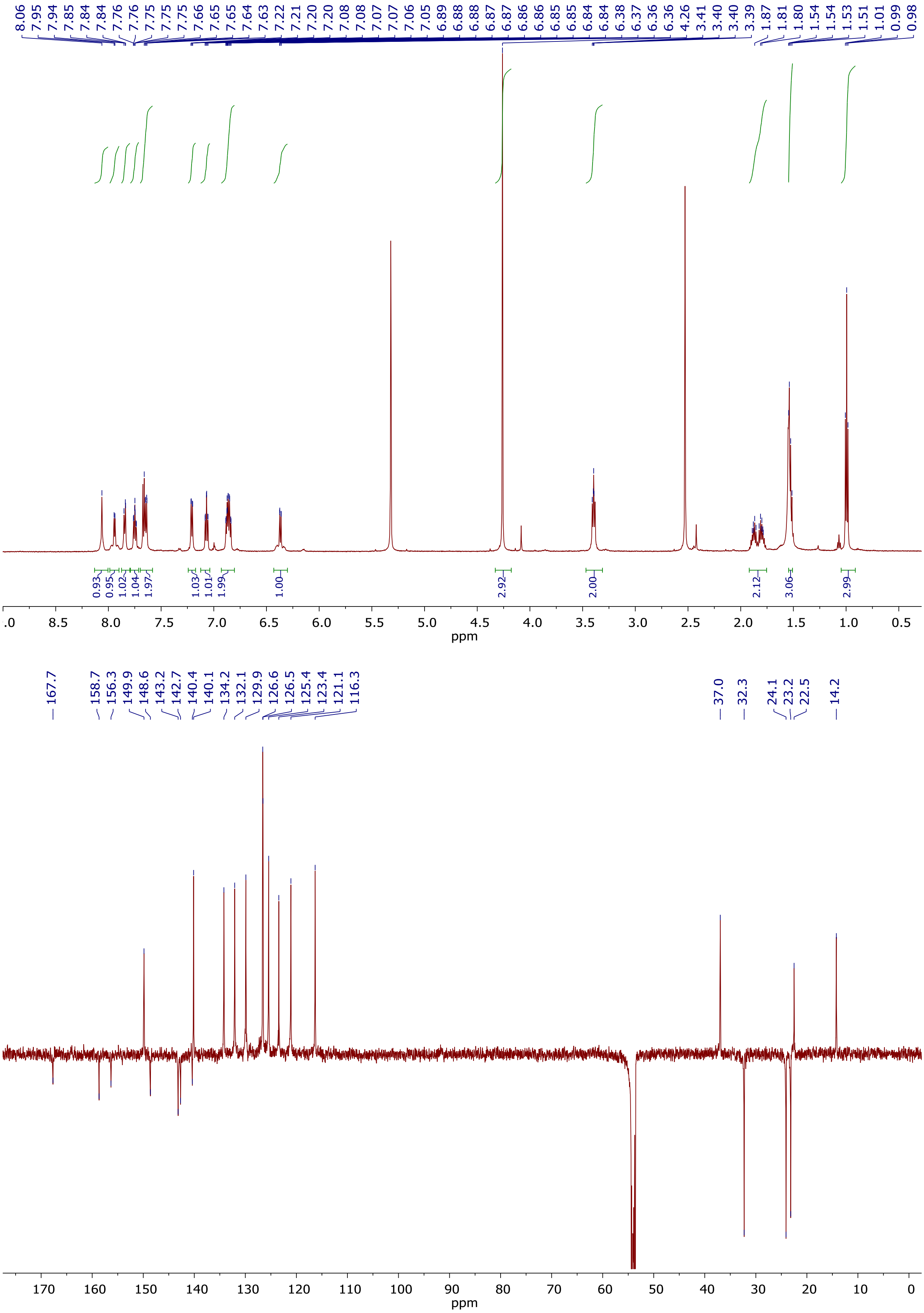

Figure S21. ^{1}H (top) and $^{13}C\{^{1}H\}$ APT (bottom) NMR spectra of complex **5d** (CD_2Cl_2, 600 and 151 MHz, respectively).

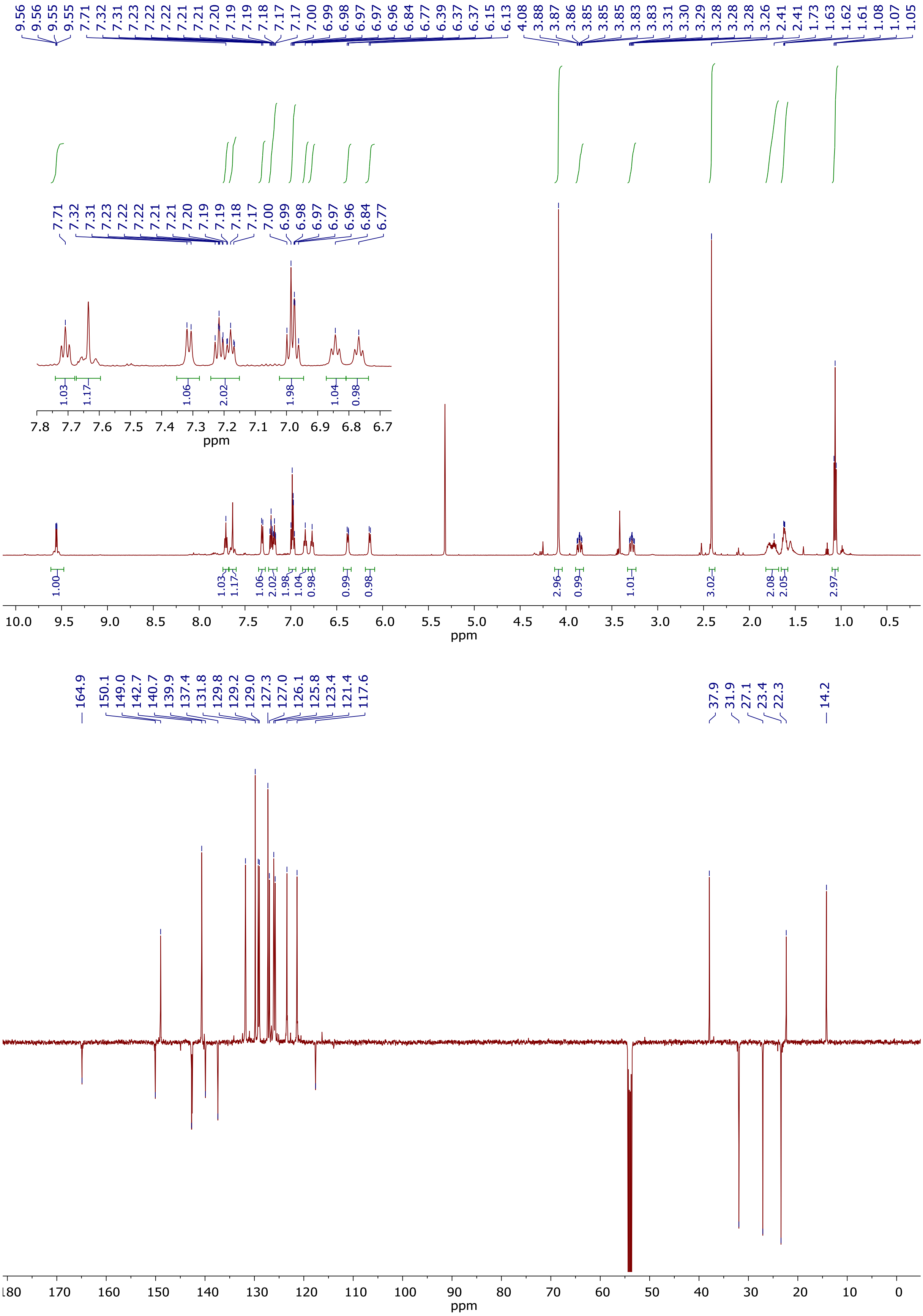

Figure S22. ^{1}H (top) and $^{13}C\{^{1}H\}$ APT (bottom) NMR spectra of complex **6d** (CD_2Cl_2, 600 and 151 MHz, respectively).

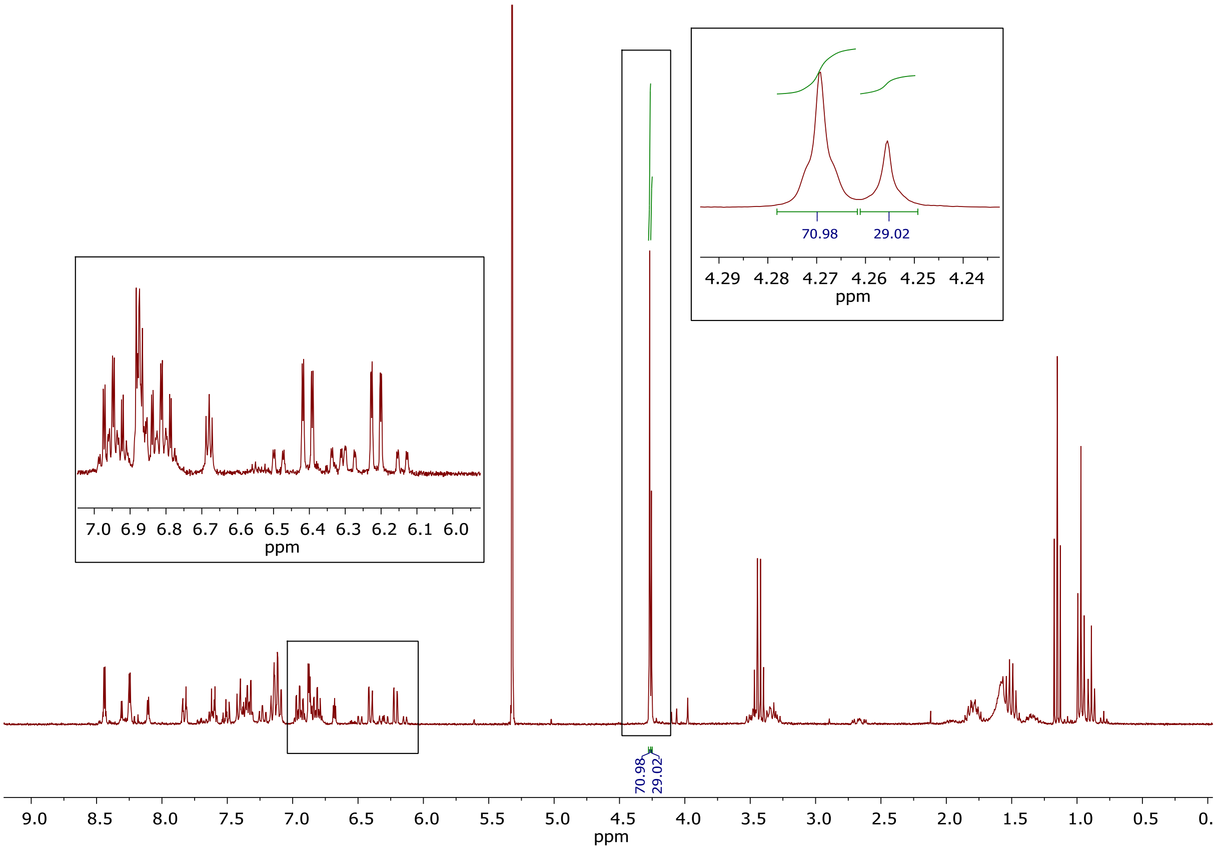

Figure S23. ^{1}H NMR spectrum after oxidation of *cis-C,C**-**2a**, complexes **3a** and **4a** in 70:30 ratio (CD_2Cl_2, 300 MHz).

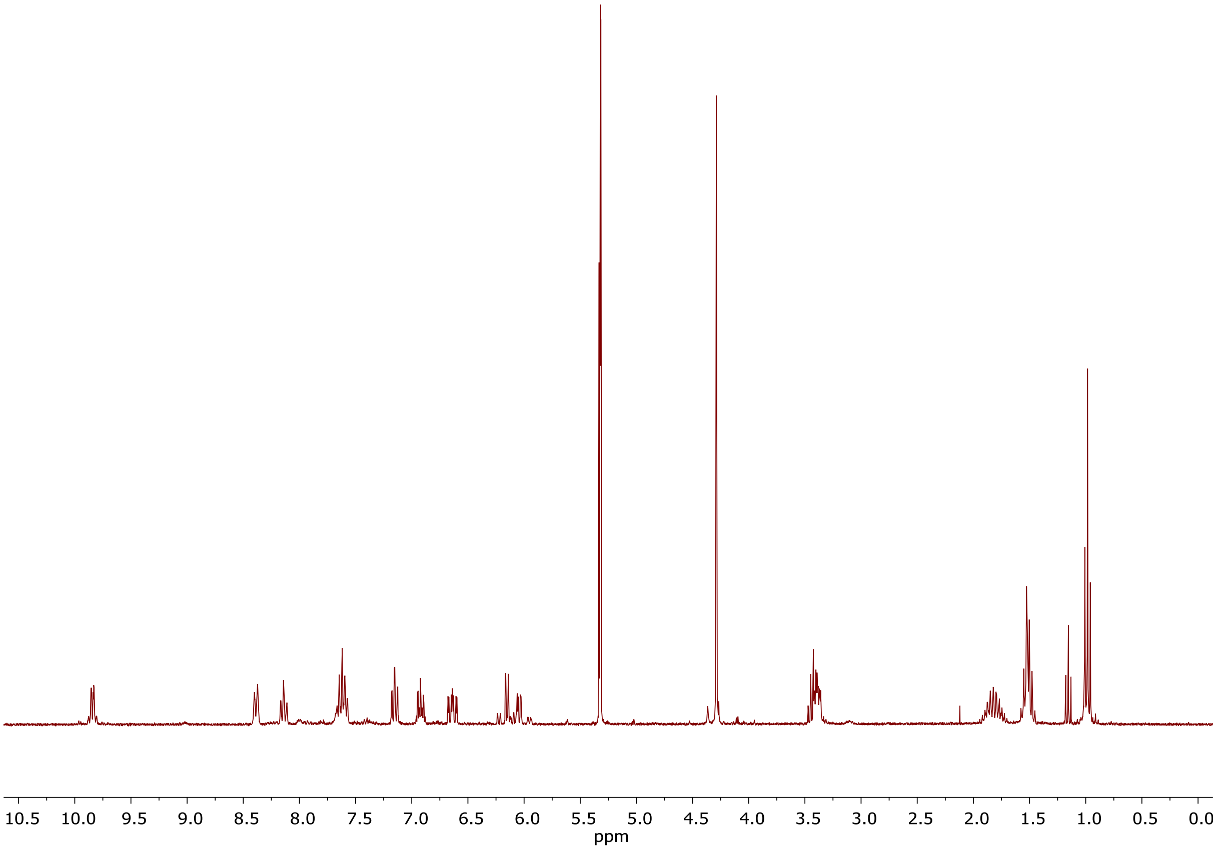

Figure S24. ^{1}H NMR spectrum after oxidation of *cis-C,C**-**2b** (CD_2Cl_2, 300 MHz).

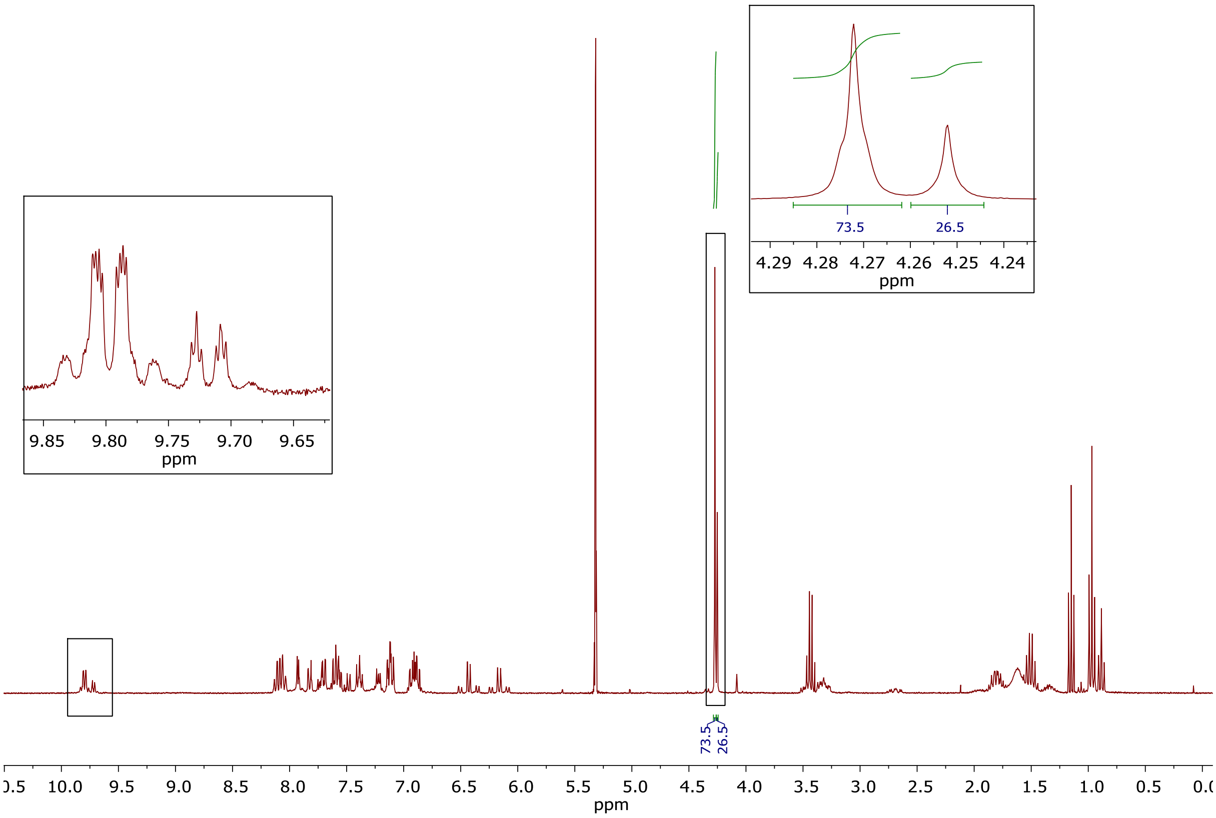

Figure S25. ^{1}H NMR spectrum after oxidation of *cis*-*C,C**-**2c**, complexes **3c** and **4c** in 74:26 ratio (CD_2Cl_2, 300 MHz).

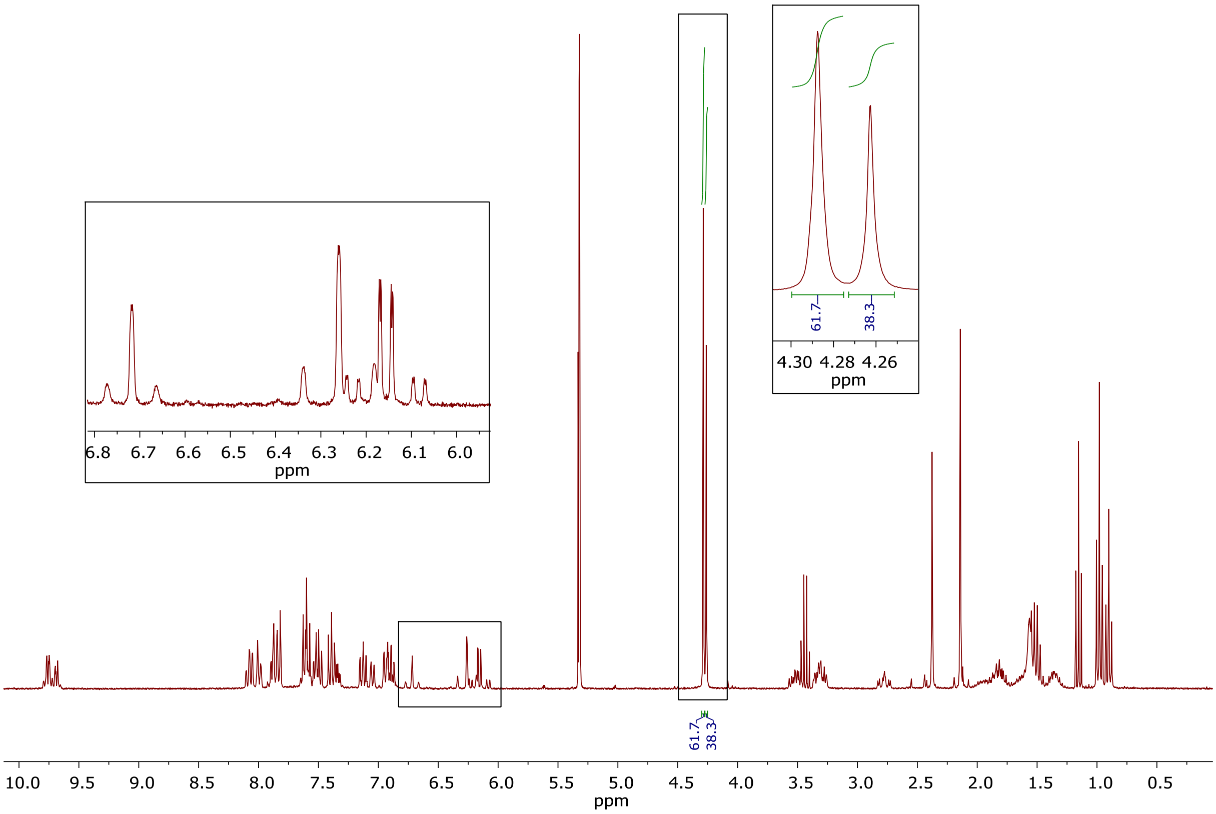

Figure S26. ^{1}H NMR spectrum after oxidation of *cis*-*C,C**-**2d**, complexes **3d** and **4d** in 62:38 ratio (CD_2Cl_2, 300 MHz).

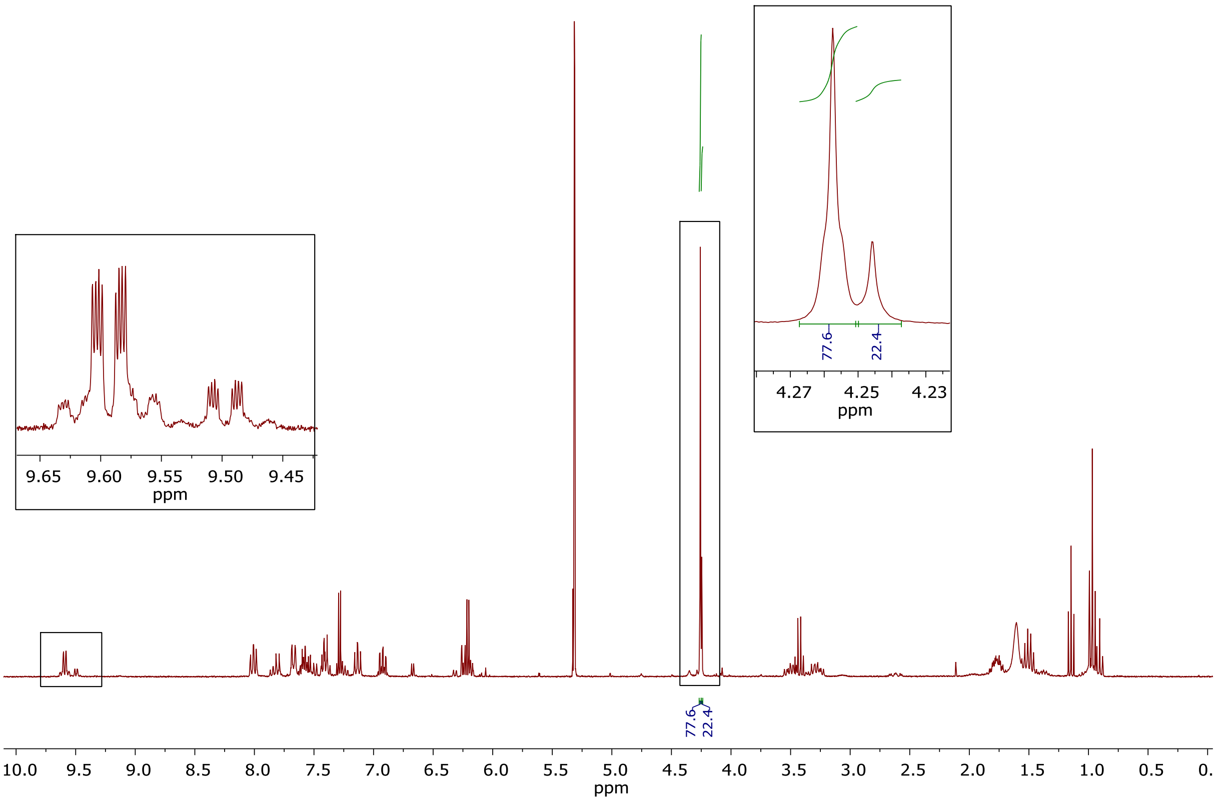

Figure S27. ^{1}H NMR spectrum after oxidation of *cis-C,C**-**2e**, complexes **3e** and **4e** in 78:22 ratio (CD_2Cl_2, 300 MHz).

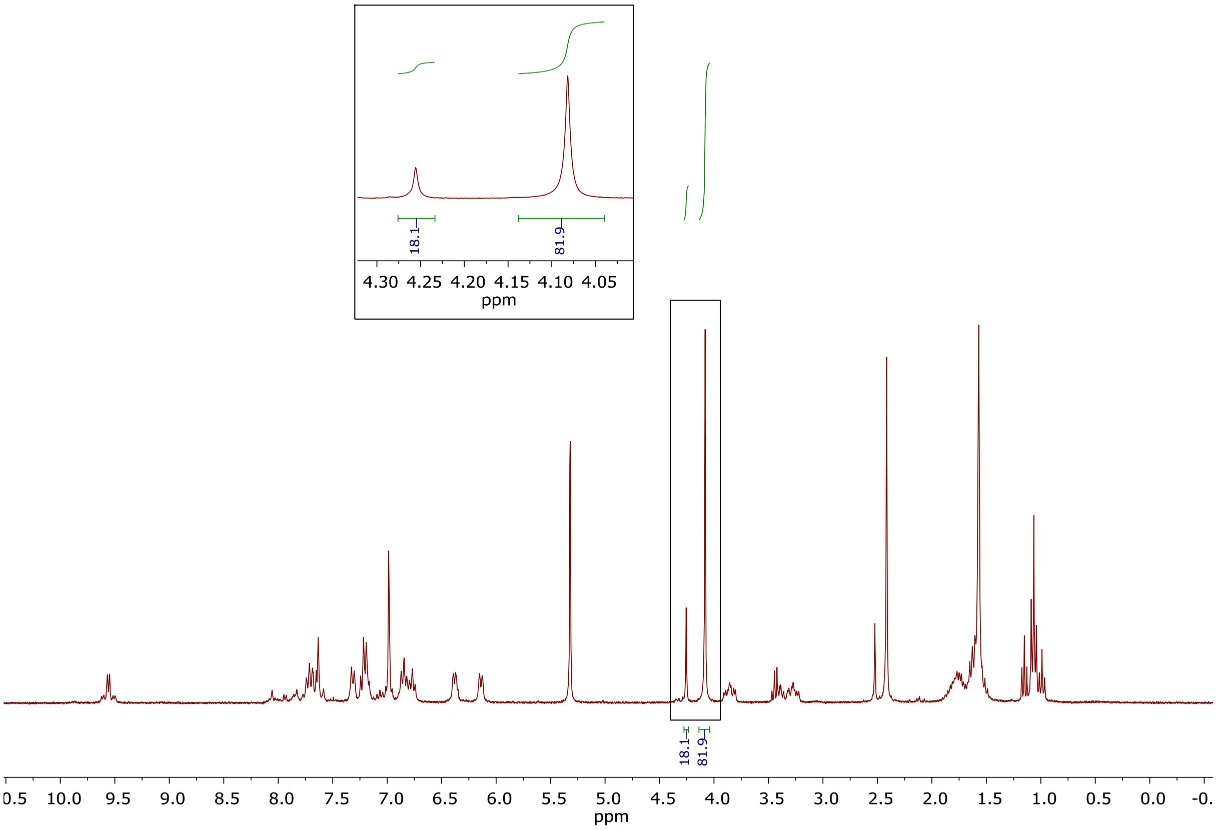

Figure S28. ^{1}H NMR spectrum after oxidation of *trans-C,C**-**2d**, complexes **5d** and **6d** in 18:82 ratio (CD_2Cl_2, 300 MHz).

Table S2. Molar ratios of complexes **3** and **4** resulting from the oxidative addition of $PhICl_2$ to *cis-C,C**-**2** as determined by ^{1}H NMR.

C^N ligand	Molar ratio **3**:**4**
ppz	70:30
dfppy	95:5
ppy	74:26
tpy	62:38
thpy	78:22

5. Excitation and emission spectra

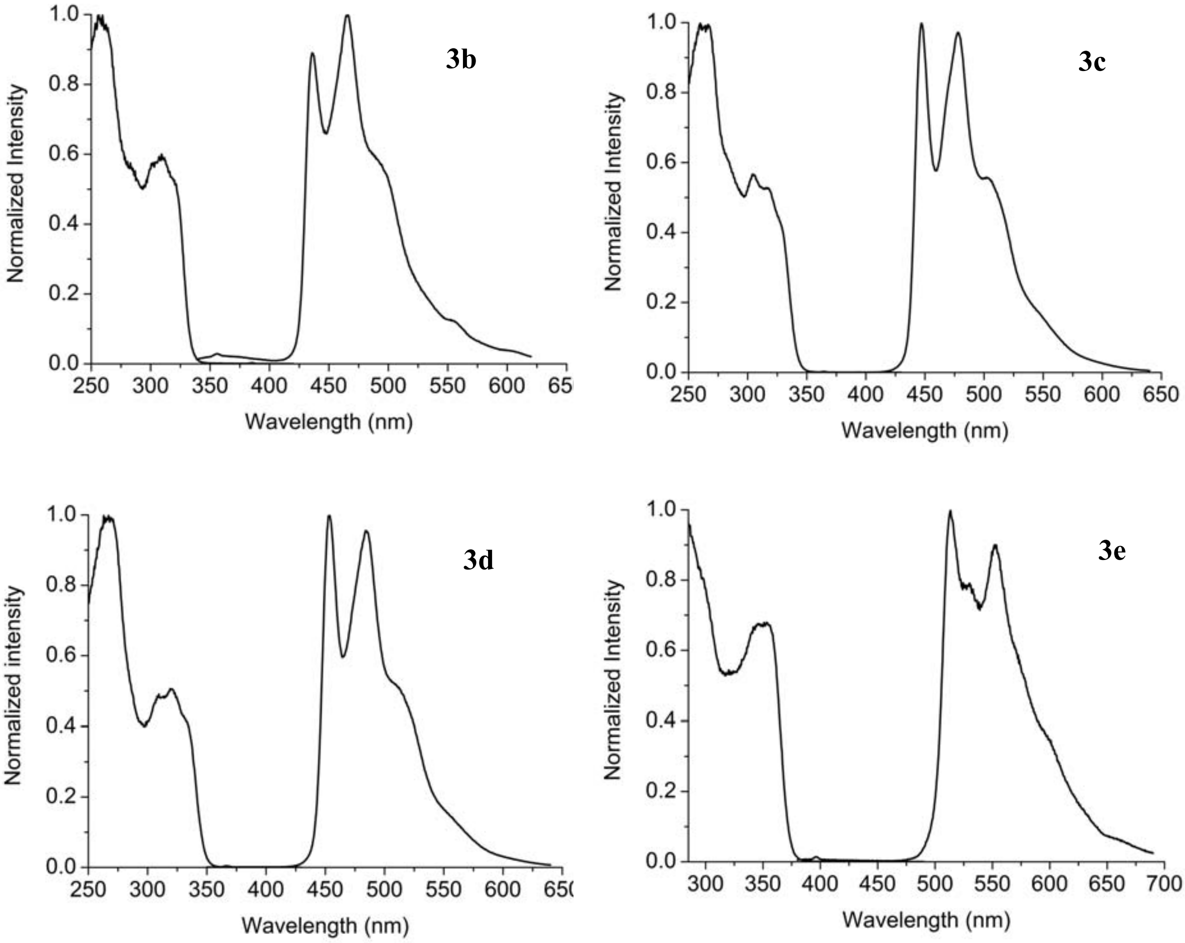

Figure S29. Excitation and emission spectra of complexes **3b-e** in CH_2Cl_2 at 298 K.

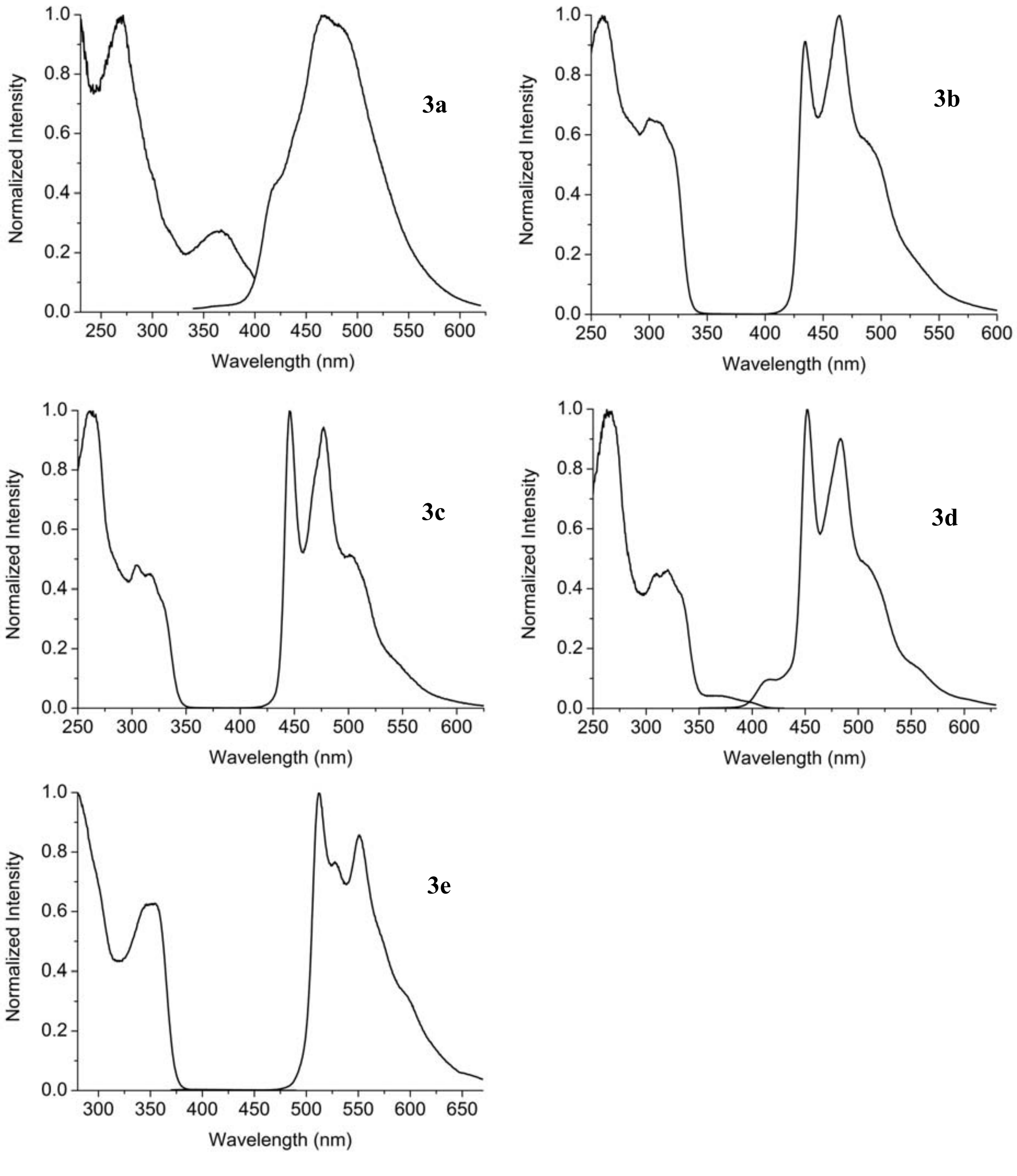

Figure S30. Excitation and emission spectra of complexes **3a-e** in PMMA matrices (2 wt%) at 298 K.

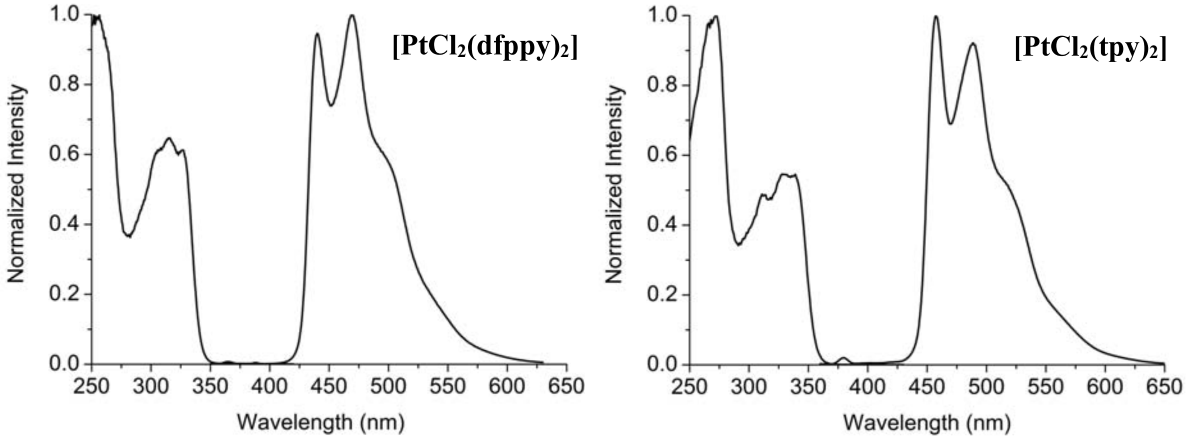

Figure S31. Excitation and emission spectra of complexes (*OC*-6-33)-[$PtCl_2$(dfppy)$_2$] and (*OC*-6-33)-[$PtCl_2$(tpy)$_2$] in CH_2Cl_2 at 298 K.

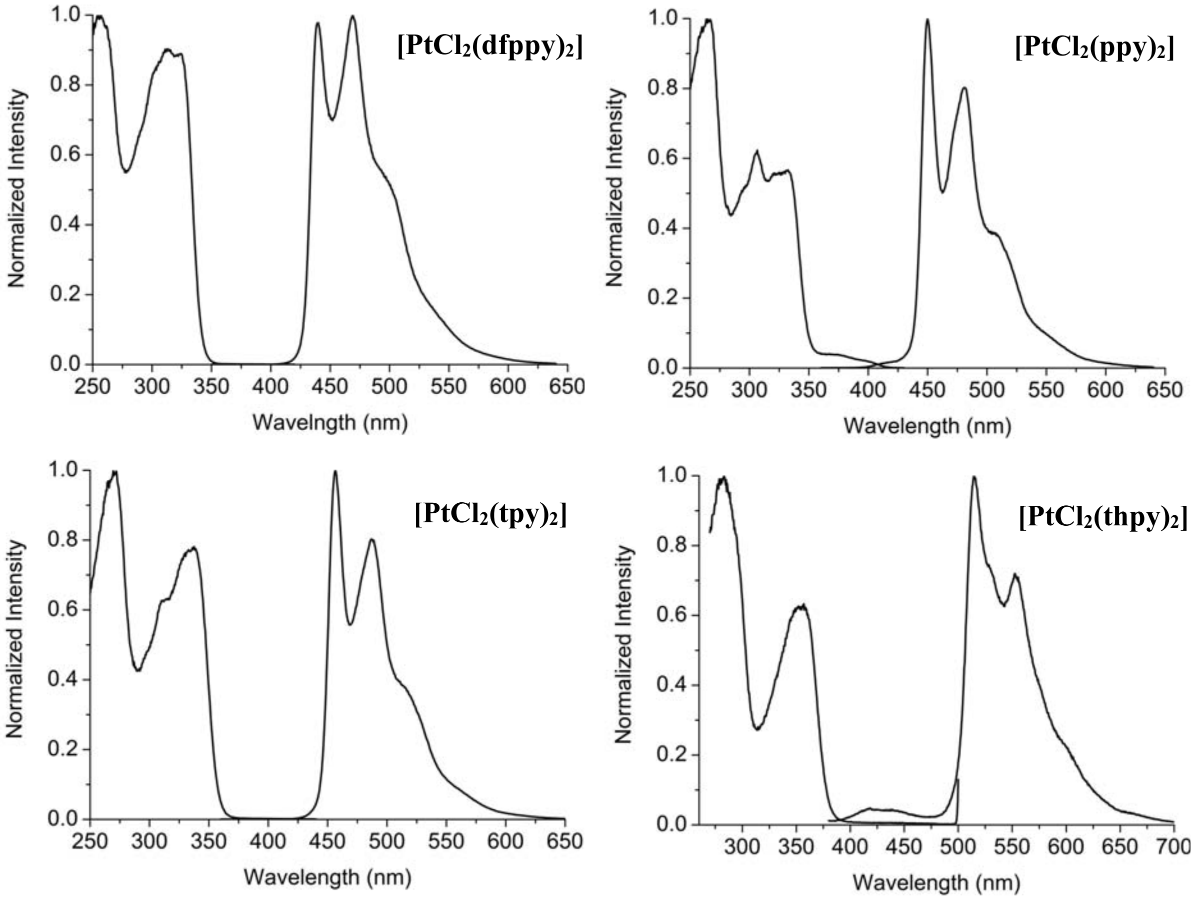

Figure S32. Excitation and emission spectra of complexes (*OC*-6-33)-[$PtCl_2$(C^N)$_2$] (C^N = dfppy, ppy, tpy, thpy) in PMMA matrices (2 wt%) at 298 K.

6. Computational methods

DFT calculations were performed with Gaussian 09,[5] using the B3LYP functional[6,7] together with the 6-31G**[8,9] basis set for the light atoms and the LANL2DZ[10] basis set and effective core potential for the Pt atom. Geometry optimizations were carried out without symmetry restrictions, using "tight" convergence criteria and "ultrafine" integration grid. Vertical excitation energies were obtained from TDDFT calculations at the ground-state geometries. Triplet-state geometry optimizations were performed following a published strategy.[11] The solvent effect (CH_2Cl_2) was accounted for in all cases by using the integral equation formalism variant of the polarizable continuum solvation model (IEFPCM).[12] The optimized geometries were confirmed as minima on the potential energy surface by performing frequency calculations (zero imaginary frequencies). Natural spin densities were obtained from natural population analyses using the NBO 5.9 program.[13]

7. Computational data

7.1. Complex 3d

Table S3. Fragment contributions (%; from atomic orbital contributions) to the frontier orbitals of **3d** in CH_2Cl_2 solution.

energy (a.u.)	number	L1	L2	L3	L4	Pt
–0.010	134 (LUMO+5)	12	83	0	0	5
–0.037	133 (LUMO+4)	19	36	2	5	39
–0.040	132 (LUMO+3)	64	14	4	2	16
–0.044	131 (LUMO+2)	62	15	4	4	15
–0.066	130 (LUMO+1)	83	12	0	2	4
–0.069	129 (LUMO)	11	84	1	0	4
–0.231	128 (HOMO)	92	0	3	0	4
–0.243	127 (HOMO–1)	0	51	5	35	8
–0.247	126 (HOMO–2)	59	4	26	2	9
–0.251	125 (HOMO–3)	5	14	14	59	7
–0.252	124 (HOMO–4)	14	25	13	45	4
–0.254	123 (HOMO–5)	22	7	38	30	3

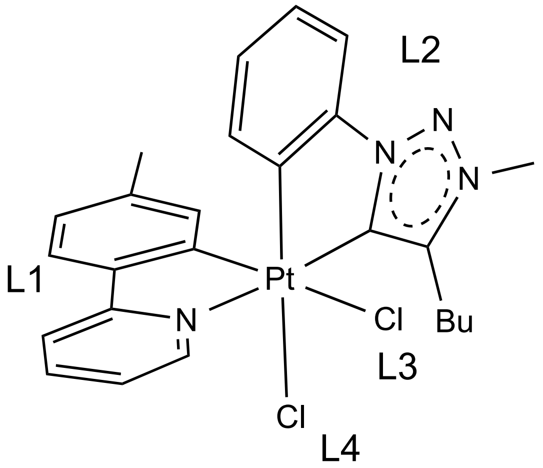

Figure S33. Ligand numbering in complex **3d**.

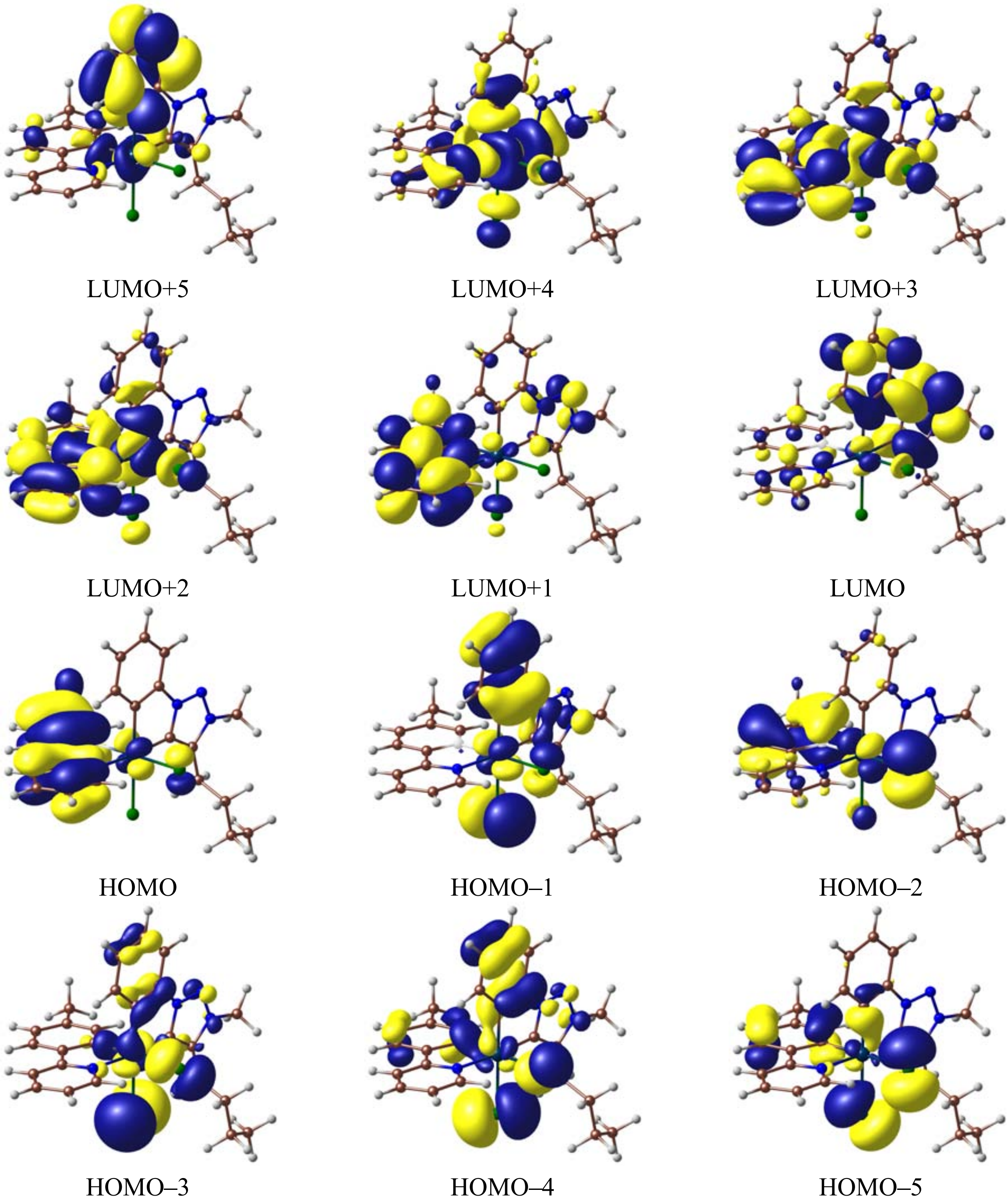

Figure S34. Molecular orbital isosurfaces of **3d** (0.03 e bohr^{-3}).

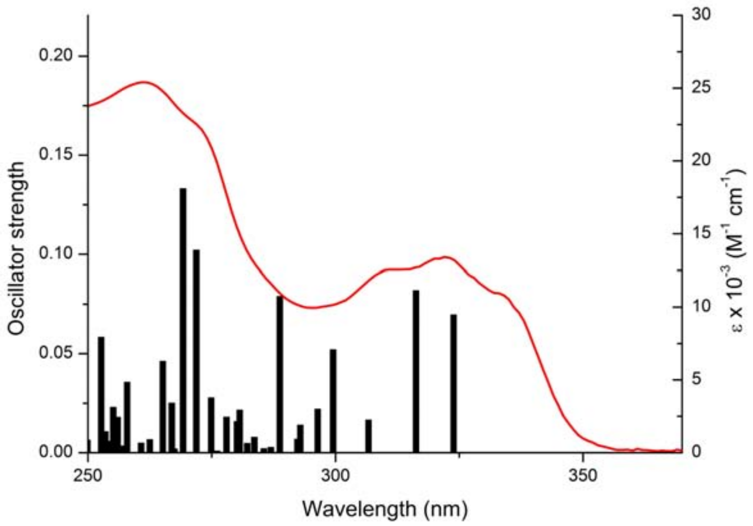

Figure S35. Calculated stick absorption spectrum of **3d** compared with the experimental spectrum in CH_2Cl_2 solution (*ca.* 1×10^{-5} M) at 298 K.

Table S4. Selected vertical singlet excitations of **3d** from TDDFT calculations at the ground state geometry in CH_2Cl_2 solution.

State	monoexcitations	AE/eV	λ/nm	Oscillator strength
S1	126 ->129 (4%)<br>128 ->129 (72%)<br>128 ->130 (10%)<br>128 ->131 (5%)	3.829	323.9	0.0697
S2	128 ->129 (16%)<br>128 ->130 (78%)	3.920	316.3	0.0819
S3	127 ->129 (67%)<br>127 ->130 (10%)<br>127 ->131 (4%)<br>128 ->129(4%)<br>128 ->130 (3%)	4.043	306.7	0.0166
S4	127 ->129 (18%)<br>127 ->130 (69%)	4.140	299.5	0.0521
S5	123 ->129 (6%)<br>125 ->130 (2%)<br>126 ->129 (57%)<br>126 ->131 (5%)<br>126 ->132 (3%)<br>127 ->129 (4%)<br>127 ->130 (3%)<br>128 ->129 (6%)<br>128 ->131 (4%)<br>128 ->132 (5%)	4.183	296.4	0.0221
S6	124 ->129 (7%)<br>124 ->130 (8%)<br>125 ->129 (18%)<br>125 ->130 (18%)<br>126 ->130 (23%)<br>128 ->131 (12%)<br>128 ->132 (2%)	4.233	292.9	0.014
S7	123 ->130 (4%)<br>124 ->129 (3%)	4.242	292.3	0.0069

	125 ->129 (11%)<br>125 ->130 (12%)<br>126 ->129 (16%)<br>126 ->130 (4%)<br>128 ->131 (30%)<br>128 ->132 (8%)			
S8	123 ->129 (8%)<br>125 ->130 (5%)<br>126 ->129 (5%)<br>126 ->130 (51%)<br>128 ->130 (2%)<br>128 ->132 (12%)	4.294	288.8	0.079
S9	122 ->129 (39%)<br>122 ->131 (2%)<br>124 ->130 (6%)<br>125 ->129 (34%)<br>125 ->130 (9%)	4.321	286.9	0.0028
S10	123 ->129 (13%)<br>124 ->129 (51%)<br>125 ->130 (15%)<br>126 ->129 (4%)<br>126 ->130 (3%)<br>128 ->133 (4%)	4.342	285.5	0.0021
S11	121 ->129 (3%)<br>122 ->129 (39%)<br>123 ->129 (9%)<br>124 ->129 (3%)<br>124 ->130 (14%)<br>125 ->129 (19%)<br>126 ->129 (2%)<br>127 ->130 (2%)	4.372	283.6	0.0079
S12	122 ->129 (8%)<br>123 ->129 (36%)<br>124 ->130 (10%)<br>125 ->129 (7%)<br>125 ->130 (26%)<br>126 ->129 (2%)<br>128 ->133 (3%)	4.394	282.2	0.0048
S13	122 ->130 (2%)<br>123 ->129 (21%)<br>123 ->130 (12%)<br>124 ->129 (19%)<br>124 ->130 (29%)<br>128 ->133 (3%)	4.418	280.7	0.0216
S14	123 ->130 (20%)<br>125 ->130 (4%)<br>126 ->133 (4%)<br>127 ->130 (4%)<br>127 ->133 (5%)<br>128 ->133 (50%)	4.426	280.1	0.0158

Table S5. Lowest-energy vertical triplet excitations of **3d** from TDDFT calculations at the ground state geometry in CH_2Cl_2 solution.

**State**	**monoexcitations**	**AE/eV**	**λ/nm**
T1	117 ->137 (2%)<br>126 ->130 (3%)<br>128 ->129 (10%)<br>128 ->130 (66%)<br>128 ->131 (6%)	2.903	427.1
T2	120 ->129 (6%)<br>121 ->129 (11%)<br>124 ->129 (14%)<br>125 ->129 (7%)<br>126 ->129 (3%)<br>127 ->129 (31%)<br>127 ->130 (3%)<br>127 ->134 (3%)	3.193	388.3
T3	118 ->129 (4%)<br>120 ->129 (60%)<br>120 ->130 (7%)<br>121 ->129 (8%)<br>125 ->129 (3%)<br>127 ->129 (4%)	3.481	356.2
T4	117 ->131 (4%)<br>117 ->132 (5%)<br>126 ->129 (3%)<br>126 ->130 (7%)<br>126 ->132 (4%)<br>128 ->129 (2%)<br>128 ->130 (12%)<br>128 ->131 (37%)<br>128 ->132 (13%)	3.682	336.8

7.2. Complex (*OC*-6-33)-[$PtCl_2(tpy)_2$]

Table S6. Fragment contributions (%; from atomic orbital contributions) to the frontier orbitals of (*OC*-6-33)-[$PtCl_2(tpy)_2$] in CH_2Cl_2 solution.

energy (a.u.)	number	L1	L2	L3	L4	Pt
–0.040	121 (LUMO+5)	31	31	6	6	27
–0.046	120 (LUMO+4)	49	49	0	0	1
–0.051	119 (LUMO+3)	47	47	0	0	3
–0.059	118 (LUMO+2)	28	27	2	2	41
–0.073	117 (LUMO+1)	44	48	1	0	6
–0.074	116 (LUMO)	48	44	1	2	5
–0.235	115 (HOMO)	48	43	1	1	7
–0.236	114 (HOMO–1)	43	48	4	4	0
–0.250	113 (HOMO–2)	20	21	28	29	0
–0.252	112 (HOMO–3)	33	34	10	9	13
–0.256	111 (HOMO–4)	13	12	35	35	4
–0.256	110 (HOMO–5)	28	27	21	21	3

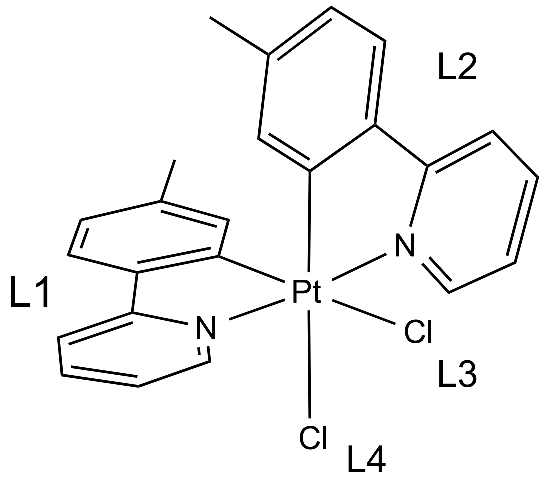

Figure S36. Ligand numbering in complex (*OC*-6-33)-[$PtCl_2(tpy)_2$].

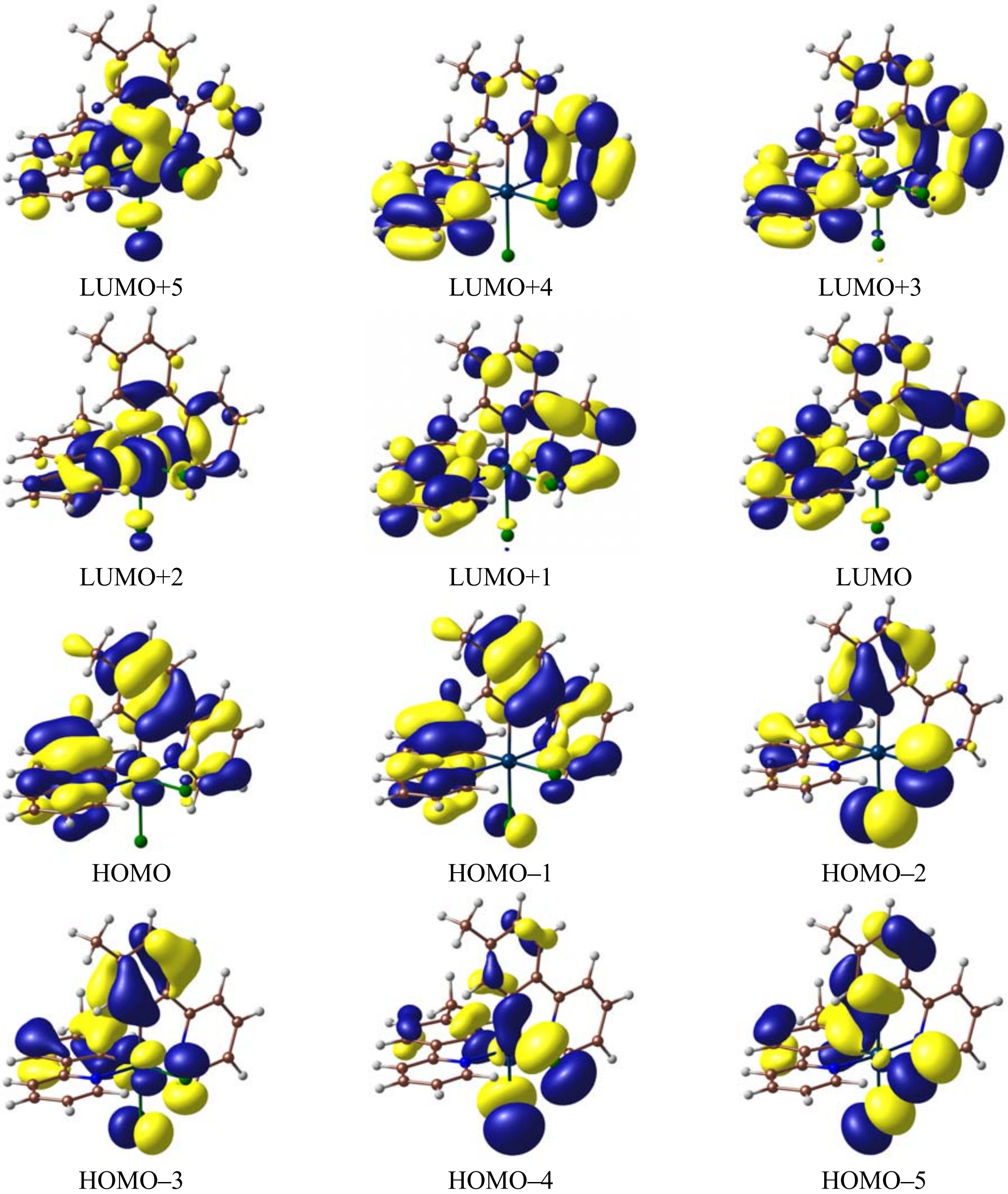

Figure S37. Molecular orbital isosurfaces of (*OC*-6-33)-[$PtCl_2(tpy)_2$] (0.03 e bohr^{-3}).

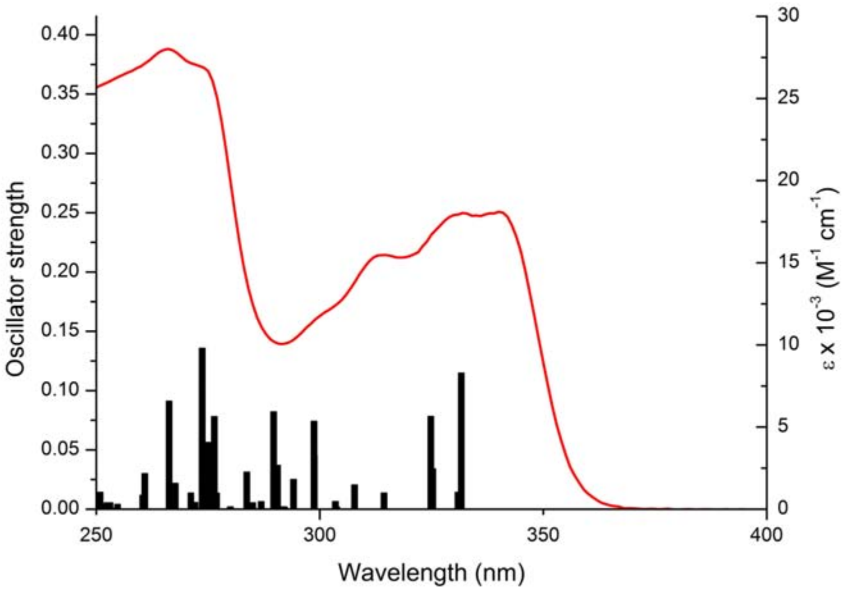

Figure S38. Calculated stick absorption spectrum of (*OC*-6-33)-[$PtCl_2(tpy)_2$] compared with the experimental spectrum in CH_2Cl_2 solution (*ca.* 1×10^{-5} M) at 298 K.

Table S7. Selected vertical singlet excitations of (*OC*-6-33)-[$PtCl_2(tpy)_2$] from TDDFT calculations at the ground state geometry in CH_2Cl_2 solution.

State	monoexcitations	AE/eV	λ/nm	Oscillator strength
S1	112 ->116 (6%)<br>115 ->116 (83%)<br>115 ->121 (2%)	3.738	331.7	0.1148
S2	112 ->117 (3%)<br>112 ->118 (3%)<br>115 ->117 (66%)<br>115 ->118 (25%)	3.747	330.9	0.0142
S3	113 ->116 (3%)<br>114 ->116 (84%)<br>114 ->117 (5%)	3.812	325.3	0.0341
S4	113 ->117 (2%)<br>114 ->116 (6%)<br>114 ->117 (75%)<br>114 ->118 (13%)	3.817	324.9	0.0782
S5	112 ->118 (6%)<br>114 ->116 (3%)<br>115 ->117 (27%)<br>115 ->118 (59%)	3.944	314.4	0.0136
S6	114 ->117 (14%)<br>114 ->118 (78%)<br>115 ->116 (3%)	4.028	307.8	0.0204
S7	110 ->117 (2%)<br>111 ->116 (8%)<br>112 ->116 (11%)<br>113 ->116 (10%)<br>113 ->117 (48%)<br>113 ->118 (7%)<br>115 ->116 (2%)	4.081	303.8	0.0017
S8	110 ->116 (4%)<br>111 ->117 (6%)	4.085	303.5	0.0065

	112 ->117 (10%)<br>113 ->116 (55%)<br>113 ->117 (9%)			
S9	111 ->117 (2%)<br>111 ->118 (2%)<br>112 ->116 (19%)<br>112 ->117 (36%)<br>112 ->118 (7%)<br>113 ->116 (15%)<br>113 ->117 (3%)<br>115 ->118 (2%)	4.150	298.8	0.0451
S10	112 ->116 (44%)<br>112 ->117 (15%)<br>112 ->118 (3%)<br>113 ->116 (6%)<br>113 ->117 (10%)<br>115 ->119 (3%)	4.151	298.7	0.0741
S11	108 ->116 (8%)<br>109 ->117 (34%)<br>109 ->118 (15%)<br>111 ->117 (27%)<br>111 ->118 (9%)	4.184	296.3	0.0003
S12	108 ->117 (15%)<br>108 ->118 (4%)<br>109 ->116 (55%)<br>110 ->117 (3%)<br>111 ->116 (9%)<br>112 ->116 (4%)	4.215	294.1	0.0251
S13	109 ->117 (5%)<br>110 ->116 (20%)<br>110 ->117 (23%)<br>110 ->118 (5%)<br>111 ->116 (18%)<br>111 ->117 (8%)<br>112 ->117 (4%)<br>113 ->117 (2%)	4.244	292.1	0.0016
S14	109 ->117 (6%)<br>110 ->116 (19%)<br>110 ->117 (19%)<br>110 ->118 (4%)<br>111 ->116 (13%)<br>111 ->117 (14%)<br>111 ->118 (2%)<br>112 ->117 (6%)<br>113 ->116 (2%)	4.247	291.9	0.0019
S15	109 ->117 (14%)<br>109 ->118 (3%)<br>110 ->116 (38%)<br>111 ->117 (14%)<br>112 ->117 (3%)<br>112 ->118 (5%)<br>114 ->119 (12%)<br>114 ->121 (5%)	4.267	290.6	0.037

Table S8. Lowest-energy vertical triplet excitations of (*OC*-6-33)-[$PtCl_2(tpy)_2$] from TDDFT calculations at the ground state geometry in CH_2Cl_2 solution.

**State**	**monoexcitations**	**AE/eV**	**λ/nm**
T1	114 ->117 (33%)<br>114 ->120 (4%)<br>115 ->116 (41%)<br>115 ->119 (4%)	2.866	432.6
T2	113 ->116 (2%)<br>114 ->116 (37%)<br>114 ->119 (4%)<br>115 ->117 (37%)<br>115 ->120 (4%)	2.869	432.1
T3	112 ->117 (8%)<br>112 ->118 (17%)<br>114 ->116 (4%)<br>115 ->117 (12%)<br>115 ->118 (49%)	3.510	353.2
T4	104 ->120 (4%)<br>105 ->119 (3%)<br>110 ->117 (3%)<br>112 ->116 (10%)<br>113 ->120 (3%)<br>114 ->120 (17%)<br>115 ->116 (15%)<br>115 ->119 (28%)	3.615	343.0

7.3. Complex 5d

Table S9. Fragment contributions (%; from atomic orbital contributions) to the frontier orbitals of **5d** in CH_2Cl_2 solution.

energy (a.u.)	number	L1	L2	L3	L4	Pt
–0.012	134 (LUMO+5)	0	96	0	0	3
–0.021	133 (LUMO+4)	24	31	1	7	36
–0.046	132 (LUMO+3)	97	2	0	0	1
–0.064	131 (LUMO+2)	2	96	0	0	2
–0.067	130 (LUMO+1)	79	5	5	0	11
–0.075	129 (LUMO)	37	11	13	5	34
–0.228	128 (HOMO)	94	1	0	1	3
–0.242	127 (HOMO–1)	80	7	0	8	4
–0.242	126 (HOMO–2)	16	15	5	58	7
–0.243	125 (HOMO–3)	10	57	0	25	8
–0.253	124 (HOMO–4)	0	54	2	42	1
–0.257	123 (HOMO–5)	6	10	54	20	11

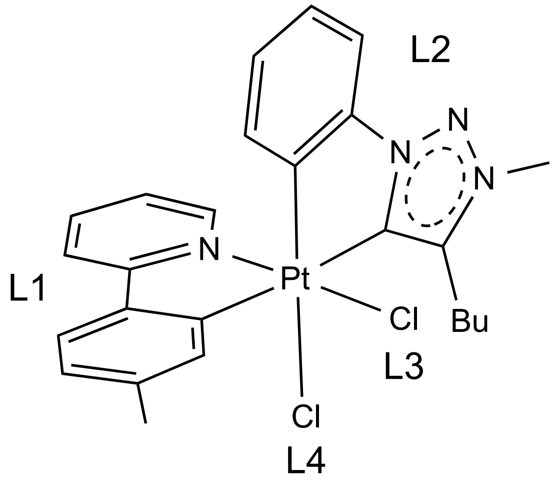

Figure S39. Ligand numbering in complex **5d**.

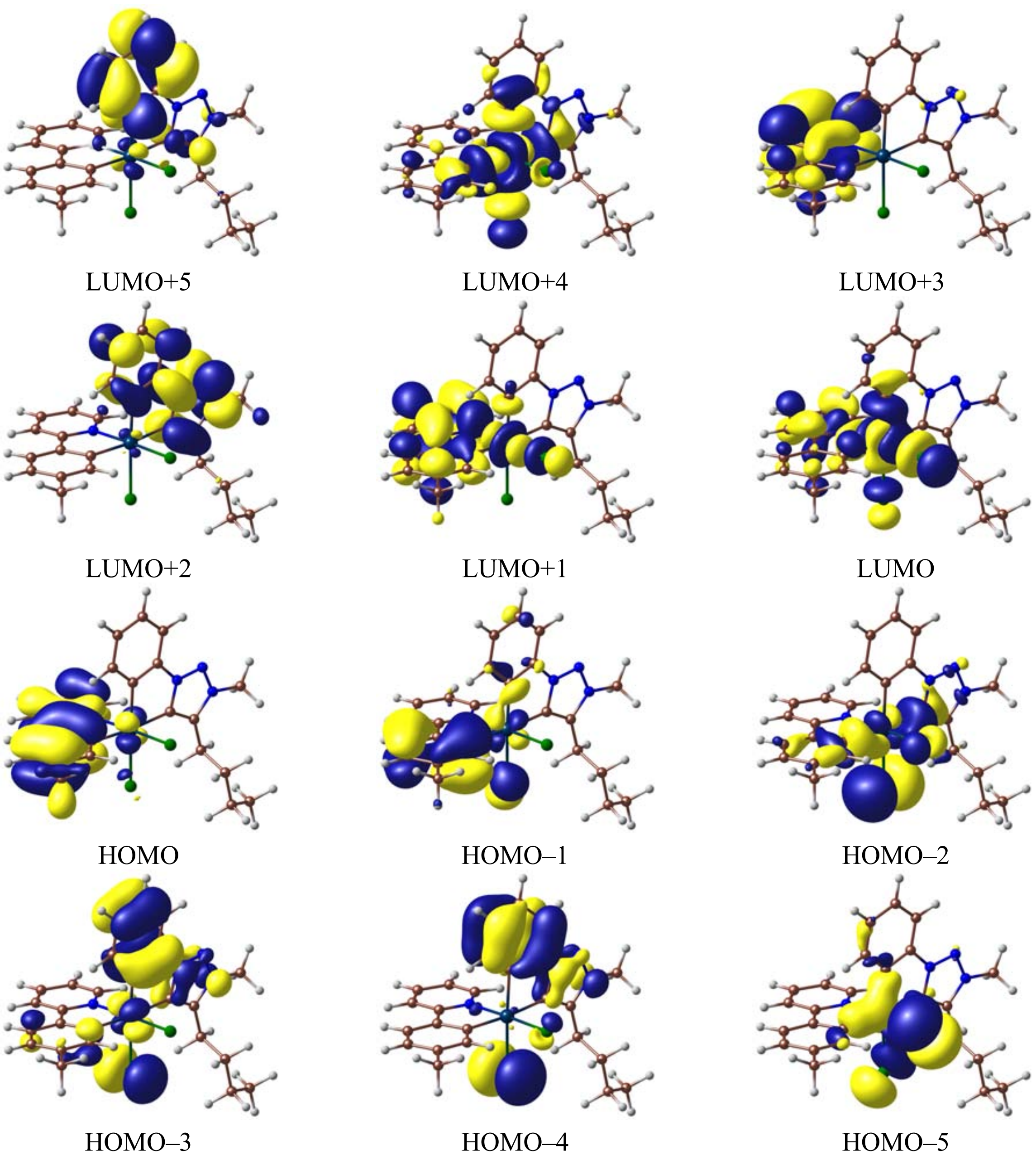

Figure S40. Molecular orbital isosurfaces of **5d** (0.03 e bohr^{-3}).

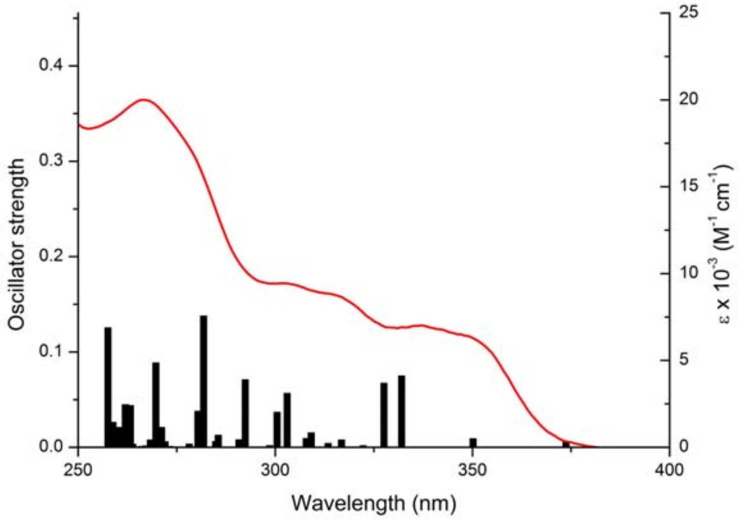

Figure S41. Calculated stick absorption spectrum of **5d** compared with the experimental spectrum in CH_2Cl_2 solution (*ca.* 1×10^{-5} M) at 298 K.

Table S10. Selected vertical singlet excitations of **5d** from TDDFT calculations at the ground state geometry in CH_2Cl_2 solution.

State	monoexcitations	AE/eV	λ/nm	Oscillator strength
S1	125 ->129 (7%)<br>128 ->129 (78%)<br>128 ->130 (6%)	3.318	373.7	0.0064
S2	123 ->129 (5%)<br>125 ->129 (27%)<br>125 ->130 (3%)<br>126 ->129 (46%)<br>126 ->130 (4%)<br>127 ->129 (7%)	3.506	353.6	0.0005
S3	122 ->129 (3%)<br>125 ->129 (21%)<br>125 ->130 (3%)<br>126 ->129 (38%)<br>126 ->130 (3%)<br>127 ->129 (15%)<br>128 ->129 (11%)	3.541	350.2	0.0092
S4	122 ->129 (3%)<br>125 ->129 (6%)<br>126 ->129 (5%)<br>127 ->129 (15%)<br>128 ->130 (63%)	3.734	332.0	0.075
S5	123 ->129 (2%)<br>125 ->129 (12%)<br>125 ->130 (2%)<br>127 ->129 (45%)<br>127 ->130 (6%)<br>128 ->129 (7%)<br>128 ->130 (16%)	3.785	327.6	0.0674
S6	121 ->129 (4%)<br>122 ->129 (15%)<br>122 ->130 (2%)<br>123 ->129 (49%)	3.847	322.3	0.0019

	123 ->130 (7%)<br>124 ->129 (3%)<br>125 ->129 (7%)<br>127 ->129 (5%)			
S7	121 ->129 (6%)<br>122 ->129 (45%)<br>122 ->130 (7%)<br>123 ->129 (14%)<br>123 ->130 (2%)<br>124 ->129 (8%)<br>125 ->129 (5%)<br>127 ->129 (5%)	3.914	316.8	0.0079
S8	128 ->131 (96%)	3.956	313.4	0.0043
S9	126 ->129 (5%)<br>126 ->130 (86%)<br>128 ->130 (2%)	4.011	309.1	0.0153
S10	121 ->129 (6%)<br>123 ->129 (5%)<br>124 ->129 (73%)<br>124 ->130 (4%)<br>125 ->129 (4%)	4.028	307.8	0.0095

Table S11. Lowest-energy vertical triplet excitations of **5d** from TDDFT calculations at the ground state geometry in CH_2Cl_2 solution.

**State**	**monoexcitations**	**AE/eV**	**λ/nm**
T1	127 ->136 (2%)<br>128 ->129 (35%)<br>128 ->130 (43%)<br>128 ->132 (8%)	2.826	438.8
T2	122 ->129 (3%)<br>123 ->129 (11%)<br>123 ->130 (2%)<br>125 ->129 (17%)<br>126 ->129 (8%)<br>128 ->129 (25%)<br>128 ->130 (19%)	3.112	398.4
T3	120 ->131 (3%)<br>121 ->131 (3%)<br>121 ->134 (3%)<br>123 ->129 (3%)<br>124 ->131 (24%)<br>125 ->129 (2%)<br>125 ->131 (28%)<br>125 ->134 (3%)<br>126 ->129 (11%)	3.229	383.9
T4	119 ->129 (2%)<br>123 ->129 (12%)<br>123 ->130 (3%)<br>124 ->131 (6%)<br>125 ->129 (10%)<br>125 ->131 (8%)<br>126 ->129 (32%)<br>126 ->130 (4%)<br>127 ->129 (5%)	3.242	382.4

7.4. Supplementary computational data

Table S12. Energies, free energies, enthalpies and entropies of the optimized structures in CH_2Cl_2 solution.[a]

Structure	E_0 [b]	ZPE [c]	G [d]	H [e]	S [f]
**3d** (S_0)	-2227.001644	-2226.537181	-2226.601643	-2226.504893	203.627
**3d** (T_1)	-2226.899408	-2226.439550	-2226.506113	-2226.406532	209.585
(*OC*-6-33)-[$PtCl_2(tpy)_2$] (S_0)	-2075.820918	-2075.441188	-2075.500130	-2075.413500	182.329
(*OC*-6-33)-[$PtCl_2(tpy)_2$] (T_1)	-2075.719983	-2075.344700	-2075.404870	-2075.316375	186.254
**5d** (S_0)	-2226.975464	-2226.511417	-2226.576405	-2226.479072	204.853
**5d** (T_1)	-2226.897587	-2226.436078	-2226.504581	-2226.402685	214.458

[a] Thermal corrections from vibrational calculations at 298.15 K. [b] Electronic energy (Hartrees). [c] Sum of electronic and zero-point energies (Hartrees). [d] Free Energy (Hartrees). [e] Enthalpy (Hartrees). [f] Entropy (cal mol^{-1} K^{-1}).

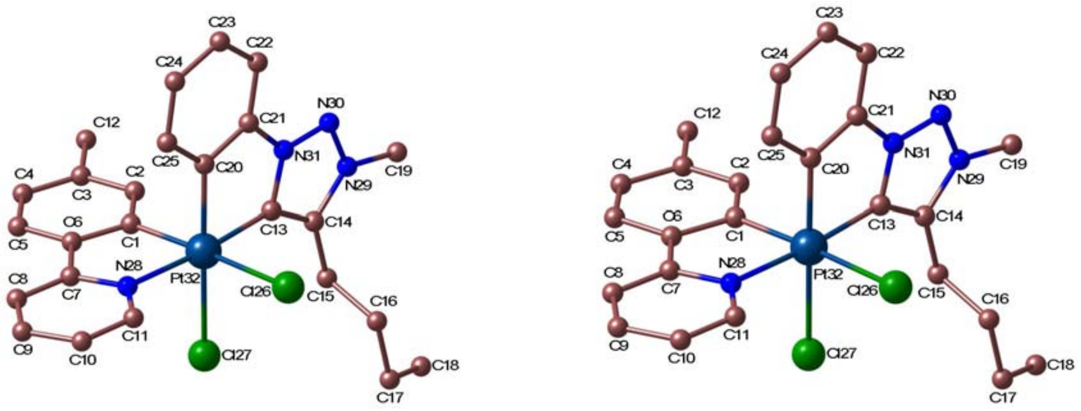

3d (S_0) **3d** (T_1)

Figure S42. Optimized structure of **3d** in the ground state (S_0) and the lowest triplet state (T_1) with atom numbering (hydrogen atoms are omitted).

Table S13. Selected bond lengths (Å) for the optimized geometries of **3d** in the ground state (S_0) and in the lowest triplet state (T_1).

	S_0	T_1
C1-C2	1.391	1.371
C1-C6	1.413	1.481
C1-Pt32	2.033	2.020
C2-C3	1.405	1.421
C3-C4	1.402	1.441
C4-C5	1.390	1.361
C5-C6	1.404	1.455
C6-C7	1.466	1.389
C7-C8	1.401	1.441
C7-N28	1.360	1.415

	S_0	T_1
C8-C9	1.390	1.371
C9-C10	1.397	1.420
C10-C11	1.389	1.404
C11-N28	1.342	1.324
Cl26-Pt32	2.546	2.541
Cl27-Pt32	2.522	2.544
N28-Pt32	2.135	2.117
C13-Pt32	2.013	2.018
C20-Pt32	2.045	2.045

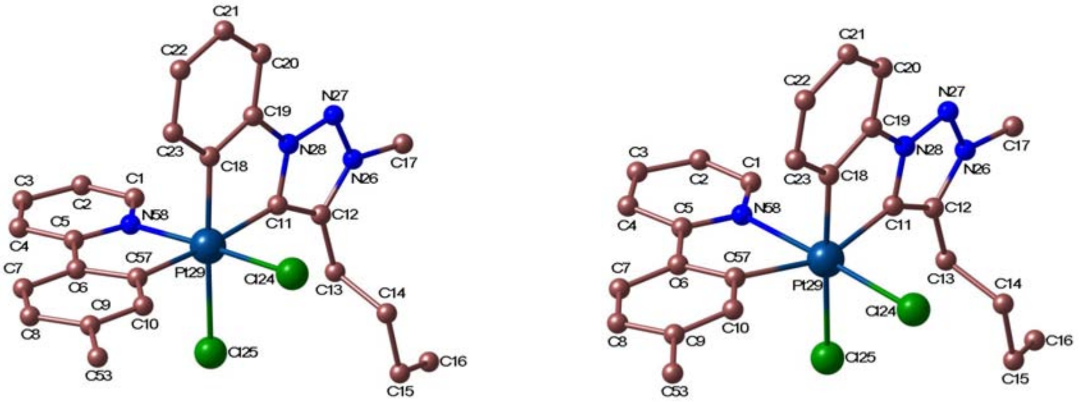

5d (S_0) **5d** (T_1)

Figure S43. Optimized structure of **5d** in the ground state (S_0) and the lowest triplet state (T_1) with atom numbering (hydrogen atoms are omitted).

Table S14. Selected bond lengths (Å) and angles (°) for the optimized geometries of **5d** in de ground state (S_0) and in the lowest triplet state (T_1).

	**S_0**	**T_1**
C1-C2	1.385	1.393
C1-N58	1.346	1.335
C2-C3	1.398	1.396
C3-C4	1.387	1.391
C4-C5	1.403	1.405
C5-C6	1.459	1.473
C5-N58	1.366	1.349
C6-C7	1.405	1.405
C6-C57	1.414	1.408
C7-C8	1.389	1.392
C8-C9	1.405	1.402
C9-C10	1.404	1.405
C9-C53	1.510	1.509
C10-C57	1.393	1.396
C11-C12	1.389	1.390
C11-C28	1.371	1.366
C11-Pt29	2.127	2.071
Cl24-Pt29	2.410	2.635
Cl25-Pt29	2.535	2.478

	**S_0**	**T_1**
C26-C27	1.325	1.328
C27-C28	1.323	1.319
Pt29-C57	2.064	2.084
Pt29-N58	2.070	2.533
C11-Pt29-C18	80.0	79.0
C11-Pt29-Cl24	86.9	93.3
C11-Pt29-Cl25	95.3	98.5
C11-Pt29-C57	174.9	155.2
C11-Pt29-N58	98.0	87.0
C18-Pt29-Cl24	88.8	88.8
C18-Pt29-Cl25	175.2	177.2
C18-Pt29-C57	95.2	88.2
C18-Pt29-N58	91.1	93.7
Cl24-Pt29-Cl25	92.2	90.1
Cl24-Pt29-C57	94.8	107.7
Cl24-Pt29-N58	175.1	177.5
Cl25-Pt29-C57	89.4	94.6
Cl25-Pt29-N58	88.3	87.4
C57-Pt29-N58	80.3	72.6

Table S15. Cartesian coordinates (Å) of the optimized structures at the B3LYP(6-31G**+LANL2DZ) level in CH_2Cl_2 solution.

3d (S_0)

C	35.252118033	10.969010706	-3.979634320
C	35.372003086	11.576303354	-5.225488462
C	34.241831721	11.855260993	-6.012074379
C	32.977665956	11.506591819	-5.516026801
C	32.843232286	10.902432217	-4.271090015
C	33.973324962	10.627137226	-3.485055664
C	33.908553032	9.979777975	-2.171523229
C	32.743216146	9.544312143	-1.527480882
C	32.831317437	8.936550273	-0.280224228
C	34.082367068	8.764693030	0.316662468
C	35.207210306	9.213203822	-0.363363451
C	34.387958462	12.539582851	-7.349516006
C	38.264864291	11.253681290	-3.833052095
C	39.206209920	10.947573577	-4.806668244
C	39.506818158	9.681111341	-5.541463029
C	40.555559675	8.800565744	-4.823489339
C	40.857023170	7.490660120	-5.567731811
C	41.575038590	7.669084771	-6.910880720
C	41.027394061	12.349819149	-5.913517445
C	36.772775640	12.376761997	-1.982981841
C	37.740750580	13.220235553	-2.547093314
C	37.919956032	14.547353143	-2.168859208
C	37.081947078	15.059433734	-1.177980942
C	36.103663977	14.246283410	-0.599165105
C	35.945949048	12.912274873	-0.998048194
Cl	38.532616919	9.810588314	-1.008098016
Cl	36.819790446	8.156548565	-3.727674834
N	35.113142382	9.804929187	-1.564738612
N	39.924655403	12.105418319	-4.984498820
N	39.516080665	13.104533858	-4.215767036
N	38.521098635	12.567303484	-3.535286116
Pt	36.774953628	10.471632406	-2.727549492
H	35.347392164	12.302373805	-7.818087378
H	33.587941524	12.247820446	-8.035888849
H	34.339836512	13.630105293	-7.237800021
H	32.092136091	11.709020585	-6.111884617
H	31.851287945	10.640750416	-3.916233218
H	31.779163203	9.677507485	-2.002632738
H	31.930983362	8.596892459	0.221718908
H	34.190574343	8.292456152	1.285936678
H	36.212634326	9.113095869	0.030525091
H	36.350929720	11.835756790	-5.614712326
H	38.689095002	15.155094481	-2.632759564
H	37.193844623	16.090719440	-0.859729971
H	35.454156743	14.650796659	0.171540738
H	35.180910408	12.302725496	-0.530343534
H	39.836219513	9.922161791	-6.557528115
H	38.573576455	9.118566292	-5.621027527
H	41.487545323	9.365160879	-4.684810742
H	40.173944666	8.571720291	-3.823062329
H	39.918774868	6.941385989	-5.719409303
H	41.474642924	6.863113742	-4.913773577
H	41.809355683	6.698525488	-7.360075050
H	40.966445542	8.222030863	-7.634412495
H	42.518745408	8.212831487	-6.784559549
H	41.824059085	11.626969271	-5.735576198
H	40.670252229	12.265105280	-6.941409696
H	41.392452331	13.358162534	-5.729348681

3d (T_1)

$\langle S^2 \rangle$ = 2.028427

C	35.275494862	10.973811137	-3.987967745
C	35.373473617	11.583866937	-5.211806706
C	34.227704170	11.872250334	-6.001992653
C	32.931019670	11.532798740	-5.474179941
C	32.780459217	10.942640745	-4.257040771
C	33.943898315	10.623946234	-3.443280348
C	33.888146105	10.016532246	-2.195636566
C	32.697859781	9.594351408	-1.500920021
C	32.787678941	8.983832790	-0.276986793
C	34.067247098	8.773399054	0.302242552
C	35.208120491	9.204854992	-0.394079255
C	34.345009854	12.497196971	-7.358568059
C	38.276659281	11.248927108	-3.843560688
C	39.222052270	10.946243807	-4.814386803
C	39.524201896	9.681620939	-5.551732772
C	40.567662330	8.797837782	-4.829842652
C	40.875198556	7.491059331	-5.576977066
C	41.605257257	7.674915457	-6.912849365
C	41.052633328	12.348715709	-5.905525281
C	36.788911049	12.366546018	-1.982691751
C	37.759410534	13.209143733	-2.543678311
C	37.942227833	14.533561091	-2.15760502
C	37.105844564	15.042539274	-1.163624342
C	36.124610770	14.229685024	-0.58946895
C	35.962595006	12.898312690	-0.995642925
Cl	38.535085108	9.771647218	-1.037907539
Cl	36.805721316	8.143414968	-3.776332301
N	35.141263866	9.800419697	-1.574605847
N	39.945403385	12.102515941	-4.982416804
N	39.537162240	13.098270040	-4.209144388
N	38.537263625	12.559874206	-3.536422677
Pt	36.779851481	10.466539445	-2.738657642
H	35.376585285	12.773948258	-7.589340564
H	33.995991000	11.810690828	-8.142706135
H	33.720870796	13.396855339	-7.437506064
H	32.052685027	11.760744909	-6.072356611
H	31.788623728	10.706515136	-3.888607250
H	31.731669809	9.762226435	-1.961541981
H	31.892444441	8.662434077	0.244432746
H	34.182857774	8.288565429	1.263839818
H	36.207256191	9.069191250	0.008612827
H	36.346367206	11.847660314	-5.613537764
H	38.712829607	15.141843936	-2.618348395
H	37.221086457	16.071689538	-0.839665517
H	35.476158398	14.632219363	0.183156607
H	35.194871480	12.287674425	-0.533955915
H	39.859478493	9.924565844	-6.565458842
H	38.590552938	9.120511918	-5.636939728
H	41.498577399	9.361788168	-4.681436956
H	40.178294715	8.564675830	-3.833448682

H	39.938155561	6.942810217	-5.739085225
H	41.486806169	6.860497754	-4.920254977
H	41.842701311	6.706243909	-7.364485742
H	41.003701578	8.231911184	-7.639162655
H	42.548323824	8.217130470	-6.775550835
H	41.845446206	11.621102548	-5.729737944
H	40.699113345	12.272687120	-6.935367762
H	41.421565176	13.354088980	-5.712975778

(*OC*-6-33)-[$PtCl_2(tpy)_2$] (S_0)

C	1.430680763	0.129468534	-1.635288025
C	-1.428427659	0.115906631	-1.638967241
C	1.939560519	-1.131554645	-2.008575123
C	-1.935287794	1.417403812	-1.835201908
C	2.949905576	-1.199881641	-2.980343777
C	-2.939988207	1.621874070	-2.793382714
C	3.434734543	-0.037301082	-3.568623880
C	-3.423558695	0.552790207	-3.539426854
C	2.927104831	1.220199343	-3.208009932
C	-2.920024122	-0.743386347	-3.353660318
C	1.918353133	1.285373734	-2.231463263
C	-1.915530861	-0.945200522	-2.391120351
C	-1.373600763	2.469624140	-0.991739618
C	1.373372942	-2.291438005	-1.324353184
C	-1.745244109	3.819043297	-0.993661230
C	1.740725846	-3.628127597	-1.517975478
C	-1.127057623	4.708664486	-0.123316541
C	1.118048867	-4.630432144	-0.783911180
C	-0.139968588	4.245514148	0.749911621
C	0.130276077	-4.293057543	0.144481109
C	0.197564439	2.900052845	0.720153656
C	-0.203915650	-2.956059108	0.305221388
H	3.365259259	-2.158097785	-3.276583186
H	-3.351701606	2.612895885	-2.957348094
H	4.219044659	-0.103321537	-4.317233694
H	-4.203390162	0.723676273	-4.276001436
H	1.532011301	2.257970540	-1.945660785
H	-1.531740537	-1.948859614	-2.241499506
H	-2.517913935	4.161439530	-1.670751484
H	2.512812592	-3.873454339	-2.236641638
H	-1.414640951	5.754958637	-0.123047883
H	1.402446601	-5.667110134	-0.931865551
H	0.361490364	4.907378009	1.445730649
H	-0.374736837	-5.045509824	0.738197082
H	0.948841002	2.463350987	1.367203312
H	-0.955484219	-2.613406097	1.006302258
N	-0.401105709	2.049135824	-0.132520832
N	0.399416416	-1.994498553	-0.416517621
Pt	-0.000135441	0.021997092	-0.198657286
Cl	-1.813119728	-0.260681938	1.569963607
Cl	1.813978445	0.055964645	1.590471988
C	-3.423166914	-1.896401659	-4.187120502
H	-4.454957148	-1.733523288	-4.510700441
H	-2.812698609	-2.019406842	-5.090525705
H	-3.379185924	-2.838895180	-3.634003311
C	3.432284744	2.477301745	-3.872689249
H	4.483697722	2.381584419	-4.158357221
H	2.864293957	2.689345974	-4.787206254
H	3.330347605	3.345954476	-3.216308177

(*OC*-6-33)-[$PtCl_2(tpy)_2$] (T_1)

$\langle S^2 \rangle = 2.026452$

C	1.423574029	0.155829632	-1.618315125
C	-1.433322522	0.125757921	-1.625868808
C	1.936395251	-1.172684169	-2.003676455
C	-1.950078351	1.424175291	-1.816886464
C	2.967781056	-1.227031773	-3.024318914
C	-2.961238250	1.621240713	-2.769715647
C	3.428541449	-0.078055555	-3.591513363
C	-3.439942340	0.549175630	-3.515082134
C	2.920140350	1.218157116	-3.217589040
C	-2.924248581	-0.742896398	-3.335333116
C	1.903666585	1.285274704	-2.227270623
C	-1.913127195	-0.938225833	-2.378156959
C	-1.389879568	2.481548181	-0.977542681
C	1.386239797	-2.273285173	-1.361310389
C	-1.773923114	3.827485251	-0.976754567
C	1.719665523	-3.655455882	-1.576897406
C	-1.155520119	4.722821993	-0.112174624
C	1.111866013	-4.642903893	-0.847065929
C	-0.156266466	4.268797090	0.751869111
C	0.140909824	-4.285386563	0.128069130
C	0.192485218	2.925995421	0.719254050
C	-0.180902120	-2.935188955	0.312764865
H	3.370618020	-2.185497218	-3.331591986
H	-3.381427423	2.609178325	-2.930831223
H	4.202923597	-0.128612162	-4.352191736
H	-4.225041418	0.715063691	-4.247199001
H	1.521258499	2.262909510	-1.953110891
H	-1.516866936	-1.937644162	-2.232786772
H	-2.556174186	4.162995401	-1.646274602
H	2.463300615	-3.901208326	-2.325729014
H	-1.452630914	5.766474540	-0.109254875
H	1.367014030	-5.684454871	-1.007937601
H	0.345537916	4.935514903	1.442836293
H	-0.355850175	-5.032437791	0.734749682
H	0.952956556	2.495107930	1.359820850
H	-0.918774383	-2.614934440	1.040180831
N	-0.406174880	2.069649591	-0.127462781
N	0.395465041	-1.966528472	-0.391009918
Pt	0.001968670	0.036996601	-0.192577425
Cl	-1.788788391	-0.246352357	1.587200567
Cl	1.851078670	0.095492602	1.599567873
C	-3.420947767	-1.898764277	-4.168766381
H	-4.448397175	-1.734881464	-4.505190579
H	-2.800009087	-2.028673407	-5.064038277
H	-3.385985862	-2.838444628	-3.610135454
C	3.474410179	2.452236995	-3.860035282
H	4.537657423	2.581232327	-3.614117746
H	3.417276763	2.388970214	-4.954461222
H	2.942456570	3.350730031	-3.538762048

5d (S_0)

C	35.307245867	11.368915774	-5.213948138
C	34.225525124	11.638449295	-6.036516018
C	32.941657561	11.349165556	-5.565377078
C	32.787074356	10.802096983	-4.300068195
C	33.908853182	10.543915637	-3.497796892

C	33.888937119	9.960640968	-2.160287700
C	32.710930671	9.590764539	-1.488969293
C	32.782090141	9.036579326	-0.217020088
C	34.023103079	8.834650315	0.409602019
C	35.192341642	9.214221377	-0.269342621
C	38.293829337	11.205010424	-3.907910356
C	39.269758234	10.955272841	-4.863949269
C	39.598524417	9.709549009	-5.623864129
C	40.648042766	8.825316634	-4.912342115
C	40.954313254	7.520295739	-5.663333131
C	41.659807705	7.708470851	-7.011756244
C	41.104720880	12.414968171	-5.877888311
C	36.752938638	12.311613875	-2.003989617
C	37.726784905	13.146080252	-2.577238282
C	37.897049325	14.479312221	-2.211060818
C	37.057564019	15.006550194	-1.230243149
C	36.078625320	14.201360865	-0.643683718
C	35.926209996	12.862250266	-1.026364521
Cl	38.420894796	9.815218350	-1.109670313
Cl	36.807724834	8.085588806	-3.774766878
N	39.979293642	12.125944099	-4.990424367
N	39.534985099	13.091602036	-4.199534879
N	38.528844891	12.511532488	-3.565121261
Pt	36.725898315	10.390992302	-2.722830818
H	32.071256873	11.547140713	-6.182345520
H	31.799556530	10.566938498	-3.923135235
H	31.737990514	9.734401819	-1.949512655
H	31.867666140	8.755160484	0.298292505
H	36.153588093	9.066969234	0.213643601
H	36.324806461	11.564106506	-5.524025051
H	38.666282065	15.081837934	-2.681432922
H	37.169702499	16.042386401	-0.926723737
H	35.427154839	14.613104369	0.121605824
H	35.166803442	12.252380366	-0.551382093
H	39.935859726	9.971549057	-6.632443897
H	38.672559271	9.137052834	-5.724471360
H	41.578434903	9.390906463	-4.767366287
H	40.265598431	8.587657628	-3.913937864
H	40.019206942	6.963894120	-5.809193696
H	41.581922234	6.895181883	-5.016523923
H	41.899870764	6.741173946	-7.464972586
H	41.039936620	8.257173365	-7.728887991
H	42.599163099	8.261173151	-6.891996236
H	41.921414734	11.721289771	-5.674686841
H	40.788397930	12.320263065	-6.918218291
H	41.426952676	13.435267628	-5.680044707
H	34.392321634	12.062667677	-7.019302947
C	34.099043242	8.197958378	1.776169445
H	33.223425078	8.446284645	2.383597107
H	34.137850720	7.104326861	1.694593417
H	34.995998626	8.514982753	2.315645298
C	35.147540226	9.773353921	-1.544736056
N	35.149180358	10.841749610	-3.985445612

5d (T_1)
$\langle S^2 \rangle$ = 2.006824

C	35.386065866	11.168286846	-5.457082859
C	34.312316861	11.423003457	-6.306676932
C	33.023951397	11.204662966	-5.816149149
C	32.856584910	10.741654411	-4.515248037
C	33.986290017	10.495537723	-3.717757221
C	33.933204384	9.995681604	-2.332751514
C	32.742888585	9.555942295	-1.730041128
C	32.729583640	9.101649818	-0.414456176
C	33.901137830	9.072851251	0.355690216
C	35.090983191	9.532298973	-0.232689722
C	38.338683618	11.160914877	-3.708113098
C	39.185099188	10.930133588	-4.786058362
C	39.505915541	9.683177831	-5.544149873
C	40.726108634	8.927878963	-4.966713151
C	41.008696800	7.597720668	-5.681989540
C	41.446355648	7.738542989	-7.144678901
C	40.771445631	12.471859699	-6.059628152
C	36.957568834	12.164287171	-1.692359294
C	37.803367542	13.072631191	-2.351996462
C	37.957765853	14.409267039	-1.992503597
C	37.227219938	14.880796406	-0.903956304
C	36.378609341	14.011125137	-0.214794915
C	36.245426064	12.671929023	-0.602798102
Cl	38.819235933	9.608384660	-0.672119946
Cl	37.120807421	7.942023759	-3.400792760
N	39.790102986	12.138849885	-5.027066076
N	39.387545681	13.100530368	-4.205176929
N	38.517712430	12.485626087	-3.427609669
Pt	36.979732259	10.227311376	-2.454364261
H	32.156444197	11.396736527	-6.439582391
H	31.860205693	10.582017669	-4.121547471
H	31.813892894	9.558390004	-2.290895731
H	31.791846153	8.774085291	0.025768909
H	36.006388339	9.535430485	0.350593136
H	36.410779084	11.322009450	-5.781936993
H	38.633551923	15.052542047	-2.545477196
H	37.323112253	15.916782423	-0.595765279
H	35.810250856	14.374613338	0.636550001
H	35.578933616	12.030618480	-0.039434701
H	39.668824503	9.928942827	-6.598726416
H	38.629618919	9.032481483	-5.488519268
H	41.618158810	9.566472393	-5.015670789
H	40.535708649	8.736029509	-3.905357647
H	40.115136670	6.963040874	-5.621152254
H	41.792968704	7.073268919	-5.122840517
H	41.689445124	6.761401006	-7.574316216
H	40.662973132	8.181020949	-7.769331209
H	42.338278768	8.370371575	-7.230923151
H	41.667105060	11.863906956	-5.926839959
H	40.341793604	12.291602931	-7.046424921
H	41.017891201	13.525830599	-5.948389855
H	34.485374966	11.782711110	-7.314701117
C	33.895026045	8.550640674	1.771261172
H	32.937946970	8.748885764	2.262806637
H	34.051857618	7.465198756	1.785652074
H	34.692006405	9.003291431	2.367475003
C	35.115511932	9.929517778	-1.571104487
N	35.221615622	10.722261017	-4.210071972

8. References

(1) Sheldrick, G. M. A Short History of SHELX. *Acta Crystallogr., Sect. A Found. Crystallogr.* **2008**, *64*, 112–122.

(2) Spek, A. L. Single-Crystal Structure Validation with the Program PLATON. *J. Appl. Crystallogr.* **2003**, *36*, 7–13.

(3) van der Sluis, P.; Spek, A. L. BYPASS: An Effective Method for the Refinement of Crystal Structures Containing Disordered Solvent Regions. *Acta Crystallogr. Sect. A Found. Crystallogr.* **1990**, *46*, 194–201.

(4) Cardona, C. M.; Li, W.; Kaifer, A. E.; Stockdale, D.; Bazan, G. C. Electrochemical Considerations for Determining Absolute Frontier Orbital Energy Levels of Conjugated Polymers for Solar Cell Applications. *Adv. Mater.* **2011**, *23*, 2367–2371.

(5) Frisch, M. J.; Trucks, G. W.; Schlegel, H. B.; Scuseria, G. E.; Robb, M. A.; Cheeseman, J. R.; Scalmani, G.; Barone, V.; Mennucci, B.; Petersson, G. A.; Nakatsuji, H.; Caricato, M.; Li, X.; Hratchian, H. P.; Izmaylov, A. F.; Bloino, J.; Zheng, G.; Sonnenberg, J. L.; Hada, M.; Ehara, M.; Toyota, K.; Fukuda, R.; Hasegawa, J.; Ishida, M.; Nakajima, T.; Honda, Y.; Kitao, O.; Nakai, H.; Vreven, T.; Montgomery Jr., J. A.; Peralta, J. E.; Ogliaro, F.; Bearpark, M.; Heyd, J. J.; Brothers, E.; Kudin, K. N.; Staroverov, V. N.; Kobayashi, R.; Normand, J.; Raghavachari, K.; Rendell, A.; Burant, J. C.; Iyengar, S. S.; Tomasi, J.; Cossi, M.; Rega, N.; Millam, N. J.; Klene, M.; Knox, J. E.; Cross, J. B.; Bakken, V.; Adamo, C.; Jaramillo, J.; Gomperts, R.; Stratmann, R. E.; Yazyev, O.; Austin, A. J.; Cammi, R.; Pomelli, C.; Ochterski, J. W.; Martin, R. L.; Morokuma, K.; Zakrzewski, V. G.; Voth, G. A.; Salvador, P.; Dannenberg, J. J.; Dapprich, S.; Daniels, A. D.; Farkas, Ö.; Foresman, J. B.; Ortiz, J. V; Cioslowski, J.; Fox, D. J. Gaussian 09 (Revision A.02). Gaussian Inc.: Wallingford CT 2009.

(6) Becke, A. Density Functional Thermochemistry III The Role of Exact Exchange. *J. Chem. Phys.* **1993**, *98*, 5648–5652.

(7) Lee, C. T.; Yang, W. T.; Parr, R. G. Development of the Colle-Salvetti Correlation-Energy Formula into a Functional of the Electron Density. *Phys. Rev. B* **1988**, *37*, 785–789.

(8) Hariharan, P. C.; Pople, J. A. Influence of Polarization Functions on Molecular-Orbital Hydrogenation Energies. *Theor. Chim. Acta* **1973**, *28*, 213–222.

(9) Francl, M. M.; Pietro, W. J.; Hehre, W. J.; Binkley, J. S.; Gordon, M. S.; Defrees, D. J.; Pople, J. A. Self-Consistent Molecular Orbital Methods. XXIII. A Polarization-Type Basis Set for Second-Row Elements. *J. Chem. Phys.* **1982**, *77*, 3654–3665.

(10) Hay, P. J.; Wadt, W. R. Ab Initio Effective Core Potentials for Molecular Calculations–Potentials for K to Au Including the Outermost Core Orbitals. *J. Chem. Phys.* **1985**, *82*, 299–310.

(11) Escudero, D.; Thiel, W. Exploring the Triplet Excited State Potential Energy Surfaces of a Cyclometalated Pt(II) Complex: Is There Non-Kasha Emissive Behavior? *Inorg. Chem.* **2014**, *53*, 11015–11019.

(12) Tomasi, J.; Mennucci, B.; Cammi, R. Quantum Mechanical Continuum Solvation Models. *Chem. Rev.* **2005**, *105*, 2999–3093.

(13) Glendening, E. D.; Badenhoop, J. K.; A. E. Reed; Carpenter, J. E.; Bohmann, J. A.; Morales, C. M.; Weinhold, F. NBO 5.9. Theoretical Chemistry Institute, University of Wisconsin: Madison, WI 2009.
